# Emerging Processing Technologies for Modulating Allergenic Proteins in Legumes and Gluten-Containing Cereals: A Systematic Review

**DOI:** 10.3390/molecules31193438

**Published:** 2026-09-27

**Authors:** Jose Aaron Ortiz-Zapata, Rebeca Salvador-Reyes, Marcio Schmiele, Raniere Ribeiro Lima, Ernesto Dante Rodríguez-Lafitte, Zecarlos Hugo Solano-Santisteban, Bruno Martins Dala-Paula, Luis Miguel Anaya-Esparza, Edward Florencio Aurora-Vigo

**Affiliations:** 1 Escuela de Ingeniería Agroindustrial y Comercio Exterior, Facultad de Ingeniería, Universidad Señor de Sipán, Chiclayo 14000, Peru; ozapatajoseaaro@uss.edu.pe (J.A.O.-Z.); ssantistebanzec@uss.edu.pe (Z.H.S.-S.); 2 Facultad de Ingeniería, Universidad Tecnológica del Perú, Lima 150101, Peru; rsalvador@utp.edu.pe; 3 Instituto de Ciência e Tecnologia, Universidade Federal dos Vales do Jequitinhonha e Mucuri, Diamantina 39100-000, Brazil; raniere.ribeiro@ufvjm.edu.br; 4 Faculdade de Farmácia, Universidade Federal de Minas Gerais, Belo Horizonte 31270-901, Brazil; brunodala@farmacia.ufmg.br; 5 Universidad de Guadalajara, Centro Universitario de los Altos, Departamento de Ciencias Pecuarias y Agrícolas, Tepatitlán de Morelos 47620, Mexico; luis.aesparza@academicos.udg.mx; 6 Programa de Doctorado en Ingeniería Agroindustrial Mención Transformación Avanzada de Granos y Tubérculos Andinos, Universidad Nacional del Santa, Chimbote 02712, Peru

**Keywords:** food allergy, immunoreactivity, IgE binding, legume proteins, gluten, emerging processing, protein structure

## Abstract

Legumes and gluten-containing cereals are major sources of plant protein, but they also contain immunoreactive proteins that may resist processing and gastrointestinal digestion. This systematic review examined how emerging processing technologies affect these proteins, focusing on immunoreactivity, antigenicity, molecular structure, digestibility, techno-functional properties, and the analytical methods used to measure these responses. Searches were conducted in Scopus and the Web of Science Core Collection according to PRISMA 2020, resulting in 95 eligible studies. Soybean and wheat were the most studied matrices, representing 35% and 30% of the included studies, respectively. Most experiments were conducted in vitro and relied on enzyme-linked immunosorbent assays, Western blotting, electrophoresis, or simulated gastrointestinal digestion. Direct biological evidence was scarce. Ultrasound and irradiation were the most frequently investigated technologies, followed by microwave processing, cold plasma, extrusion, high-pressure processing, electric fields, enzymatic hydrolysis, and pH shifting. These treatments induced protein unfolding, aggregation, fragmentation, oxidation, secondary-structure rearrangement, changes in sulfhydryl-group accessibility, and shifts in digestibility and techno-functional behavior. Decreased antibody recognition was reported more consistently in legumes, especially after combined treatments. Responses in gluten-containing cereals were less predictable and ranged from partial reduction to no change or increased recognition, depending on the matrix, treatment intensity, extraction procedure, and immunological assay. Emerging technologies can therefore modify protein structure and analytical immunoreactivity. Current evidence, however, does not establish that these changes reduce clinical allergenicity or gluten-related immunogenicity.

## 1. Introduction

Growing interest in healthier and more sustainable diets has expanded the consumption of cereals and legumes beyond their traditional forms, increasing their use as flours, protein concentrates, isolates, textured materials, and functional ingredients in novel food products [1,2,3]. This expanding utilization is supported by their nutritional, technological, and agronomic value. Legumes provide substantial amounts of plant proteins, complex carbohydrates, dietary fiber, vitamins, and minerals, while also contributing to more sustainable agricultural systems through biological nitrogen fixation and improvements in soil fertility [4,5,6]. Cereals supply a large share of global dietary energy and provide proteins, B-group vitamins, minerals, dietary fiber, and several bioactive compounds, in addition to exhibiting broad technological versatility [7,8,9].

However, their increasing incorporation into diversified food products also heightens the need to understand how processing affects proteins capable of eliciting adverse immune responses in susceptible individuals.

A critical concern associated with this broader utilization is the presence of proteins capable of triggering distinct adverse immune responses in susceptible individuals. The major allergen families in legumes include vicilins, also known as 7S globulins [10], legumins or 11S globulins [11], and several albumins [12]. In soybean, β-conglycinin (Gly m 5) and glycinin (Gly m 6) are particularly relevant storage allergens [13,14], whereas Ara h 1 and Ara h 2 are among the major allergens in peanuts. Wheat presents a more complex immunological context because gliadin and glutenin fractions may participate in IgE-mediated wheat allergy, while specific gluten-derived peptides are involved in immune responses associated with celiac disease and other gluten-related disorders [15]. Findings related to wheat allergy should therefore be interpreted separately from those concerning gluten immunogenicity, as these conditions involve distinct immune mechanisms and analytical endpoints.

Many of these proteins possess compact molecular structures stabilized by disulfide bonds, extensive hydrophobic interactions, and other non-covalent forces, which may limit their unfolding and enzymatic hydrolysis [16]. Their resistance to processing and gastrointestinal digestion can preserve linear or conformational epitopes during food preparation and gastrointestinal transit, thereby maintaining antibody recognition. Structural stability alone, however, does not determine allergenicity, which also depends on protein abundance, solubility, matrix interactions, digestion kinetics, epithelial exposure, and the immune status of the individual.

Legumes and cereals also contain antinutritional components, including lectins, trypsin inhibitors, phytates, and tannins [17]. Although these compounds are not necessarily allergens, they may affect digestive-enzyme activity, protein hydrolysis, and nutrient bioavailability, thereby modifying gastrointestinal exposure to immunoreactive proteins. Trypsin inhibitors, for example, can reduce proteolytic activity, whereas tannins and phytates may interact with proteins and alter their solubility or accessibility to digestive enzymes. Changes in these compounds should not be interpreted as direct evidence of reduced allergenicity, but they remain relevant when evaluating the overall effect of processing on protein digestion and epitope persistence.

Conventional thermal treatments, including cooking, boiling, and roasting, can inactivate several antinutritional factors and modify protein antigenicity [18], although their effects vary with the food matrix, target protein, moisture content, temperature, and exposure time. Heating may disrupt conformational epitopes and increase susceptibility to proteolysis, but it can also expose previously buried sequences, promote aggregation and disulfide-bond exchange, or generate neoepitopes through oxidation and glycation reactions [19,20]. Consequently, thermal processing may reduce, preserve, or even increase antibody recognition depending on the molecular response of the protein and the analytical method used. Severe heating can also impair nutritional and sensory quality by promoting amino acid losses, browning, off-flavor formation, and undesirable changes in color, aroma, and texture [21].

These limitations have encouraged the investigation of emerging and non-conventional technologies such as ultrasound, high-pressure processing, pulsed electric fields, cold plasma, irradiation, microwave processing, extrusion, and selected biotechnological treatments. These processes expose proteins to different combinations of mechanical, thermal, electrical, oxidative, or enzymatic stresses, leading to unfolding, aggregation, fragmentation, oxidation, hydrolysis, and rearrangements of secondary and tertiary structures. Such molecular changes may alter epitope accessibility, antibody binding, digestive-enzyme susceptibility, solubility, interfacial behavior, gelation, and other techno-functional properties. Although some of these technologies operate under milder bulk-temperature conditions than conventional heating, their effects still depend strongly on treatment intensity, exposure time, hydration, protein concentration, matrix composition, and the heat generated during processing [22,23,24,25].

Experimental findings illustrate this pronounced dependence on processing conditions and analytical approach. Ultraviolet treatment of wheat, for example, reduced the measured gliadin immunoreactivity by approximately 50% under selected conditions [26], while high-pressure processing of whole soymilk decreased antigen-binding capacity by approximately 56% [23]. In white lupin, solid-state fermentation modified the proteomic profile and decreased the abundance of tryptic peptides derived from certain allergenic proteins [27]. These results are relevant, but they do not independently demonstrate hypoallergenicity because a weaker immunoassay signal may reflect epitope destruction, aggregation-induced masking, reduced recovery of insoluble proteins, or interference from other matrix components.

A more reliable assessment requires complementary analytical and biological approaches capable of distinguishing true epitope degradation from reduced protein extractability or impaired antibody access. Antibody-based assays should therefore be combined with structural characterization, protein-recovery measurements, and simulated gastrointestinal digestion. IgE-binding assays using sera from well-characterized sensitized individuals provide greater clinical relevance than assays based on IgG, animal antisera, or generic monoclonal antibodies, although IgE recognition alone still does not reproduce the complexity of an allergic reaction. Cellular assays, animal models, and, when ethically and technically feasible, clinical studies are needed to determine whether processing-induced reductions in analytical immunoreactivity translate into biologically meaningful changes in allergenic potential.

Previous reviews have examined processing strategies for reducing food antigenicity, but many have combined animal and plant-derived matrices without addressing the distinct structural and immunological characteristics of legumes and gluten-containing cereals in sufficient detail [28]. Other reviews have focused specifically on legumes, including allergen detection, characterization, and immunotherapy strategies, but have not comprehensively analyzed the relationships among emerging technology conditions, structural protein modifications, and changes in immunoreactivity [29]. A critical synthesis that compares processing technologies, underlying mechanisms, food matrices, analytical methods, and levels of evidence is therefore still needed.

This systematic review aimed to critically examine the effects of emerging processing technologies on allergenic and immunoreactive proteins in legumes and gluten-containing cereals. Specifically, it sought to: (i) identify the food matrices and target proteins most frequently investigated; (ii) characterize the technologies and their operating conditions; (iii) compare their effects on protein immunoreactivity, antigenicity, digestibility, molecular structure, and techno-functional properties; (iv) assess the in vitro and in vivo methods used and the type of evidence they provide; and (v) identify methodological weaknesses and knowledge gaps that currently limit biological and clinical interpretation.

## 2. Methodology

### 2.1. Review Design and Search Strategy

This systematic review was conducted and reported in accordance with the PRISMA 2020 guidelines [30]. The research question was structured using the PIO framework: Population (P), legumes and gluten-containing cereals; Intervention (I), emerging or non-conventional processing technologies; and Outcomes (O), changes in immunoreactivity, antigenicity, IgE-binding capacity, protein structure, digestibility, and techno-functional properties.

The main research question was: How do emerging processing technologies affect allergenic and immunoreactive proteins in legumes and gluten-containing cereals? The following specific questions were subsequently formulated:

(i) Which food matrices and allergenic proteins have been most frequently investigated?
(ii) Which emerging technologies have been applied, and what are their main operating conditions?
(iii) What effects do these technologies have on protein immunoreactivity, antigenicity, structure, digestibility, and functionality?
(iv) To what extent does the available evidence support reductions in immunoreactivity, and under which processing conditions?
(v) Which in vitro and in vivo methods have been used to assess these effects?

Bibliographic searches were conducted in Scopus and Web of Science Core Collection without publication-year restrictions. Original research articles and conference papers published in English or Spanish were considered. In Scopus, the search terms were applied to the title, abstract, and keyword fields, whereas the Topic field was used in Web of Science. The final search was performed on 21 February 2026 and identified 639 records. The Boolean structure used in Scopus was adapted to the Web of Science Topic field while preserving the same conceptual groups and Boolean operators.

The complete Scopus search string was: (TITLE-ABS-KEY ((“legumes” OR “grain legumes” OR Fabaceae OR soybean OR “Glycine max” OR pea OR “Pisum sativum” OR lentil OR “Lens culinaris” OR chickpea OR “Cicer arietinum” OR lupin OR “Lupinus mutabilis” OR “Lupinus albus” OR “Lupinus angustifolius” OR “Vicia faba” OR “Phaseolus vulgaris” OR “Vigna radiata” OR “Vigna angularis” OR “mung bean” OR “adzuki bean” OR “faba bean” OR “common bean” OR wheat OR “Triticum aestivum” OR barley OR “Hordeum vulgare” OR rye OR “Secale cereale” OR triticale OR oat* OR “Avena sativa” OR gluten)) AND TITLE-ABS-KEY ((“high pressure processing” OR HPP OR ultrasound OR ultrasonics OR “ultrasound-assisted” OR irradiation OR microwave OR “microwave-assisted” OR “pulsed electric fields” OR PEF OR “cold plasma” OR “non-thermal plasma” OR “pulsed light” OR “ohmic heating” OR ohmic OR “enzymatic hydrolysis” OR “assisted extrusion” OR “microwave-assisted extrusion” OR “ultrasound-assisted extrusion” OR “steam-assisted extrusion” OR co-extrusion OR coextrusion OR “third-generation extrusion” OR “3G extrusion” OR “extrusion cooking” OR fermentation OR “controlled fermentation” OR germination)) AND TITLE-ABS-KEY ((allergen* OR allergenicity OR hypoallergenic OR “IgE binding” OR “allergen reduction” OR “protein allergen*” OR vicilin OR legumin OR glycinin OR “β-conglycinin” OR “Ara h” OR “Gly m” OR “Lup an” OR “Len c” OR “Cic a”))) AND (LIMIT-TO (DOCTYPE, “ar”) OR LIMIT-TO (DOCTYPE, “cp”)).

### 2.2. Study Selection and Eligibility Criteria

**Figure 1:**
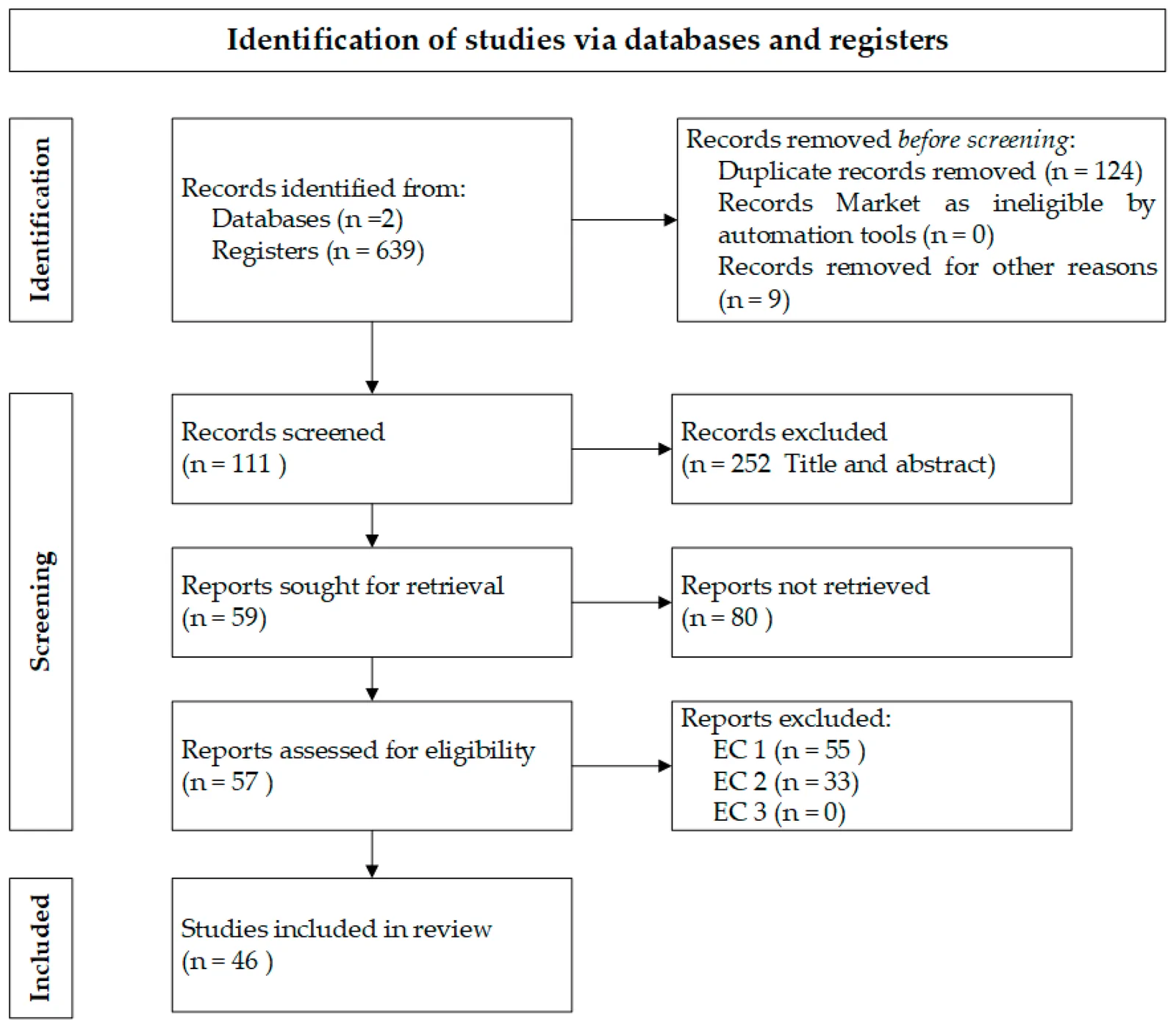
PRISMA 2020 flow diagram for study identification, screening, eligibility, and inclusion.

All records retrieved from the two databases were exported to a standardized Microsoft Excel form and checked for duplication. After the removal of 124 duplicate records, the titles and abstracts of 515 records were screened by two reviewers. Disagreements regarding eligibility were resolved through discussion until consensus was reached. At this stage, 252 records were excluded because they did not address the food matrices, processing technologies, or outcomes defined for the review. The full texts of the remaining 263 reports were sought for eligibility assessment, but 80 could not be retrieved. The other 183 reports were assessed against the predefined inclusion and exclusion criteria, resulting in the exclusion of 88 reports and the inclusion of 95 studies in the final synthesis. The complete process of identification, screening, retrieval, eligibility assessment, and inclusion is presented in the PRISMA 2020 flow diagram shown in Figure 1 [30]. Bibliographic information for all included studies is provided in Supplementary Table S1, while the complete extraction matrix is available in Supplementary Table S2.

Studies were eligible when they met the following criteria: (IC 1) original research articles or conference papers reporting an experimental design; (IC 2) studies conducted on legumes or gluten-containing cereals; (IC 3) application of at least one emerging or non-conventional processing technology; (IC 4) evaluation of a recognized allergenic or immunoreactive protein or protein fraction; and (IC 5) reporting of at least one direct immunological outcome or an associated structural, digestive, proteomic, or techno-functional response. Studies without a direct immunological assay were retained only when they investigated processing-induced changes in a protein or protein fraction recognized as allergenic or immunoreactive. These studies were treated as sources of indirect mechanistic evidence and were not interpreted as demonstrating reduced allergenicity.

The exclusion criteria were as follows: (EC 1) studies conducted on food matrices other than legumes or gluten-containing cereals; (EC 2) exclusive use of conventional treatments, such as boiling or traditional drying, the application of an emerging or non-conventional technology; (EC 3) review articles, book chapters, editorials, and other non-experimental document; (EC 4) exclusive use of computational simulations, in silico studies, or theoretical models; and (EC 5) absence of an allergenic or immunoreactive protein as the analytical target or insufficient methodological and outcome data for extraction.

No formal risk-of-bias or methodological-quality assessment tool was applied to the included studies. The review was designed as a descriptive and comparative synthesis of a heterogeneous body of experimental evidence rather than as a meta-analysis of a common effect estimate. Eligibility therefore required clear reporting of the processing conditions, the analytical or immunological methods used to assess protein modifications, and at least one measurable outcome related to immunoreactivity, antigenicity, structure, digestion, or techno-functional behavior. Studies that lacked sufficient information for data extraction were excluded.

These eligibility requirements improved the transparency and interpretability of the extracted evidence but should not be considered equivalent to a formal risk-of-bias assessment. Likewise, publication in a peer-reviewed source indexed in Scopus or Web of Science does not establish methodological quality or eliminate selective reporting. The absence of a structured quality assessment and certainty-of-evidence framework was therefore considered when interpreting the findings and is acknowledged as a limitation of the review.

### 2.3. Data Extraction and Analysis

Data were extracted into a standardized Microsoft Excel form developed for this review. The recorded variables included database, bibliographic information, publication year, country, food matrix, species or variety, plant material or protein fraction, target allergen or immunoreactive protein, processing technology, operating conditions, complementary treatments, analytical methods, measured outcomes, and limitations reported or identified for each study.

The extracted outcomes covered protein immunoreactivity, antigenicity, IgE-binding capacity, molecular and supramolecular structure, gastrointestinal digestibility, and techno-functional properties. Assays that directly measured antibody recognition, including IgE-, IgG-, R5-, and G12-based methods, were classified as direct immunological evidence, although their biological and clinical relevance was interpreted according to the antibody and experimental model used. Cellular, animal, and other biological models were considered a separate and stronger level of evidence because they evaluated responses beyond analytical antibody binding. Structural, proteomic, digestive, and techno-functional measurements were classified as indirect mechanistic evidence and were not interpreted alone as proof of reduced allergenicity or gluten-related immunogenicity.

For direct immunological outcomes, the direction of the response was classified as decreased, increased, unchanged, or mixed and condition-dependent relative to the corresponding untreated control. This classification considered the statistical interpretation reported in the original study, when available, together with the response observed across treatment conditions. Changes detected only by electrophoresis, spectroscopy, chromatography, proteomics, digestibility assays, or functional measurements were not assigned an immunological direction unless the study also included an antibody-based or biological assessment.

Because the included studies differed markedly in food matrix, target protein, processing intensity, analytical method, antibody specificity, outcome unit, and experimental design, the evidence was synthesized narratively and comparatively rather than pooled through meta-analysis. Comparisons were organized according to food matrix, processing technology, molecular changes, type of immunological assessment, and direction of the reported response. Results derived from IgE binding, IgG or monoclonal-antibody recognition, R5/G12 assays, and biological models were interpreted separately because these approaches address different immunological mechanisms and do not provide equivalent evidence of clinical risk.

The 95 included studies generated 129 evidence records because a single study could contribute more than one record when it evaluated multiple matrices, technologies, treatment conditions, target proteins, or immunological outcomes. Study-level counts were used to describe the overall evidence base, whereas record-level counts were used to summarize specific technology–matrix and immunological-response combinations. These units of analysis were kept separate to prevent multiple experiments from the same publication from being counted as independent studies. The complete study-level and record-level extraction matrix is provided in Supplementary Table S2.

Bibliometric analyses and term co-occurrence mapping were performed in VOSviewer version 1.6.19 using the bibliographic metadata retrieved for the included studies. Publication trends, journal distribution, geographical contribution, and relationships among recurrent terms were examined to characterize the development and thematic structure of the research field. These analyses were used to describe the coverage of the literature and were not interpreted as measures of study quality or technological effectiveness.

## 3. Results and Discussion

### 3.1. General Characteristics of the Included Studies

**Figure 2:**
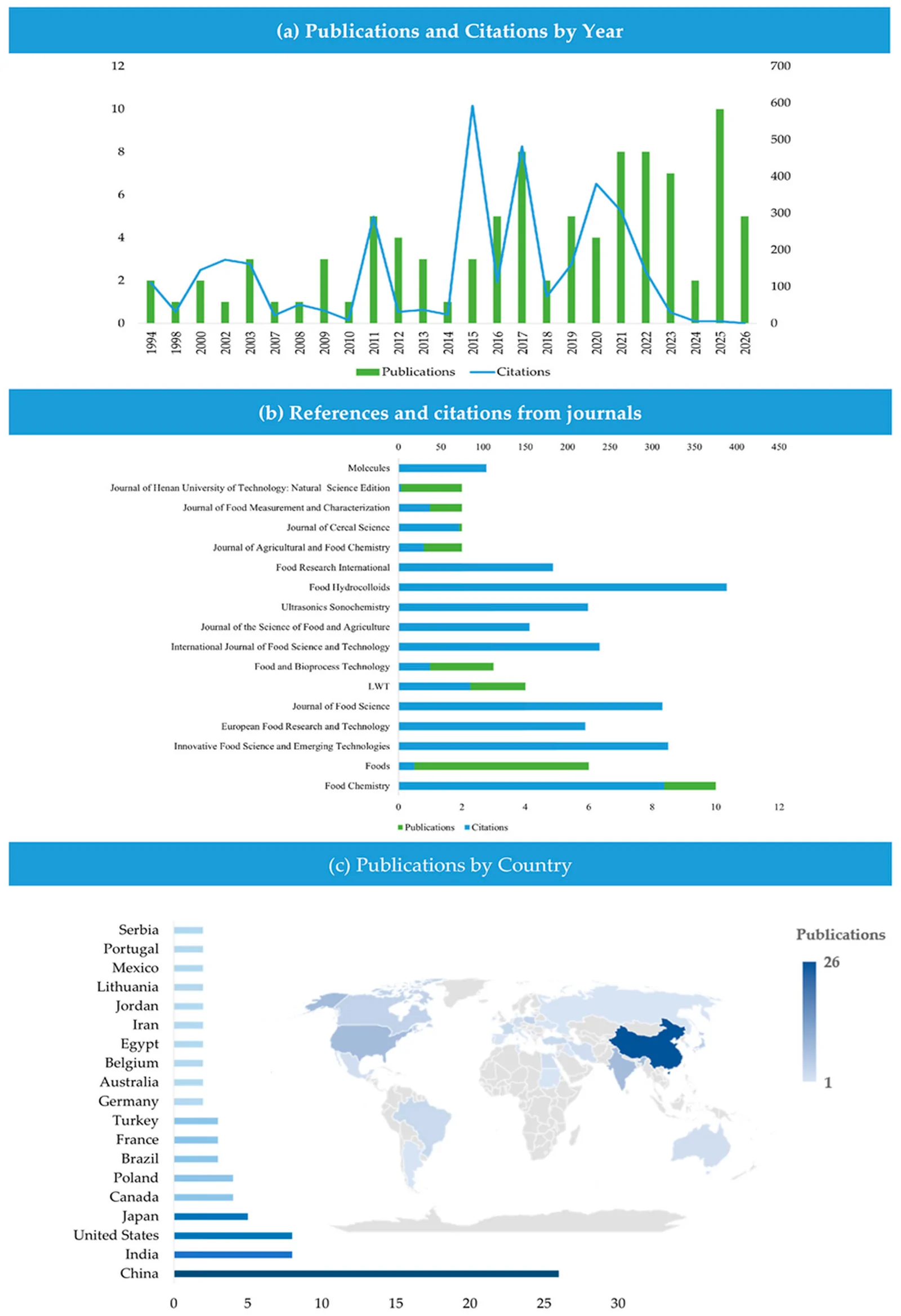
Bibliometric characterization of the studies included in the systematic review: (**a**) annual distribution of publications and citations; (**b**) distribution of publications and citations by journal; and (**c**) geographical distribution of publications.

The included studies show a clear increase in research on the use of emerging technologies to modify allergenic and immunoreactive plant proteins, particularly during the past decade. Earlier publications were scattered across years, whereas recent work has examined a broader range of processing technologies, protein sources, and analytical endpoints. This expansion coincides with the growing use of plant-derived proteins in formulated foods and with the need to control immunoreactivity without compromising nutritional quality or technological performance. The number of publications reported for 2026 is incomplete because the final search was conducted in February. Citation data require similar caution, since recently published studies have had less time to accumulate citations and should not be judged solely on this basis (Figure 2a).

The presence of the included studies in journals focused on food chemistry, processing, protein structure, nutrition, and functional properties reflects the multidisciplinary character of the field (Figure 2b). The geographical distribution, however, was uneven, with most studies originating from Asian and North American countries, while Latin America and Africa remained poorly represented despite their broad diversity of legumes and long history of grain consumption (Figure 2c). This imbalance restricts the extent to which current findings can be transferred across raw materials and production systems, as species, cultivar, environmental conditions, and postharvest practices can alter protein composition, supramolecular organization, and interactions with other matrix components. These factors influence both the susceptibility of proteins to processing and their recovery and recognition during subsequent immunological analysis.

**Figure 3:**
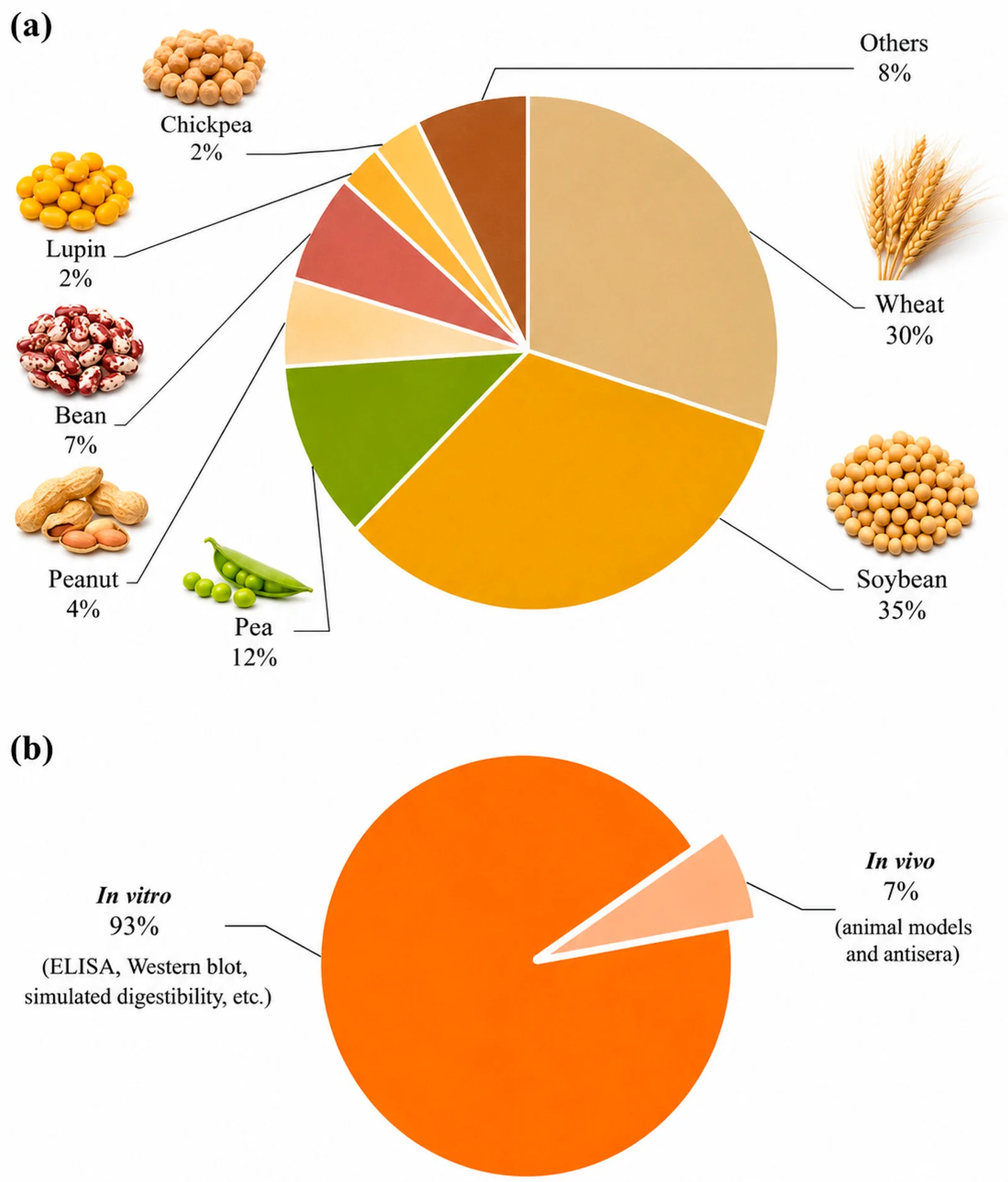
Distribution of the included studies according to: (**a**) the investigated food matrices and (**b**) the assessment approach used (in vitro or in vivo); ELISA: enzyme-linked immunosorbent assay.

The available evidence was also concentrated on a limited number of food matrices, particularly those of greater commercial and clinical relevance (Figure 3a). This pattern creates a bias toward extensively investigated proteins, whereas other legumes and gluten-containing cereals remain poorly characterized. The response of a protein to a given technology cannot be directly extrapolated across species because of differences in composition, supramolecular organization, disulfide-bond content, interactions with other matrix components, and epitope stability.

From a methodological perspective, in vitro studies predominated, whereas in vivo and clinical validation remained scarce (Figure 3b). In vitro assays are widely used because of their reproducibility, accessibility, and lower ethical complexity; however, a reduction in analytical signal does not necessarily demonstrate a decrease in allergenic risk. Human studies are subject to stringent bioethical restrictions because of the risk of inducing severe allergic responses [31]. Therefore, the limited biological validation represents one of the main weaknesses of the available evidence.

**Figure 4:**
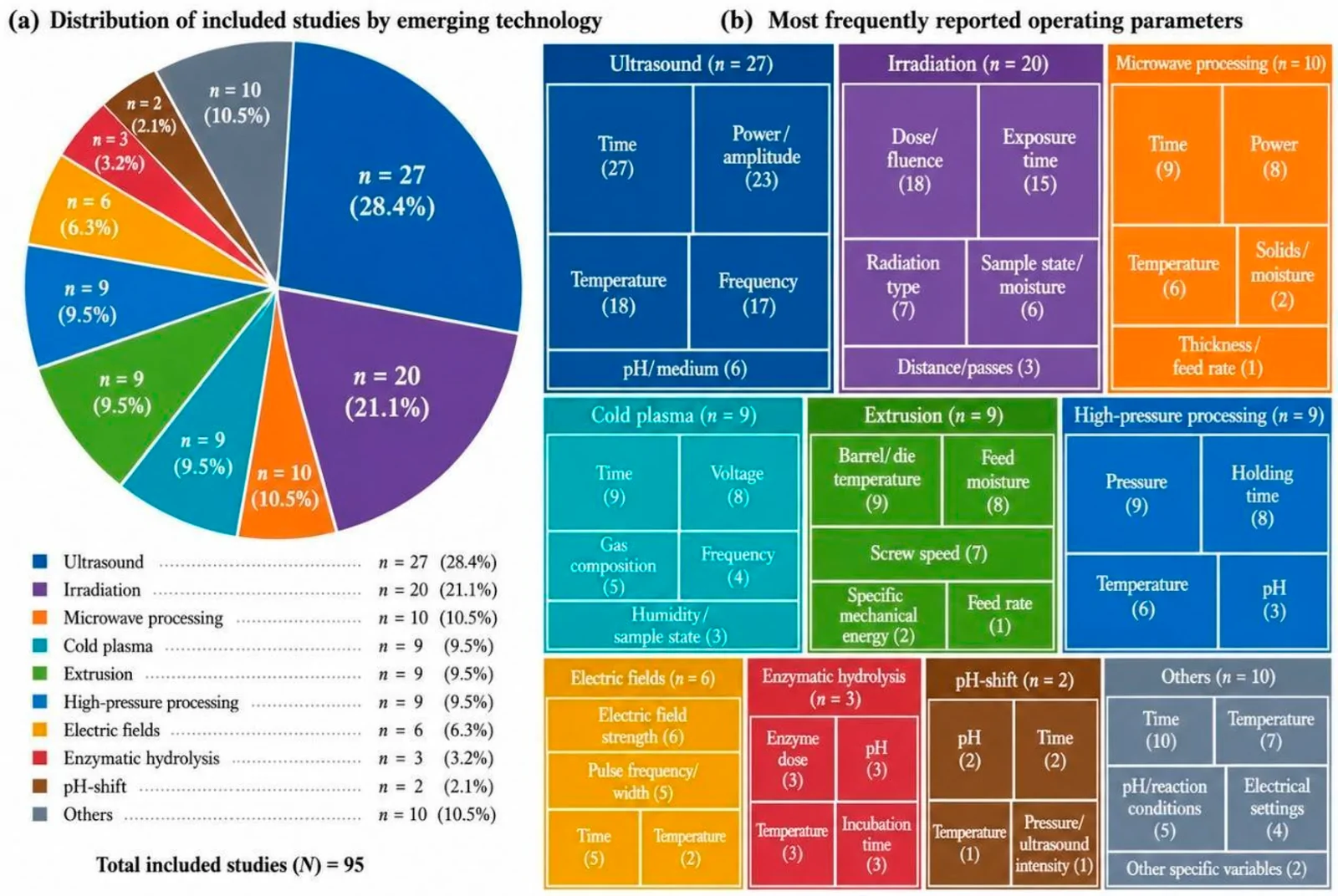
Frequency of emerging processing technologies and operating parameters reported in the included studies: (**a**) distribution of the investigated technologies; and (**b**) tree map of the most frequently reported operating parameters for each technology. Technology categories were not mutually exclusive because some studies evaluated more than one treatment. Note: Percentages are calculated relative to the total number of included studies (*n* = 95). The sum of the individual category counts (*n* = 105) exceeds *n* because several studies evaluated more than one emerging technology; categories are therefore not mutually exclusive, and percentages do not add up to 100%.

The technologies investigated showed considerable heterogeneity in their mechanisms of action, operating conditions, and food matrices (Figure 4). In addition, some studies evaluated more than one technology; therefore, the categories were not mutually exclusive. Differences in treatment intensity, processing time, temperature, moisture, sample physical state, and treatment combinations limit direct comparisons among studies. Consequently, the frequency with which a technology has been investigated should not be interpreted as a measure of effectiveness, and its effects should be analyzed in relation to the food matrix, target protein, and assessment method.

**Figure 5:**
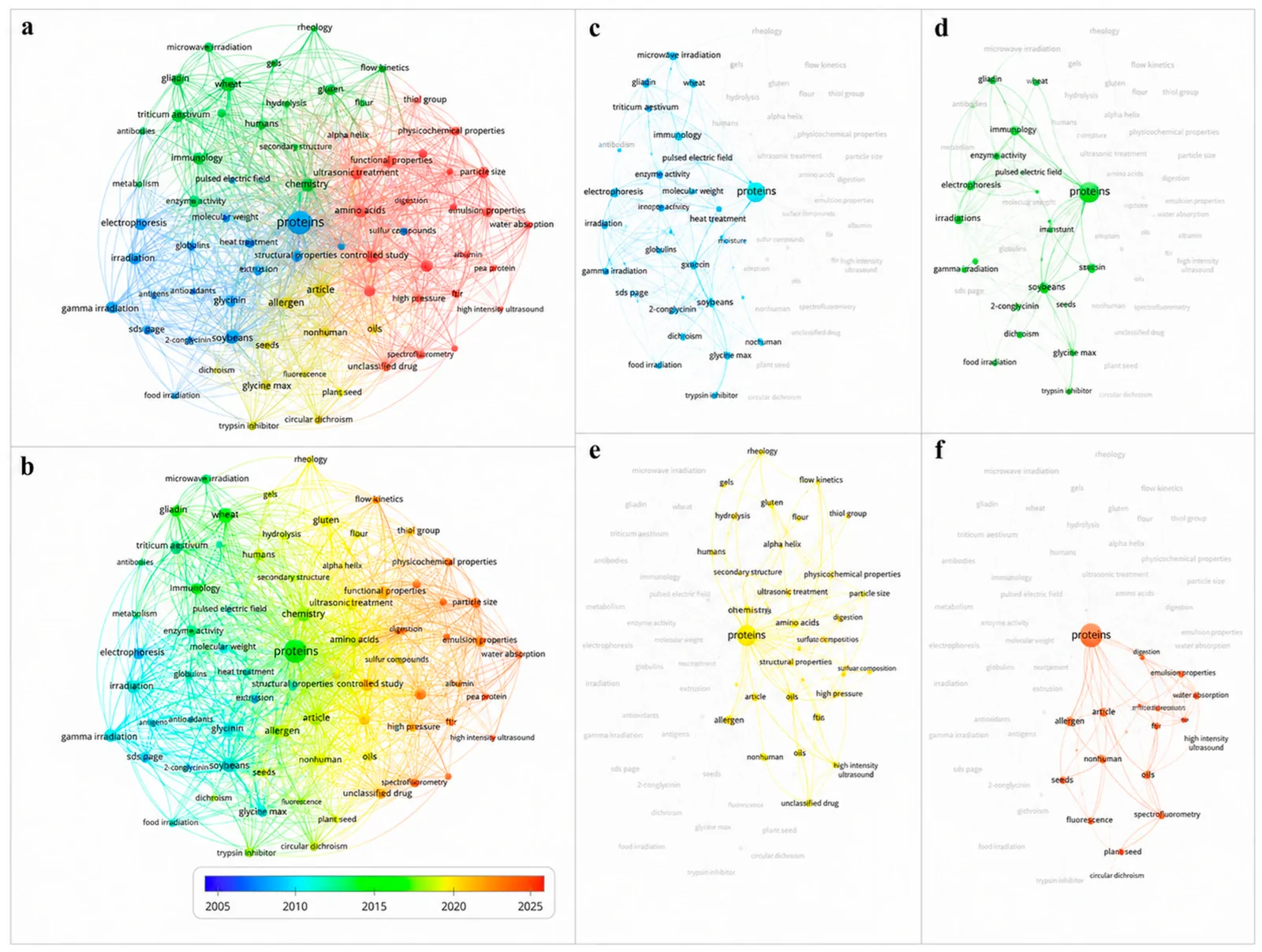
Keyword co-occurrence and temporal evolution of the included studies: (**a**) network visualization; (**b**) overlay visualization; and (**c**–**f**) temporal subnetworks showing the progression from earlier to more recent research topics. Node size represents occurrence frequency, links indicate co-occurrence strength, and colors represent thematic clusters or average publication year.

The keyword co-occurrence network revealed a highly interconnected research field organized around the central term proteins (Figure 5a). The network visualization identified several complementary thematic clusters. One cluster was mainly associated with gluten-containing cereals and included terms related to wheat, gliadin, gluten, rheology, and immunological responses. A second cluster focused on legume proteins, particularly soybean storage proteins such as glycinin and β-conglycinin, together with analytical approaches including SDS-PAGE and electrophoresis and treatments based on gamma or food irradiation. A third cluster comprised terms related to processing-induced structural and functional changes, including secondary structure, particle size, digestion, water absorption, and emulsifying properties. The close connections among these clusters indicate that the field increasingly integrates food matrix composition, processing technology, protein structure, and immunological assessment rather than examining these elements independently.

The temporal overlay and the four period-specific subnetworks illustrate the progressive evolution of the research focus (Figure 5b–f). Earlier studies were primarily associated with irradiation, electrophoretic characterization, and the detection of major soybean proteins (Figure 5c). The intermediate period showed greater attention to wheat, gliadin, immunology, enzyme activity, and trypsin inhibitors, reflecting a transition toward the biological relevance of individual protein fractions (Figure 5d). Subsequent research increasingly incorporated secondary structure, hydrolysis, ultrasound treatment, and functional properties, indicating a shift toward mechanistic evaluation of processing-induced protein modifications (Figure 5e). The most recent network emphasized pea protein, high-pressure processing, high-intensity ultrasound, digestion, particle size, water absorption, and emulsion properties (Figure 5f). Overall, this progression suggests that the field has moved from descriptive protein detection toward a more integrated understanding of how emerging technologies affect protein conformation, digestibility, functionality, and potential immunoreactivity.

### 3.2. Allergenic and Immunoreactive Proteins Identified in Legumes and Gluten-Containing Cereals

As summarized in Table 1, the evidence was unevenly distributed across the food matrices and protein families evaluated. Legumes comprised a relatively diverse range of species, although research was clearly concentrated on soybean [23,32,33,34,35,36,37,38,39]. Among cereals, wheat was the predominant matrix [40,41,42,43,44,45,46,47,48,49,50,51]. In both groups, storage proteins, particularly legume globulins and cereal prolamin and glutenins, were the main research targets because of their abundance, technological relevance, and potential involvement in adverse immune responses [52,53,54,55,56,57,58].

In soybean, the most extensively investigated proteins were 7S β-conglycinin (Gly m 5) and 11S glycinin (Gly m 6), with reported contents ranging from 0.16 to 28.99 mg·mL^−1^ depending on the food matrix, extraction procedure, and analytical method [32,33,34,60,61,62,63,64]. Their oligomeric organization, hydrophobic interactions, and disulfide bonds contribute to structural stability and may influence epitope accessibility and susceptibility to proteolysis. However, a substantial proportion of the studies primarily evaluated conformational, digestive, or techno-functional changes. Therefore, modifications in protein structure, solubility, or digestibility should not be independently interpreted as evidence of reduced allergenicity.

Kunitz-type protease inhibitors and lectins were also relevant targets because of their combined allergenic and antinutritional significance. Residual trypsin inhibitor activity reached values of up to 522.40 TIU·mg^−1^ in some samples [35]. Nevertheless, this activity primarily represents an antinutritional indicator rather than a direct measurement of immunoreactivity. By reducing digestive protease activity, these inhibitors may indirectly prolong gastrointestinal exposure to intact proteins, but this effect does not independently demonstrate increased clinical allergenicity [35]. For other legumes, including pea, peanut, and lupin, the available information was frequently based on electrophoretic profiles, identification of protein fractions, and evaluation of structural or functional properties [52,53,54,55,56,57,58,84,85]. Direct immunological evidence was more consistent for the major peanut allergens and selected mung bean proteins, whereas lentil and guamuchil were mainly investigated through compositional and techno-functional approaches [94,95].

Among cereals, research focused primarily on gliadins, including the α/β, γ, and ω fractions, and high- and low-molecular-weight glutenin subunits. Reported contents ranged from 1.29 to 2.21% for gliadins and from 2.52 to 3.36% for glutenins [15,40,96]. Although these proteins are highly relevant from an immunological perspective, their effects must be interpreted according to the biological mechanism evaluated. Some wheat proteins participate in IgE-mediated allergic reactions, whereas specific gluten-derived peptides are involved in the immune response associated with celiac disease. Accordingly, wheat allergy and gluten-related immunogenicity should not be considered equivalent outcomes [15,24,96,97,98].

Compared with gluten proteins, wheat albumins, globulins, and wheat germ agglutinin were less frequently investigated and generally lacked comparable quantitative information. Oat was also underrepresented; the included studies primarily addressed structural and techno-functional modifications of avenalin and other oat-protein fractions rather than direct immunoreactivity [103,104]. Evidence concerning barley and rye was similarly insufficient to support consistent comparisons among food matrices and processing technologies.

Quantitative data were concentrated mainly on soybean and wheat, whereas evidence for other matrices was frequently limited to protein extractability, densitometric measurements, electrophoretic band intensity, enzymatic activity, or qualitative immunological detection. Moreover, reported contents and activities were expressed using different sample types, extraction procedures, analytical methods, and calculation bases. This heterogeneity limits direct comparisons, prevents the quantitative ranking of allergenic potency, and restricts the development of a robust meta-analysis of the effectiveness of emerging technologies. The main research gaps concern less-studied matrices, minor protein fractions, and the limited number of studies integrating structural, digestive, and immunological evidence.

### 3.3. Analytical Methods and Biological Models Used to Assess Allergenic and Immunoreactive Proteins

The interpretation of processing-induced effects on allergenic and immunoreactive proteins was strongly influenced by the assessment method. As summarized in Table 2, in vitro assays predominated, particularly immunochemical methods based on the recognition of proteins or epitopes by specific antibodies. Structural, proteomic, digestive, and functional techniques provided complementary evidence, whereas validation using biological models was uncommon.

ELISA, including sandwich, indirect, and competitive formats, was the most frequently used method for evaluating the effects of emerging technologies on cereals and legumes [25,26,61,62,96,97,106]. However, the biological meaning of the results depended on the antibody used. Assays based on IgE from sensitized individuals provided direct evidence of IgE-binding capacity, although they did not independently reproduce an in vivo allergic reaction or predict its clinical severity [15,66]. In contrast, assays based on IgG, animal antisera, or commercial monoclonal antibodies primarily reflected antigenicity or analytical detectability [23,59]. In wheat, R5 and G12 antibodies were used to detect the persistence of gluten and related epitopes after processing [15,26,96], whereas legume studies used a broader range of antibodies to evaluate proteins such as Gly m 5, Gly m 6, and major peanut allergens [23,59,62,90].

Western blotting and dot blotting complemented ELISA by identifying proteins or fractions that retained antibody binding [80,89,90,96,101,105]. Nevertheless, a reduction in immunological signal may result from degradation, aggregation, reduced extractability, or altered epitope accessibility. Therefore, signal loss in these assays should not automatically be interpreted as a proportional reduction in clinical allergenicity [80,89,90].

Structural and proteomic methods, including SDS-PAGE, chromatographic techniques, LC-MS/MS, and conformational spectroscopy, helped relate changes in immunoreactivity to modifications in protein integrity, aggregation, conformation, and peptide profiles [15,75,87,107]. These findings represented indirect evidence and did not independently demonstrate reduced IgE binding or allergenic potential. Similarly, simulated gastrointestinal digestion was used to assess the persistence of proteins and peptides under controlled digestive conditions [22,66,108], but the disappearance of an intact protein did not exclude the formation of immunoreactive fragments. Trypsin inhibitor activity assays primarily assessed the residual functionality of antinutritional factors rather than direct immunoreactivity [35,38].

Biological validation was limited. Sensitized murine models enabled the assessment of systemic immune responses, although extrapolation to humans was restricted by physiological and immunological differences [31]. Antisera obtained from cattle exposed to soybean-containing diets combined an in vivo exposure phase with subsequent ex vivo assessment of antibody responses [81]. Although these models incorporated greater biological complexity than exclusively analytical methods, species specificity and experimental complexity limited their application.

No substantial differences were observed between cereals and legumes in the predominance of immunochemical methods [26,59]. Cereal studies showed greater standardization using R5- and G12-based assays for gluten detection [15,96], whereas legume investigations combined a broader range of immunological, structural, and proteomic methods because of the diversity of their storage proteins [62,75]. The predominance of in vitro findings requires cautious interpretation of claims concerning “reduced allergenicity.” A more robust assessment should integrate human IgE binding, structural characterization, gastrointestinal digestion, and appropriate cellular or biological models to distinguish reduced detectability from epitope modification and an actual reduction in allergenic potential.

### 3.4. Effects of Emerging Processing Technologies on Allergenic and Immunoreactive Proteins and Associated Structural and Techno-Functional Changes

The effects of emerging processing technologies on allergenic and immunoreactive proteins varied according to the food matrix, protein fraction, treatment intensity, hydration level, and analytical method. Importantly, the immunological assays used across the included studies addressed different biological or analytical endpoints and were therefore interpreted separately. Electrophoretic, spectroscopic, proteomic, digestive, and techno functional measurements were treated as complementary mechanistic evidence. Accordingly, reduced analytical detectability, disappearance of a protein band, loss of antibody recognition in a non-IgE assay, or structural modification was not independently interpreted as confirmation of hypoallergenicity or reduced clinical immunogenicity.

#### 3.4.1. Ultrasound

Ultrasound modifies proteins mainly through acoustic cavitation, which generates microstreaming, shear forces, shock waves, and, under certain conditions, reactive species. These effects may induce protein disaggregation, unfolding, partial fragmentation, oxidation, or reaggregation. Such modifications may reduce, preserve, or even increase epitope accessibility; therefore, the immunological response depends on the protein, food matrix, and treatment severity [69,72,79].

As summarized in Table 3, soybean and wheat were the most extensively investigated matrices, although mung bean, pea, lentil, chickpea, lupin, guamuchil, and pea–oat blends were also evaluated [59,67,88,94,95,104,109,110]. Sonication conditions were highly heterogeneous, with powers generally ranging from 100 to 750 W, frequencies commonly near 20–40 kHz, treatment times ranging from a few seconds to 60 min, and different temperature-control systems. Some wheat studies used substantially higher frequencies or combined ultrasound with fermentation, cooking, transglutaminase, pH-shifting, or ultrasound-assisted freezing [50,59,88,96,111]. This methodological diversity limits direct comparisons and prevents identification of a processing window applicable to all matrices.

Direct immunological evidence showed that ultrasound applied alone generally produced partial reductions. In soybean slurry, 60 min of sonication reduced the immunoreactivity signal by approximately 50%, whereas a reduction greater than 99% was achieved only when ultrasound was combined with cooking [59]. In soybean sprouts, an approximately 51% decrease in reactivity assessed using human serum was reported, together with reduced trypsin inhibitor and lipoxygenase activities [39]. In soybean protein isolate, the reduction in IgE binding was more moderate, reaching 18.9% [60]. For isolated wheat gliadin, the maximum decrease in antigenicity was 33.03%, indicating that antibody-reactive fractions persisted even under high-power conditions [96].

Findings obtained through non-immunological methods require separate interpretation. In mung bean, combined ultrasound and pH-shifting reduced the abundance of Vig r 4 and β-conglycinin-like proteins; however, this conclusion was based on SDS-PAGE, LC-MS/MS, and in silico prediction without direct assessment of IgE binding [88]. In wheat, ultrasound combined with lactic acid fermentation or transglutaminase markedly reduced gliadin content or band intensity [50,111]. These outcomes indicate protein degradation or reduced detectability but do not necessarily demonstrate an equivalent decrease in immunoreactivity. Similarly, studies using ultrasound to enhance soybean-protein extraction reflected changes in analytical recovery and detectability rather than reduced allergenic potential of the food matrix [33,34,61].

Structural modifications depended on the protein fraction and treatment intensity. Decreased α-helix content, increased β-sheet or disordered structures, changes in sulfhydryl groups and disulfide bonds, particle-size reduction, and exposure of hydrophobic regions were reported [69,72,79,88,95,104,109,110]. These responses were not uniform: some proteins underwent unfolding and fragmentation, whereas others showed oxidation, aggregation, or rearrangement into more ordered structures. Changes in solubility, digestibility, emulsification, foaming, and gelation confirmed the reorganization of protein–water and protein–protein interactions but did not constitute direct evidence of reduced allergenicity.

The available evidence indicates that ultrasound effectively modifies the structure and techno-functional properties of plant proteins, but its independent effect on immunoreactivity was variable and generally incomplete. The largest reductions were obtained using combined treatments and therefore cannot be attributed exclusively to sonication. Most studies also lacked validation using human IgE, cellular models, or biological testing. Ultrasound should consequently be considered primarily a facilitating technology that may increase the susceptibility of certain proteins to subsequent thermal, fermentative, or enzymatic treatments, rather than a stand-alone strategy capable of producing clinically hypoallergenic foods.

#### 3.4.2. Irradiation

The irradiation technologies evaluated included ultraviolet (UV), gamma, and electron-beam irradiation. Gamma and electron-beam irradiation are ionizing treatments that can modify proteins through direct ionization of polypeptides and, particularly in hydrated systems, through water radiolysis. The latter generates reactive species, including hydroxyl radicals (•OH), hydrogen atoms (H•), and hydrated electrons (e^−^_aq), which may induce oxidation, bond cleavage, unfolding, fragmentation, and protein aggregation [35,36]. In contrast, UV irradiation is non-ionizing and acts through photon absorption by chromophores and sensitive amino acid residues, thereby initiating photochemical and photooxidative reactions [26]. Because these processes may destroy selected epitopes but may also expose previously buried regions or promote aggregation, irradiation does not necessarily produce a uniform reduction in immunoreactivity.

The evidence focused predominantly on soybean and wheat, with additional studies involving peanut, common bean, cowpea, chickpea, and black gram (Table 4). Experimental conditions were highly heterogeneous, ranging from low-dose gamma irradiation of seeds to treatments of 200 kGy, together with different UV exposure times and electron-beam doses. This variability limits direct comparisons and emphasizes that absorbed dose should be interpreted together with matrix hydration, physical state, complementary treatments, and the analytical method used.

In wheat, UV irradiation decreased the G12-reactive signal by 58–64%, whereas combined UV–infrared treatment and UV exposure after baking produced more variable responses [26]. The reduction coincided with modifications of gliadins, –SH/S–S oxidation, and weakening of the gluten network [26,113]. However, decreased G12 recognition should be interpreted as reduced analytical detection of selected gluten-related motifs rather than evidence of lower IgE-mediated allergenicity or safety for individuals with celiac disease. Previous baking also attenuated the response to subsequent UV exposure, highlighting the influence of matrix structural state on processing effectiveness.

Hydration was a major determinant of gamma-irradiation effects. In soybean trypsin inhibitor systems, 10 kGy decreased inhibitor activity by 80–85% in aqueous conditions, whereas dry powder required doses of up to 200 kGy to achieve smaller reductions [35,36]. Although inhibitor activity is an antinutritional rather than immunological outcome, these findings demonstrate the importance of water-radiolysis products in protein modification. Hydration dependence also represents a technological limitation because dry seeds and flours showed greater radioresistance, while intensive treatments were associated with aggregation and loss of solubility.

Direct immunological evidence in legumes was similarly matrix- and treatment-dependent. In the soybean cultivars Enrei and Fukuyutaka, IgE reactivity decreased at 20–30 kGy, although responses varied among sera and several major allergenic proteins remained detectable [36,71]. In kidney bean, black gram, and peanut, gamma irradiation alone at doses up to 25 kGy did not significantly alter IgE binding. In contrast, combining 25 kGy with boiling decreased IgE binding by 52.1–89.0%, depending on the matrix and protein fraction [25]. These reductions coincided with extensive aggregation and decreased solubility and therefore cannot be attributed exclusively to irradiation or interpreted without considering altered recovery of soluble and insoluble proteins.

Wheat showed particularly contrasting responses. Irradiation of wheat germ agglutinin decreased its antigenicity, intestinal-cell binding, and inflammatory responses in sensitized mice [31], providing biological evidence beyond structural measurements. However, wheat germ agglutinin is not a gluten protein, and these findings cannot be extrapolated to gliadin immunogenicity in celiac disease. Conversely, gamma irradiation of commercial gliadin increased immunoreactivity to 174.1% and IgE binding by up to approximately 500%, while increased antibody recognition was also observed in wheat flour [44,101]. Fragmentation of ω-gliadins, loss of native conformation, and aggregation may have increased the accessibility of previously less exposed regions. These results clearly demonstrate that fragmentation or greater structural disruption does not necessarily predict reduced immunoreactivity.

Several studies applied low-dose gamma irradiation directly to soybean or wheat seeds and reported subsequent changes in globulin abundance, gliadin and glutenin profiles, and trypsin inhibitor activity [40,65,77,100]. However, these experiments primarily assessed physiological or heritable responses following seed irradiation and generally lacked antibody-binding assays. They should therefore be regarded as indirect evidence of altered protein profiles rather than evidence of reduced immunoreactivity. Similarly, irradiation-induced changes in digestibility, solubility, or antinutritional activity are relevant to nutritional quality but cannot independently indicate lower allergenic potential.

Evidence for electron-beam irradiation was more limited and focused on soybean flour. Treatment modified β-conglycinin and glycinin subunits, promoted fragmentation, and altered the 11S/7S ratio, together with changes in solubility, trypsin inhibitor activity, and digestibility [32,108]. Because IgE or other allergen-specific antibody binding was not assessed, these findings represent indirect structural and functional evidence and cannot establish whether epitopes were destroyed, exposed, or masked.

The available evidence indicates that irradiation responses depend on irradiation modality, food matrix, hydration, absorbed dose, target protein, and assessment method. The most relevant evidence of immunological modification was provided by IgE-binding assays and biological models, whereas changes in SDS-PAGE profiles, protein structure, digestibility, solubility, or enzyme activity should be regarded as complementary evidence. Although UV and gamma irradiation decreased selected immunoreactive signals, techno-functional losses and increased antibody recognition were also observed in some wheat systems. Moreover, some of the largest decreases in IgE binding in legumes were obtained through combined treatments, limiting attribution to irradiation alone. Thus, current evidence supports system-dependent modulation of immunoreactivity but does not demonstrate that any irradiation modality alone produces clinically hypoallergenic foods or wheat products that are safe for individuals with celiac disease.

#### 3.4.3. Microwave

Microwave processing modifies food proteins primarily through dielectric heating, in which electromagnetic energy interacts with polar molecules and ionic species to generate rapid volumetric heating through dipole rotation and ionic conduction [61]. Consequently, most protein modifications arise from the thermal history generated within the matrix, although heating rate and spatial energy distribution may differ substantially from those of conventional heating. Depending on moisture content, treatment intensity, and protein environment, microwave exposure may promote unfolding, aggregation, fragmentation, sulfhydryl–disulfide interchange, and secondary- and tertiary-structure rearrangement. These changes may alter epitope accessibility, digestibility, and techno-functional behavior but do not necessarily reduce immunoreactivity.

The available evidence focused predominantly on soybean and wheat and encompassed diverse matrices, including flours, protein isolates, edamame milk, glycosylated protein systems, whole grain, isolated gliadin, hydrated or powdered gluten, and gluten hydrolysates (Table 5). Treatment conditions also varied considerably in power, exposure time, hydration, and final temperature. This heterogeneity is particularly relevant because microwave energy absorption strongly depends on matrix water content and dielectric properties. Nominal microwave power alone is therefore insufficient for comparing treatment severity across studies; moisture, sample geometry, final temperature, and time–temperature history should also be considered.

In soybean systems, the evidence was predominantly analytical, structural, digestive, or antinutritional rather than directly immunological. Microwave-assisted extraction decreased ELISA-detectable soybean protein recovery from 28.99 to 19.51 mg/mL in raw flour and from 2.39 to 0.88 mg·mL^−1^ in protein isolate [61]. Because this study evaluated allergen extraction and analytical recovery, these decreases should not be interpreted as equivalent reductions in allergenicity or IgE binding. In edamame milk, microwave heating decreased trypsin inhibitor activity by approximately 74% while increasing in vitro protein digestibility from 81.05% to 86.60% [38]. Although nutritionally relevant, neither reduced inhibitor activity nor improved digestibility independently demonstrates reduced immunoreactivity.

Structural responses in soybean further demonstrate that microwave-induced conformational changes are not unidirectional. In edamame proteins, α-helix content decreased from 15.41% to 6.84% after treatment [38], whereas glycosylated soybean 7S globulin showed an increase from 19.68% to 47.86% after 60 s of microwave exposure, accompanied by decreased β-sheet structures and increased particle size [74]. These opposite responses indicate that unfolding, rearrangement, and aggregation depend on initial protein conformation, molecular interactions, glycosylation, hydration, and treatment severity. Changes in water-holding capacity, emulsifying behavior, dispersibility, or solubility therefore indicate altered protein interactions rather than direct evidence of allergen mitigation.

In wheat, direct immunochemical evidence was more frequent but considerably less consistent. Microwave pretreatment followed by enzymatic hydrolysis decreased the R5-reactive gluten signal by approximately 60%, reaching 39.65% of the untreated control [106]. However, because enzymatic hydrolysis followed microwave pretreatment, this response cannot be attributed exclusively to microwave exposure. Furthermore, reduced R5 recognition indicates lower analytical detection of selected gluten sequences rather than reduced IgE-mediated wheat allergy or absence of celiac-related immunogenicity. In contrast, microwave treatment of wheat gluten powder increased the R5-reactive signal from 227.5 to 298.8 g·kg^−1^, approximately 31% [98], whereas medium-strength wheat flour showed no quantifiable reduction in IgG immunoreactivity [97]. Thus, microwave treatment may decrease, preserve, or increase antibody recognition depending on the protein system and processing conditions.

A particularly informative response was observed in commercial gliadin and wheat flour, where antibody recognition varied non-monotonically with microwave energy input [105]. Gliadin showed a pronounced increase in antibody-reactive signal at an intermediate energy input, reaching a reported value of approximately 210% at 40 kJ before approaching the untreated response at higher energy levels; wheat flour showed a similar pattern. Although the magnitude should be interpreted according to the normalization used in the original assay, the response demonstrates that increasing microwave energy does not necessarily produce a progressive decrease in immunoreactivity. Partial unfolding may expose previously buried antibody-binding regions, whereas more severe treatment may promote aggregation, degradation, or epitope masking. The concurrent loss of intact gliadin fractions and formation of high-molecular-weight aggregates further indicates that electrophoretic band disappearance or fragmentation cannot be used as surrogate evidence of allergen reduction.

Microwave-induced structural changes were also associated with technological trade-offs. In the microwave–enzymatic hydrolysis system, emulsifying activity increased from 11.63 to 50.66 m^2^·g^−1^ [106], indicating that extensive protein modification can improve selected interfacial properties even when the immunological contribution of each treatment cannot be separated. Conversely, prolonged microwave exposure of hydrated wheat gluten decreased protein solubility, while intensive treatment of whole-wheat grain impaired breadmaking-related properties [49,102]. These findings highlight the need to evaluate immunoreactivity together with solubility, rheology, nutritional quality, and final-product functionality.

The available evidence indicates that microwave processing can substantially modify legume and wheat proteins but does not support a consistent stand-alone reduction in immunoreactivity. In soybean, most studies describe changes in extraction behavior, antinutritional activity, digestibility, or protein conformation rather than direct reductions in IgE binding. In wheat, immunochemical responses range from substantial decreases under combined microwave–enzymatic treatment to unchanged or increased antibody recognition after microwave treatment alone. Structural disruption is therefore not synonymous with allergen mitigation, and the observed response depends on the balance among unfolding, fragmentation, aggregation, epitope exposure, and protein extractability. Future studies should include conventionally heated controls matched for time–temperature history, analyze soluble and insoluble allergenic protein fractions, and integrate human IgE-based assays with gastrointestinal digestion and residual-epitope characterization. Current evidence therefore supports microwave processing as a matrix- and condition-dependent protein-modification technology rather than an established stand-alone strategy for reducing food allergenicity.

#### 3.4.4. Cold Plasma

Cold plasma is a nonthermal processing technology based on the partial ionization of a gas through the application of an electric field, generating electrons, ions, photons, and reactive oxygen and nitrogen species (RONS). These species can react with susceptible amino acid residues and protein side chains, promoting oxidation, sulfhydryl-group modification, conformational rearrangement, fragmentation, and, depending on treatment severity, protein aggregation or cross-linking [75,76,80]. Because plasma action occurs predominantly at accessible regions of the sample, its effects can be strongly influenced by food structure, matrix composition, and protein exposure to reactive species. Consequently, although these modifications may alter epitope accessibility, protein oxidation or fragmentation alone cannot be considered evidence of reduced allergenicity.

The available studies ranged from purified glycinin and protein isolates to flours and intact foods, with soybean predominating and peanut, pea, grass pea, wheat, and oat receiving comparatively less attention (Table 6). Processing conditions were highly heterogeneous in plasma configuration, carrier gas, voltage, frequency, and exposure time. This variability limits the establishment of a universal relationship between treatment intensity and protein response, particularly because RONS composition and their interactions with proteins depend on both the plasma-generation system and matrix characteristics. Treatment severity should therefore not be compared solely based on voltage or exposure time.

The most consistent direct immunological evidence was obtained in soybean systems. In soy protein isolate, cold plasma reduced IgE binding by up to 75% after 5 min under the condition producing the greatest response; however, extending exposure to 10 min resulted in a subsequent increase in IgE binding [75]. This non-linear behavior indicates that greater treatment intensity does not necessarily lead to a progressive decline in immunoreactivity. An even more pronounced effect was observed in purified glycinin, where IgG and IgE binding decreased by 91.64% and 81.49%, respectively, after 5 min [80]. Simultaneously, α-helix content decreased from 25.77% to 11.06%, together with polypeptide fragmentation and oxidation of aromatic residues. These findings support an association between oxidative structural modification and reduced antibody recognition, although they do not imply that every conformational alteration will produce the same immunological response.

The biphasic response observed in soy protein isolate further suggests that the balance among unfolding, fragmentation, and aggregation may ultimately determine epitope accessibility. Moderate modification may destroy or mask selected antibody-recognized regions, whereas prolonged exposure may promote new molecular associations or expose regions that were previously less accessible [75]. This distinction is particularly relevant because another study on soy protein isolate demonstrated progressive oxidation, carbonyl groups increased from 2.39 to 4.36 μmol·g^−1^ and free –SH groups decreased from 4.49 to 3.25 μmol·g^−1^, together with secondary-structure rearrangement and improved gel properties, but without directly assessing immunoreactivity [76]. Thus, the extent of protein oxidation cannot independently be used as a predictor of the immunological response.

The role of matrix structure was especially evident in peanut. After 60 min of treatment, Ara h 1 antigenicity decreased by approximately 43–44% in defatted peanut flour but by only 9.3% in whole kernels exposed under comparable conditions [89]. Although these measurements evaluated antigenicity rather than human IgE binding, the pronounced difference between the two matrices suggests that physical protein accessibility is an important limitation to plasma efficacy. Tissue disruption and the lower lipid content of defatted flour probably facilitated contact between reactive species and Ara h 1, whereas cellular organization and the composition of the intact kernel may have exerted a protective effect. This finding cautions against directly extrapolating the high effectiveness observed in purified proteins or disrupted matrices to intact foods.

Studies in other legumes further support the ability of cold plasma to modify protein conformation and interfacial properties, although without directly demonstrating reduced immunoreactivity. In grass pea protein isolate, free –SH groups decreased by approximately 33.5–45.5%, together with increased carbonyl and dityrosine formation, globulin dissociation, and changes in secondary structure [55]. In pea protein concentrate, the carrier gas influenced the conformational response; under selected air-plasma conditions, α-helix content decreased from 17.47% to 15.82%, accompanied by an increase in disordered structures [54]. These modifications were associated with moderate improvements in solubility, emulsion stability, and water- or oil-holding properties. In the absence of antibody-binding assays, however, these effects should be regarded as complementary mechanistic evidence rather than proof of allergen attenuation.

A similar distinction applies to cereal proteins. In hard wheat flour, free –SH content decreased from 17.5 to 9.8 nmol·mg^−1^ protein, whereas disulfide-mediated cross-linking and protein polymerization increased, with the elastic modulus reaching 57.8 kPa [47]. Whole wheat kernels also exhibited changes in α-helix and β-sheet contents and in gluten-network microstructure, accompanied by modifications in wet-gluten content and dough stability [107]. These findings demonstrate substantial remodeling of gluten supramolecular organization, but neither study directly assessed specific IgE binding or celiac-related epitopes. Increased polymerization, reduced extractability, or modification of the gluten network should therefore not be interpreted as evidence of lower immunogenicity. Similarly, an oat protein–casein system subjected to combined cold-plasma and pH-shifting treatment showed a 14.49% increase in solubility and a reduction in emulsion-droplet size from 5.36 to 3.07 μm [103]. Because immunoreactivity was not assessed and two processing approaches were combined, these changes cannot be attributed exclusively to cold plasma or directly related to reduced allergenicity.

Cold-plasma-induced structural modifications can also result in substantial techno-functional changes. In purified glycinin, solubility increased from 10.78% to 65.96%, whereas the emulsifying activity index increased from 21.08 to 160.29 m^2^·g^−1^ [80]. Selected treatments of soy protein isolate increased solubility at pH 4.5 by up to 282%, together with substantial improvements in emulsifying and foaming properties [75]. These responses indicate that controlled oxidation and partial unfolding can enhance the exposure of hydrophilic or interfacial active regions. Nevertheless, the coexistence of improved functionality with non-linear immunological responses demonstrates that techno-functional performance and immunoreactivity do not necessarily change in parallel.

From technological and immunological perspectives, cold plasma shows considerable potential for modifying soybean proteins, with antibody-based assays reporting substantial reductions in recognition under specific treatment conditions. However, these effects remain sensitive to plasma intensity, exposure time, reactive-species composition, protein physical state, and the analytical procedure used. However, the response depends on treatment severity and, notably, on protein accessibility within the food matrix. The contrasting responses of defatted peanut flour and whole kernels illustrate how physical matrix complexity can substantially limit the effectiveness observed in purified systems. Furthermore, for several of the matrices investigated, the available evidence remains predominantly structural or techno-functional. Cold plasma should therefore currently be regarded as a promising strategy for modulating the immunoreactivity of selected proteins, rather than as an established stand-alone treatment capable of producing clinically hypoallergenic foods. Future studies incorporating IgE from sensitized individuals, simultaneous analysis of soluble and insoluble protein fractions, and evaluation of intact food matrices will be essential to determine whether plasma-induced oxidation destroys epitopes, temporarily masks them, or facilitates the exposure of new antibody-recognized regions.

#### 3.4.5. Extrusion Cooking

Extrusion is a continuous thermomechanical process, generally operated under high-temperature, short-time conditions, in which proteins are simultaneously subjected to heating, pressure, shear, and intensive mixing. These conditions can disrupt the non-covalent interactions stabilizing native protein conformations, promote polypeptide unfolding, and subsequently favor aggregation, sulfhydryl–disulfide interchange, and intermolecular association [37,81,85]. The extent of these transformations depends on temperature, moisture content, screw speed, specific mechanical energy (SME), residence time, and matrix composition. At lower moisture contents, higher melt viscosity may favor mechanical-energy transfer and extensive protein association, whereas during high-moisture extrusion, water acts as a plasticizer, increasing protein mobility and alignment during the formation of fibrous structures. These transformations may destroy, mask, or expose antibody-recognized regions; therefore, greater denaturation or reduced protein extractability does not necessarily imply lower immunoreactivity.

The included studies focused predominantly on soybean and pea, with additional evidence from common bean and wheat (Table 7). Matrices ranged from flours and ground plant fractions to protein isolates and blends designed for high-moisture texturization. This diversity strongly influences structural responses because starch, fiber, and other matrix components alter the transfer of heat and mechanical stress to the protein phase. Low- and high-moisture extrusion also generate markedly different rheological environments and, consequently, different patterns of unfolding, aggregation, and molecular alignment. Nominal process severity therefore cannot by itself predict changes in protein immunoreactivity.

The most consistent evidence of decreased antigenicity was obtained in soybean. In ground soybean hypocotyl, extrusion reduced overall antigenicity by up to approximately 99%, together with loss of detectable α, α′, and β subunits of β-conglycinin and the A3 subunit of glycinin [81]. Susceptibility differed substantially among protein fractions, however: A1A2A4 retained approximately 22.3% of control antigenicity, while an approximately 33–34-kDa allergenic protein retained about 66% of its initial reactivity [81]. Soybean flour showed an even greater reported decrease, approaching 99.9%, with approximately 0.1% residual antibody recognition [37]. These findings demonstrate that extrusion can markedly reduce antibody recognition in soybean, while also showing that loss of selected subunits does not necessarily indicate complete elimination of all immunoreactive proteins.

In pea, the relationship among thermomechanical severity, structural rearrangement, and antibody recognition is particularly relevant. Under conditions exceeding 250 kWh/t SME, anti-legumin antibody reactivity decreased [85]. This response was associated with greater protein unfolding and cross-linking, together with shear-induced alignment, suggesting that rearrangement of the 11S fraction can modify the accessibility of antibody-recognized regions. Nevertheless, high SME should not be assumed to universally destroy conformational epitopes, because the response remains dependent on both protein architecture and processing conditions.

Studies without direct immunological assessment provide important mechanistic information. During low-moisture extrusion of pea protein isolate, α-helical and β-sheet structures decreased, whereas β-turn content increased from 9.7% to 13.3%; aggregation or disappearance of the approximately 60-kDa legumin monomer was also observed [52]. Under high-moisture extrusion, detectable legumin bands decreased while vicilin and convicilin remained visible by SDS-PAGE [57]. At the same time, inter- and intramolecular disulfide-mediated interactions increased and urea-soluble protein decreased from approximately 100% to 60%, without evidence of new peptide-bond formation [57]. This pattern is consistent with extensive protein association mediated primarily by covalent and non-covalent interactions. Persistence of vicilin or convicilin bands, however, only indicates electrophoretic detectability and does not demonstrate preservation of native conformation or immunological activity.

Extensive molecular rearrangement was likewise observed in pea cv. Ballet and Pinto kidney bean. In pea, free –SH groups decreased from 21.7 to 5.5 μmol·g^−1^, while disulfide-linked insoluble protein increased from 25.5 to 157.9 g·kg^−1^ [84]. These changes coincided with a decrease in soluble protein from 760 to 220 g·kg^−1^ and an increase in water-holding capacity from 1.24 to 2.86 g·g^−1^. In Pinto kidney bean, soluble protein decreased from 730 to 140 g·kg^−1^ and approximately 14–22-kDa fragments appeared [84]. These findings indicate that extrusion may simultaneously promote fragmentation, unfolding, and extensive disulfide-mediated aggregation. In the absence of antibody-binding assays, however, such responses should be interpreted as structural and functional evidence rather than proof of reduced allergenicity.

High-moisture extrusion also produced relevant nutritional changes. In a pea protein–sunflower meal blend, trypsin inhibitor activity decreased by 64% and phytic acid by 83%, while in vitro protein digestibility reached approximately 86–89% [114]. Anisotropic fibrous structures were simultaneously formed, illustrating the ability of the process to reorganize proteins into oriented networks. These responses are relevant for nutritional quality and applications such as meat analogs, but decreases in antinutritional factors and increased digestibility do not independently demonstrate lower IgE binding.

Wheat exhibited a markedly different response from soybean. Extrusion of white wheat flour produced only an approximately 2% decrease in R5/G12-reactive gluten signal, and this difference was not statistically significant [15]. The immunochemical response should therefore be interpreted as essentially unchanged. This occurred despite pronounced structural remodeling: α-helix content decreased from 32.1% to 16.6%, intermolecular β-sheet structures increased from 26.2% to 32.8%, and soluble protein decreased from 6.5% to 2.3% [15]. The coexistence of extensive structural disruption with an essentially unchanged R5/G12 signal provides a particularly clear example that denaturation, aggregation, and reduced extractability do not necessarily decrease recognition of relevant gluten sequences. Moreover, R5 and G12 detect selected gluten motifs and cannot independently establish safety for individuals with celiac disease.

Other wheat extrusion treatments confirmed extensive matrix restructuring without providing direct immunological evidence. In baking-enzyme-enriched wheat flour from cultivar *Laudias*, extrusion promoted gluten-protein denaturation, compaction of the starch–protein matrix, reduced crystallinity, and formation of amorphous starch–lipid phases [115]. These changes increased sodium-carbonate solvent retention but were accompanied by loss of coherent dough-forming capacity. Extrusion-induced structural modifications may therefore produce substantial technological consequences and, when they markedly reduce protein solubility or extractability, may also influence the amount of protein recovered during immunological assays. This reinforces the need to evaluate soluble and insoluble fractions simultaneously.

The response to extrusion is therefore strongly dependent on the food matrix, target protein, and thermomechanical conditions. Soybean provides the strongest immunological evidence, with antigenicity reductions approaching 99–99.9%, although selected fractions retain residual recognition [37,81]. In pea, reduced anti-legumin reactivity under high-SME conditions occurs alongside substantial unfolding, cross-linking, and protein alignment [85], while much of the remaining evidence is structural. Wheat provides a contrasting example in which extensive secondary-structure and solubility changes were not accompanied by a significant decrease in R5/G12 recognition [15]. Extrusion should therefore be regarded as a technology capable of modulating immunoreactive proteins in selected matrices, but thermomechanical severity cannot be considered a universal predictor of allergen reduction. Future studies should integrate IgE from sensitized individuals, analysis of soluble and insoluble protein fractions, and systematic designs linking moisture, SME, and temperature to epitope persistence following gastrointestinal digestion.

#### 3.4.6. Pressure-Based Technologies

Pressure-based technologies comprise processes with partially distinct mechanisms. High-pressure processing (HPP) exposes foods to elevated pressures transmitted uniformly through a liquid medium, favoring volume-dependent changes and modification of non-covalent interactions, including hydrogen bonding and electrostatic and hydrophobic interactions, while primary peptide bonds remain comparatively stable [23,66,82]. In contrast, high-pressure homogenization (HPH), ultra-high-pressure homogenization (UHPH), and other dynamic pressure-based technologies additionally involve intense shear, turbulence, and cavitation, which promote aggregate disruption and protein structural rearrangement [11,56,60]. These mechanistic differences are relevant because pressure-based technologies do not necessarily generate the same molecular modifications or, consequently, the same immunological responses.

The included studies focused predominantly on soybean and pea systems, with additional evidence from wheat (Table 8). Processing conditions varied considerably in applied pressure, number of passes, holding time, moisture, and temperature. Temperature deserves particular attention in dynamic systems because final temperatures of up to 86 °C were reached during UHPH [11]. Protein modifications in these systems may therefore result from the combined effects of pressure, shear, and heating, complicating attribution to pressure alone.

Whole soymilk displayed a clearly non-monotonic immunological response to HPP. At 300 MPa for 5 min, anti-soybean trypsin inhibitor antibody recognition decreased to approximately 44.39% of the control, whereas at 600 MPa it increased to approximately 109% [23]. The more intensive treatment was accompanied by a marked decrease in α-helical structure, increased β-sheet organization, and greater aggregation. Thus, greater pressure-induced structural disruption did not produce a progressive decrease in antibody recognition, suggesting that unfolding and aggregation may also alter the exposure or analytical recovery of reactive regions.

A direct comparison of dynamic and static pressure treatments in soy protein isolate showed that HPH at 90 MPa decreased antibody reactivity by approximately 29.8%, whereas HPP at 350 MPa produced a 46.6% reduction [60]. Both treatments modified protein conformation and increased exposure of previously buried regions, although through different structural rearrangements. However, the absence of validation using sera from sensitized individuals limits interpretation of the clinical relevance of these reductions.

A substantially greater decrease was reported for β-conglycinin (Gly m 5), with antibody-reactive protein declining by ≥99.5% at 400–500 MPa [66]. Importantly, this response occurred during HPP-assisted enzymatic hydrolysis, rather than pressure treatment alone. HPP promoted unfolding and exposure of protease-accessible regions, facilitating subsequent fragmentation of β-conglycinin subunits. The response should therefore be attributed to the combined intervention and suggests that pressure may function as a pretreatment that increases the susceptibility of immunoreactive proteins to enzymatic hydrolysis.

Studies without direct immunological assessment further demonstrated strong protein- and matrix-dependent responses. Increasing pressure in soybean glycinin produced effects ranging from subunit dissociation and partial unfolding to extensive aggregation, whereas pea lectin largely retained its structural organization after high-pressure treatments [11,82,86]. In pea protein systems, HPH and dynamic high-pressure fluidization decreased aggregate size and modified molecular exposure and interfacial properties [56,58]. However, some of these studies involved combined treatments and none directly established changes in antibody binding; these findings should therefore be considered primarily mechanistic evidence rather than demonstration of reduced immunoreactivity.

In wheat, the response to HPP was strongly influenced by hydration. At 33% moisture, pressures ≥ 400 MPa promoted gluten aggregation and increased the unextractable polymeric protein fraction, whereas at 56% moisture and ≥500 MPa the gluten network became more discontinuous and structurally disrupted [48]. These findings demonstrate that water availability modifies the balance between aggregation and disruption of the protein network. However, IgE binding, R5/G12 reactivity, and other gluten-specific immunological markers were not assessed; consequently, these structural modifications cannot be interpreted as evidence of reduced wheat allergenicity or celiac-related immunogenicity.

The available evidence indicates that pressure-based technologies can induce responses ranging from moderate conformational rearrangement to dissociation, unfolding, fragmentation, and aggregation, but increasing treatment intensity is not linearly associated with reduced immunoreactivity. The biphasic response in whole soymilk [23], the moderate reductions obtained with HPH and HPP [60], and the marked decrease in Gly m 5 achieved only during HPP-assisted enzymatic hydrolysis [66] represent distinct mechanisms and levels of evidence. Findings in pea and wheat remain predominantly structural or techno-functional. Future studies should incorporate human IgE-based assays, analysis of soluble and insoluble fractions, and controls capable of separating mechanical, thermal, and combined-treatment contributions.

#### 3.4.7. Pulsed Electric Fields

Pulsed electric fields (PEF) are a processing technology based on the application of short-duration, high-intensity electric pulses to food matrices. Exposure to an electric field can modify charge distribution and the non-covalent interactions that stabilize proteins, including hydrogen bonds and electrostatic interactions, thereby promoting secondary- and tertiary-structure rearrangement, molecular unfolding, and exposure of previously less accessible groups [62,64]. These conformational changes may alter the accessibility of antibody-recognized regions and protein susceptibility to proteolysis. However, depending on the extent and nature of the structural rearrangement, epitopes may be destroyed, masked, or exposed; therefore, PEF-induced structural changes should not automatically be interpreted as reduced immunoreactivity.

The available evidence remains limited and primarily comprises soybean, pea, and wheat matrices (Table 9). Studies evaluated instant soy milk powder, soy protein isolate, pea protein extract, and vital wheat gluten using electric-field strengths of approximately 2.5–50 kV·cm^−1^ and markedly different pulse frequencies, pulse widths, treatment times, and temperatures [53,62,64,116]. This heterogeneity limits the establishment of a general relationship between field strength and protein response. Moreover, although PEF is generally considered a process with limited heating, temperatures of up to 58 °C were reported during treatment of soy protein isolate [64]. In the absence of thermally matched controls, a contribution of processing-induced heating to the observed structural modifications cannot therefore be excluded.

The only direct immunological evidence among the included studies was obtained in a soybean system. In instant soy milk powder, increasing the electric-field strength to 40 kV·cm^−1^ decreased the IgE-binding signal from 1.64 to 1.33, corresponding to an approximately 19% reduction [62]. IgG1 binding also decreased from 2.26 to a minimum of 1.61, although the value at 40 kV·cm^−1^ was 1.71, indicating a non-linear response. These changes were accompanied by tertiary- and secondary-structure rearrangement of β-conglycinin (Gly m 5) and glycinin (Gly m 6) and increased susceptibility to pepsin and pancreatin digestion [62]. The concurrence of structural modification increased digestive susceptibility, and decreased IgE binding suggests a plausible relationship among these responses. Nevertheless, the magnitude of the IgE reduction was moderate and derived from a single experimental system, preventing generalization of PEF as an allergen-reducing treatment.

In soy protein isolate, thermal analysis indicated apparent complete denaturation of the 7S and 11S fractions at 35 and 45 kV·cm^−1^, respectively, together with secondary-structure rearrangement and decreased denaturation enthalpy [64]. These findings suggest differential susceptibility of the major soybean globulins to electric-field treatment. However, antibody binding was not evaluated, and treatment temperature increased to 58 °C; therefore, the observed structural changes cannot establish either reduced immunoreactivity or an exclusively electric-field-mediated effect.

PEF also modified tertiary and secondary structures in pea protein extract. A red shift in intrinsic tryptophan fluorescence indicated protein unfolding, while parallel β-sheet content increased from 10.6% to 13.0% [53]. These molecular changes were accompanied by improvements in gelling capacity, solubility, and colloidal stability. However, because antibody-binding assays were not performed, the structural and functional responses cannot establish reduced allergenicity of pea proteins.

Pronounced structural rearrangement was likewise observed in vital wheat gluten. PEF decreased α-helical and β-turn structures while increasing parallel and antiparallel β-sheet organization, together with a 25.2% increase in free –SH groups [116]. These changes indicate extensive remodeling of the gluten protein network. However, the study did not assess IgE binding, R5/G12 reactivity, or other indicators of celiac-related immunogenicity. Consequently, extensive structural modification of gluten cannot independently demonstrate reduced persistence or recognition of immunologically relevant sequences.

The relationship between PEF-induced structural modification and immunoreactivity therefore remains insufficiently established. In soybean, PEF produced a moderate decrease in IgE binding together with structural rearrangement of Gly m 5/Gly m 6 and increased proteolytic susceptibility [62], providing the most direct connection among these responses. In contrast, evidence from soy protein isolate, pea, and wheat remains predominantly structural or techno-functional [53,64,116]. PEF cannot therefore be considered an established strategy for reducing food allergenicity. Future studies should incorporate sera from sensitized individuals, conventionally heated controls with matched time–temperature histories, analysis of soluble and insoluble protein fractions, and gastrointestinal digestion followed by residual-epitope characterization to determine whether PEF-induced conformational changes destroy, mask, or expose immunologically relevant regions.

#### 3.4.8. Other Processing Strategies and Combined Treatments

In addition to the technologies discussed above, the systematic search identified several other processing strategies represented by a limited number of studies, including pH shifting, enzymatic hydrolysis, pulsed light, ohmic heating, hydrodynamic cavitation, electrolysis, planetary ball milling, Maillard reaction, lactic fermentation, and combined treatments involving phenolic compounds (Table 10). These approaches operate through distinct mechanisms, ranging from changes in protein charge and non-covalent interactions to proteolysis, photooxidation, glycation, and mechanical forces. Because few studies were available for each strategy and combined treatments were frequently employed, the findings should be regarded mainly as preliminary evidence, and effects resulting from multi-step interventions should not be attributed to a single processing mechanism.

pH shifting produced marked changes in protein aggregation, molecular exposure, and functionality in legume systems, but without direct immunological evidence. In pea protein extract, alkaline treatment reduced aggregate size and increased free –SH groups and exposure of hydrophilic and hydrophobic regions, although primary and secondary protein profiles remained essentially unchanged [58]. In mung bean protein isolate, alkaline treatment decreased the abundance or analytical detectability of Vig r 4 and a β-conglycinin-like protein; when combined with ultrasound, particle size decreased from 5300 to 174.6 nm, and solubility increased from 45.9% to 91.2% [88]. However, SDS-PAGE, LC–MS/MS, and AllerCatPro 2.0 do not directly measure antibody binding, and effects obtained from the combined treatment cannot be attributed exclusively to pH shifting.

Enzymatic hydrolysis is mechanistically relevant because peptide-bond cleavage can modify regions containing linear epitopes. Nevertheless, both included studies evaluated hydrolysis after extrusion or thermal pretreatment and did not directly assess immunoreactivity. In soybean, the combined treatment achieved a degree of hydrolysis of 42.1%, decreased detectable intact glycinin and β-conglycinin subunits, and generated lower-molecular-weight peptides [70]. In vital wheat gluten, increasing hydrolysis similarly generated smaller protein fractions and improved solubility and emulsifying properties [41]. Disappearance of electrophoretic bands or production of smaller peptides, however, does not necessarily demonstrate elimination of immunoreactive epitopes, particularly because neither study assessed IgE binding, R5/G12 recognition, or other allergen-specific immunological markers.

Among the strategies grouped in this section, pulsed light provided the most notable direct immunological evidence. In hydrated vital wheat gluten, treatment decreased the R5-reactive gliadin signal by approximately 50%, together with partial depolymerization, disulfide-exchange reactions, and oxidation [24]. The smaller response observed in gluten powder highlights the importance of physical state and hydration. Nevertheless, decreased R5 recognition should be interpreted as reduced analytical detection of selected gluten motifs rather than evidence of safety for individuals with celiac disease. In whole peanut kernels, pulsed light decreased IgE-binding signals by up to approximately 80% for Ara h 1, Ara h 2, Ara h 3, and Ara h 4, together with fragmentation into polypeptides <17 kDa and formation of aggregates [90]. This study provides a more direct association between structural modification and reduced IgE recognition, although the evidence remains in vitro.

Other strategies also altered antibody recognition, although with greater interpretative limitations. Complexation of 7S β-conglycinin with punicalagin followed by ultrasound decreased IgG binding by 4.78–58.05%, depending on punicalagin concentration, but IgE was not evaluated [22]. Ohmic heating decreased Gly m TI immunoreactivity by approximately 36% in a soybean system and induced conformational and aggregation changes [68]. Because processing was conducted at 95 °C, however, thermal effects cannot be fully separated from the electrical contribution. These findings again illustrate that responses obtained from combined or thermally intensive processes cannot always be assigned to a single mechanism.

In contrast, hydrodynamic cavitation, electrolysis, planetary ball milling, Maillard reaction, and fermentation were supported primarily by structural, digestive, or techno-functional outcomes. Reported effects included changes in particle size, secondary structure, disulfide interactions, exposure of aromatic residues, aggregation, solubility, and interfacial properties [46,73,78,87,92]. Planetary ball milling produced substantial restructuring of wheat gluten, whereas Maillard treatment altered mung bean protein fractions and lupin fermentation modified the peptide profile generated after digestion. However, none of these studies directly assessed allergen-specific antibody binding; consequently, these changes cannot determine whether relevant epitopes were destroyed, masked, or remained immunologically accessible.

The evidence within this group therefore highlights a clear distinction between protein modification and demonstrated reduction in immunoreactivity. Pulsed light treatment of peanut currently provides the strongest direct evidence, whereas findings based on R5 recognition in wheat, IgG responses in soybean systems, or combined interventions require more restricted interpretation. For most other strategies, evidence remains structural, digestive, or functional. These approaches therefore represent promising routes for modifying allergenic and immunoreactive proteins, but the limited availability of human IgE-based assays and frequent use of combined treatments preclude general conclusions regarding allergenicity reduction. Future studies should directly link processing-induced structural changes with epitope persistence after gastrointestinal digestion and antibody recognition by sensitized individuals.

### 3.5. Comparative Assessment of Processing Technologies

**Figure 6:**
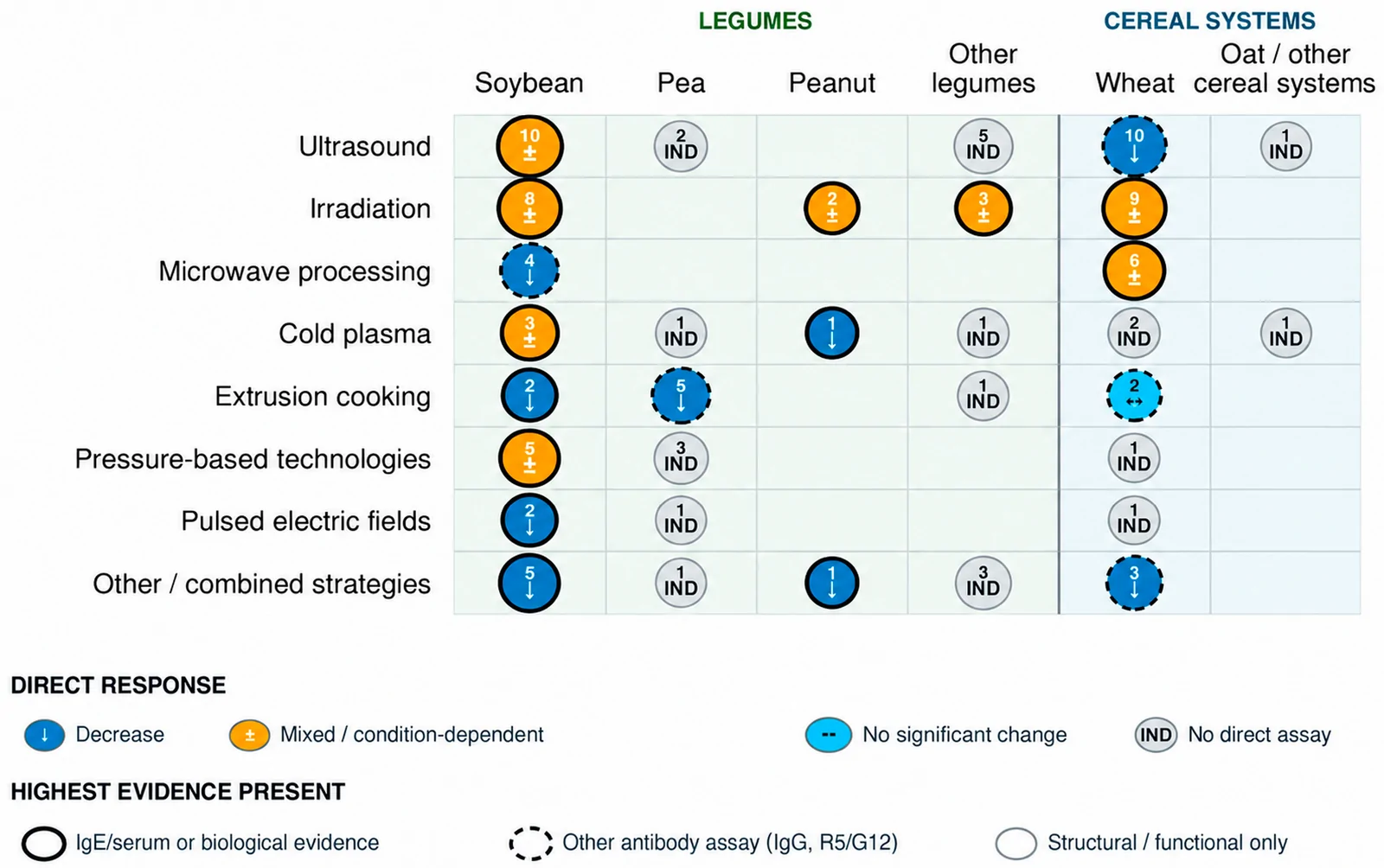
Evidence map of emerging processing technologies across legume and gluten-containing cereal matrices. Bubble area is proportional to the number of unique studies (n) contributing to each technology–matrix combination. Because some studies evaluated more than one technology or food matrix, the categories are not mutually exclusive. Fill color indicates the response pattern among studies using direct immunological assessments: decrease, mixed or condition-dependent response, increase, or no significant change. Gray bubbles (IND) indicate that only indirect structural, digestive, proteomic, or techno-functional evidence was available. Border style denotes the highest evidence category present within each cell: solid black, IgE/serum or biological evidence, including cellular, animal, or ex vivo assessments; dashed black, other antibody-based assays, including IgG and R5/G12; and gray, indirect evidence only. Data were derived from the 95 included studies summarized in Supplementary Tables S1 and S2.

Cross-technology comparison indicates that effectiveness of emerging processing technologies in reducing clinically relevant allergenicity or gluten-related immunogenicity cannot be established through a simple ranking of processing approaches. The included studies differed substantially in food matrix, target protein, treatment intensity, hydration state, extraction procedures, and analytical methods, and some investigations evaluated more than one technology simultaneously. Consequently, the frequency with which a technology has been investigated should not be considered a measure of effectiveness, and reported decreases in analytical or immunological readouts cannot be directly compared when they derive from different immunological assays, antibodies, or experimental conditions. Moreover, the biological relevance of these measurements differs substantially among IgE-binding assays, non-IgE antibody-based approaches, and R5/G12 gluten-reactivity assays. This heterogeneity also helps explain why technologies capable of inducing extensive structural modifications do not necessarily produce the greatest decreases in immunoreactivity. Accordingly, the technology–matrix evidence map shown in Figure 6 should be interpreted as an overview of evidence coverage, response patterns, and the strongest evidence category available, rather than as a ranking of technological effectiveness or clinical benefit.

At the matrix level, a general distinction emerged between legumes and gluten-containing cereals. Decreases in antibody-based analytical recognition were reported more consistently in legumes, particularly in soybean and peanut systems [83,88,97], although their magnitude depended on the target protein, processing conditions, and assessment method. These decreases should not be interpreted uniformly, because reductions in IgE binding using sera from sensitized individuals have a different biological significance from changes detected using IgG, animal antisera, or monoclonal antibodies. In gluten-containing cereals, especially wheat, responses were considerably more variable and included partial decreases, unchanged responses, and even increased immunological recognition [15,44,101]. When R5 or G12 antibodies were used, these responses reflected changes in the analytical recognition of selected gluten-related epitopes rather than direct evidence of reduced celiac-related immunogenicity. Irradiation provides a clear example of this variability: while selected treatments decreased immunoreactive signals, others markedly increased gliadin recognition, demonstrating that fragmentation or loss of native conformation may expose previously less accessible epitopes [44,101]. Findings obtained for a particular protein, antibody system, or food species should therefore not be directly extrapolated to other matrices or interpreted as evidence of reduced clinical risk.

**Figure 7:**
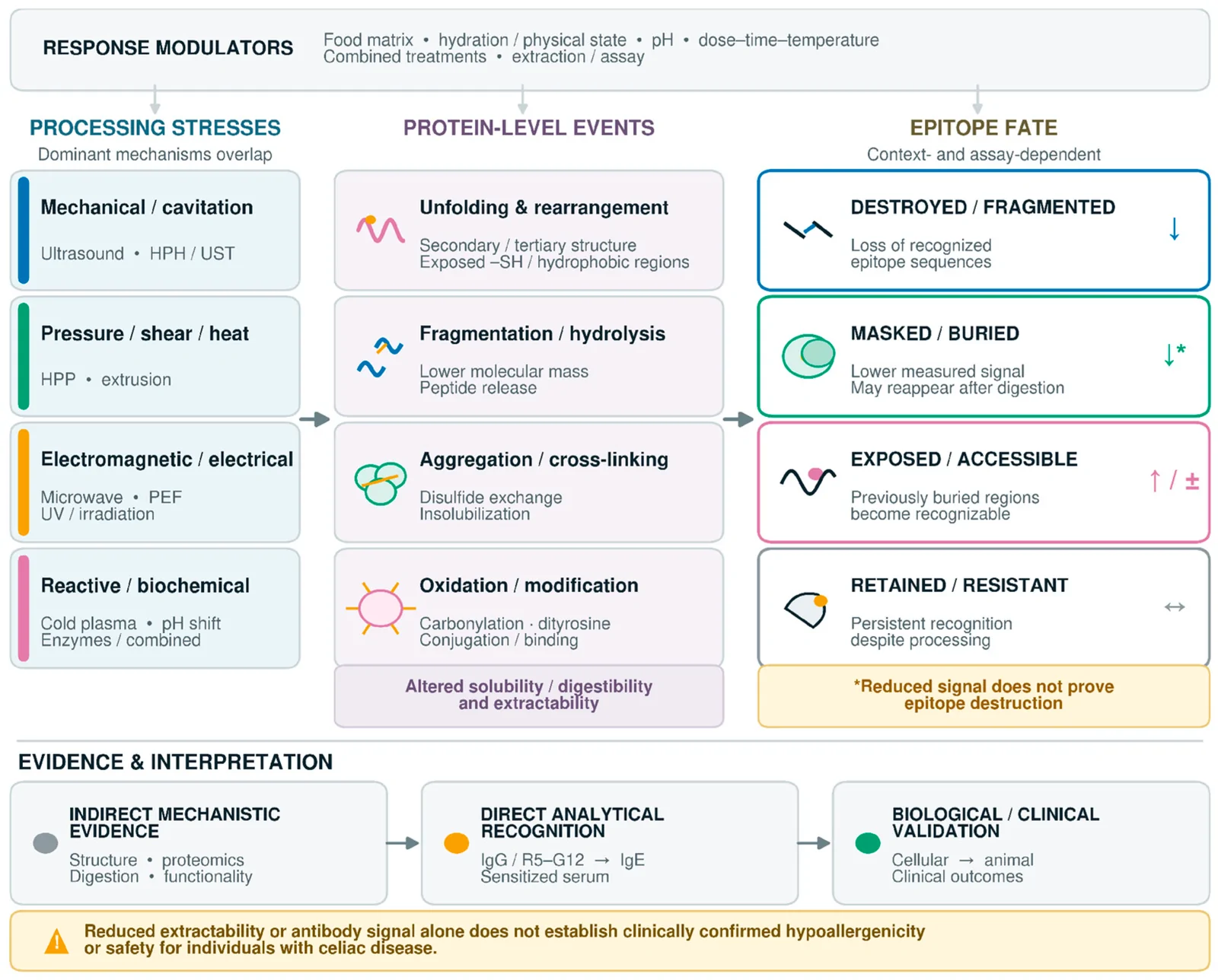
Integrated mechanism of processing-induced protein modification and epitope fate. Emerging processing technologies generate overlapping mechanical, thermal, electrical, oxidative, and biochemical stresses that may induce protein unfolding, fragmentation, aggregation, cross-linking, oxidation, and chemical modification. Depending on the food matrix, hydration state, processing intensity, complementary treatments, extraction procedure, and analytical method, epitopes may be destroyed, masked or buried, exposed, or retained. The lower pathway distinguishes indirect mechanistic evidence from direct antibody recognition and biological or clinical validation. Reduced extractability or antibody signal alone should not be interpreted as clinically confirmed hypoallergenicity or evidence of safety for individuals with celiac disease.

A common response across processing technologies involved shifts in the balance among protein unfolding, fragmentation, aggregation, and secondary- and tertiary-structure rearrangement. However, the extent of structural disruption was not monotonically related to decreased antibody recognition or analytical immunoreactivity [23,75]. Processing-induced structural changes may alter, mask, expose, or, in some cases, degrade antibody-recognized regions, depending on the protein, matrix, and treatment conditions [23,44,101]. This behavior was particularly evident for pressure-based treatments: in whole soymilk, antibody recognition decreased at 300 MPa but increased again at 600 MPa, despite more extensive secondary-structure rearrangement and aggregation at the higher pressure [23]. Accordingly, decreases in α-helical structure, increases in disordered regions, exposure of –SH groups, molecular-weight reduction, or disappearance of electrophoretic bands cannot independently be used as predictors of reduced IgE recognition. Antigenicity, gluten-related epitope detection, or clinical immunogenicity. The context-dependent pathways through which processing-induced protein modifications may alter epitope accessibility and immunological recognition are summarized in Figure 7.

Food-matrix characteristics and the use of combined treatments were additional determinants of processing response. Hydration, sample physical state, the presence of starch, lipids, and other matrix constituents, and partitioning of proteins between soluble and insoluble fractions may influence both processing susceptibility and analytical recovery [15,24,48,89]. In several cases, the greatest decreases in antibody-based analytical recognition were observed when an emerging technology was combined with heating, enzymatic hydrolysis, or another processing step. For example, ultrasound alone produced an approximately 50% decrease in immunoreactivity in a soybean system, whereas its combination with cooking resulted in a reduction exceeding 99% [59]. Similarly, the pronounced decrease in Gly m 5 recognition observed during high-pressure treatment was obtained during HPP-assisted enzymatic hydrolysis rather than through pressure alone [66]. Although these observations indicate that combined treatments warrant further investigation, they do not demonstrate that sequential processes are inherently more effective than individual treatments or that the reported reductions reflect true technological synergy. In most cases, the available experimental designs did not allow the contribution of each processing step to be separated or distinguish conclusively among epitope modification and aggregation-induced masking.

Finally, the strength and biological relevance of the conclusions depend on the specific immunological endpoint used to characterize the response to processing. IgE-binding assays using sera from sensitized individuals provide evidence to consider for the recognition of food allergens by antibodies involved in IgE-mediated allergy [39,60], although reduced IgE binding alone does not demonstrate a reduced clinical allergic response in vivo. IgG-based assays, animal antisera, and monoclonal antibodies address a different level of evidence and primarily reflect antigenicity, epitope accessibility, or analytical detectability rather than clinical allergenicity [22,23,81]. In gluten-containing cereals, R5 and G12 assays constitute a further distinct category because they quantify the recognition of selected gluten-related epitopes used for gluten detection and assessment; changes in R5/G12 reactivity may therefore indicate altered epitope accessibility or analytical gluten detectability, but they do not establish reduced T-cell-mediated immunogenicity or safety for individuals with celiac disease [15,24]. These three immunological readouts (IgE binding, non-IgE antibody recognition, and R5/G12 gluten reactivity) were therefore interpreted separately rather than as equivalent forms of direct immunological evidence. Structural, proteomic, digestive, and techno-functional approaches remain essential for elucidating the molecular mechanisms through which processing modifies proteins, but they provide complementary rather than clinically demonstrative evidence. Collectively, the available evidence consistently demonstrates that emerging processing technologies can modulate protein structure, digestibility, functionality, IgE recognition, non-IgE antigenicity, and gluten-epitope detectability; however, the current literature does not establish that any specific technology generally produces clinically hypoallergenic foods or gluten-containing cereal products that are safe for individuals with celiac disease. This distinction provides the framework for interpreting the methodological and translational challenges discussed in the following section.

## 4. Current Challenges and Future Research Directions

A fundamental limitation of the current evidence base is the heterogeneity and unequal representation of food matrices, protein systems, and processing conditions. Evidence is strongly concentrated on soybean and wheat, whereas other legumes and gluten-containing cereals, particularly barley and rye, remain insufficiently investigated. Furthermore, many studies have used purified proteins, isolates, or simplified model systems, whose responses may differ substantially from those observed in intact food because of interactions with starch, lipids, fiber, and other matrix constituents. Differences in hydration, physical state, protein accessibility, and matrix organization have already produced contrasting responses to technologies such as HPP, pulsed light, and cold plasma [24,48,89]. Consequently, findings obtained in purified or highly accessible protein systems should not be directly extrapolated to intact foods or other species.

Future investigations should therefore encompass a broader diversity of species, varieties, and realistic food matrices and should systematically report variables such as hydration state, sample geometry, protein concentration, and physical state.

At the first level of evidence, structural, proteomic, digestive, and techno-functional measurements, are essential for determining how processing modifies allergenic and immunoreactive proteins. However, these approaches remain mechanistic or indirect with respect to allergenicity and gluten-related immunogenicity. Protein unfolding, fragmentation, aggregation, cross-linking, oxidation, hydrolysis, changes in sulfhydryl-disulfide balance, or disappearance of electrophoretic bands can indicate substantial molecular modification without demonstrating that immunologically relevant epitopes have been eliminated. This distinction becomes particularly important after processing because aggregation, cross-linking, or decreased solubility may redistribute proteins toward insoluble fractions and reduce their analytical recovery without necessarily eliminating antibody-recognized regions [15,25]. Future studies should therefore standardize extraction procedures, quantify protein recovery, and analyze soluble and insoluble fractions simultaneously. Structural and proteomic approaches should be integrated with gastrointestinal digestion and immunological measurements to distinguish genuine epitope alteration or degradation from conformational masking, aggregation-induced inaccessibility, and reduced extractability.

Mechanistic evaluation should also consider nutritional and techno-functional consequences. Structural transformations associated with altered antibody recognition may simultaneously affect solubility, digestibility, water- and oil-holding capacity, emulsification, foaming, gelation, and rheological behavior. These responses do not necessarily change in parallel with immunoreactivity, and treatments associated with lower antibody recognition may also promote aggregation, loss of solubility, or other undesirable technological effects [25,75,80]. Process optimization should therefore consider molecular modification, nutritional quality, digestibility, functionality, and final product acceptability together rather than focusing exclusively on maximizing reductions in immunochemical signals.

The next level of evidence concerns direct measurement of antibody or epitope recognition; a major challenge is the lack of standardized immunological assessment procedures and the frequent comparison of assays that address fundamentally different biological endpoints. ELISA, Western blotting, and related immunoassays can detect changes in antibody recognition, but their results depend on antibody specificity, extraction procedure, protein recovery, assay format, and epitope accessibility. Reduced immunoassay signals should therefore be interpreted within the analytical context in which they were obtained.

Importantly, IgE binding, non-IgE antibody recognition, and R5/G12 gluten reactivity should be evaluated separately; IgE-binding assays using sera from sensitized individuals provide evidence directly relevant to antibody recognition in IgE-mediated food allergy, although they do not independently reproduce an in vivo allergic reaction or predict clinical severity. In contrast IgG-based assays, animal antisera, and monoclonal antibodies primarily provide information on antigenicity, epitope accessibility, or analytical detectability. R5 and G12 assays represent a further distinct category because they assess the recognition of selected gluten-related motifs and should not be interpreted as direct evidence of reduced celiac-related immunogenicity or safety. Future studies should therefore adopt standardized immunoassay and extraction protocols, clearly report antibody source and specificity, and use well-characterized serum panels when investigating IgE-mediated food allergy.

Comparability among studies using human sera is further limited by differences in the number of sensitized individuals included, patient-selection criteria, clinical phenotype, allergen-specific IgE levels, and the use of individual versus pooled sera. These factors can substantially influence the magnitude and variability of IgE-binding responses and should therefore be systematically reported and considered when comparing processing-induced changes across studies. Changes in IgE recognition, non-IgE antigenicity, and R5/G12 reactivity should be reported and interpreted as separate outcomes rather than combined into a single category of immunological response.

A higher level of evidence is required to determine whether processing-induced molecular or analytical changes translate into biologically meaningful effects. The current literature remains predominantly based on in vitro measurements, whereas cellular and animal models are scarce and clinical validation is virtually absent. Animal models can provide information on sensitization, systemic responses, and inflammatory outcomes, but their direct extrapolation to human food allergy remains limited by physiological and immunological differences [31]. Future investigations should therefore progress beyond antibody recognition by integrating physiologically relevant gastrointestinal digestion with characterization of residual epitopes and appropriate cellular, ex vivo, or other biological models.

When animal experimentation or human challenge studies are not feasible or ethically justified, well-characterized human-serum assays combined with digestion-integrated cellular or ex vivo models may provide an ethically preferable intermediate or “second-best” level of evidence. Such approaches cannot replace clinical confirmation, but they can substantially strengthen biological interpretation beyond purely structural or analytical measurements.

Combined treatments represent both an opportunity and an important methodological challenge; some of the largest reported decreases in antibody or epitope recognition were observed when emerging technologies were combined with heating, enzymatic hydrolysis, or other processing steps [59,66]. However, these observations do not establish that combined processes are inherently more effective than individual treatments or that the reported effects represent true technological synergy. Multi-step interventions frequently make it difficult to determine which operation is responsible for the observed response. This issue is particularly relevant when the emerging process itself generates substantial heating, as may occur during microwave processing, UHPH, PEF, or ohmic heating. Future studies should therefore incorporate appropriate treatment-alone controls, conventionally heated samples matched for time-temperature history, and factorial designs evaluating individual treatments and their combinations.

The final level of evidence concerns translation from laboratory observations to realistic food systems, industrial processing, and ultimately safety-related interpretation. Many included studies used small sample volumes, purified proteins, isolates, or treatment conditions that do not fully reproduce the temperature gradients, mass and energy transfer, residence times, matrix heterogeneity, and equipment constraints encountered during large-scale processing. Consequently, effects demonstrated in laboratory systems cannot be assumed to remain unchanged during pilot or industrial-scale operation. Future research should therefore assess reproducibility at progressively larger scales while considering energy consumption, throughput, equipment costs, process control, storage stability, nutritional quality, and sensory attributes. Validation should also be performed in realistic food matrices rather than relying exclusively on purified proteins or simplifies model systems.

From a regulatory and safety perspective, the level of evidence required should be proportional to the claim being made. A reduction in ELISA, Western blotting, R5/G12, or other immunoassay signals should not alone support claims of reduced allergenicity, reduced celiac-related immunogenicity, or improved safety for susceptible populations. Accordingly, future research should aim not merely to demonstrate that emerging processing technologies modify proteins, but to establish, through increasingly robust levels of evidence, whether those modifications have biologically and clinically meaningful consequences.

## 5. Conclusions

This systematic review shows that emerging processing technologies can alter the molecular structure, gastrointestinal digestibility, techno-functional behavior, and analytical immunoreactivity of proteins from legumes and gluten-containing cereals. The evidence remains heavily concentrated on soybean and wheat, leaving most other legumes, as well as barley, rye, oats, and less widely consumed wheat species, insufficiently characterized. Decreases in analytical antibody or epitope recognition were reported more frequently in legume-based systems, whereas gluten-containing cereals, particularly wheat, showed more heterogeneous responses ranging from decreased recognition to no significant change or increased recognition under specific processing conditions. However, these outcomes were derived from immunological assays with different biological meanings, including IgE binding, non-IgE antibody recognition, and R5/G12 gluten reactivity, and therefore should not be interpreted as equivalent evidence of reduced allergenicity or immunogenicity. The observed responses were influenced by the target protein, matrix composition, treatment intensity, extraction procedure, and analytical method used.

Protein unfolding, fragmentation, aggregation, oxidation, hydrolysis, and secondary-structure rearrangement were widely reported across the investigated technologies, but the extent of structural modification did not consistently predict the direction or magnitude of antibody recognition. Processing-induced changes may alter or degrade immunoreactive sequences, mask epitopes within aggregates, or expose previously inaccessible regions of the protein, which explains why pronounced conformational changes do not necessarily result in lower immunoreactivity. Structural, proteomic, digestive, and techno-functional measurements are therefore valuable for explaining processing mechanisms, but they cannot independently establish a reduction in allergenic or gluten-related immunogenic potential.

Combined treatments were associated with some of the largest reported decreases in analytical antibody or epitope recognition, particularly when emerging technologies were coupled with heating, enzymatic hydrolysis, fermentation, or other processing steps. These findings suggest that sequential or integrated processes may be more effective than isolated treatments, although most studies did not include experimental designs capable of confirming synergy or separating the contribution of each operation. Thermal controls matched for time and temperature, factorial designs, and measurements of protein recovery in both soluble and insoluble fractions are needed to determine whether the observed reductions arise from epitope degradation, aggregation-induced masking, or reduced analytical extractability.

The current evidence demonstrates more consistently that emerging processing technologies modify the molecular, digestive, techno-functional, and analytical properties of allergenic and immunoreactive proteins than that they reduce biologically or clinically relevant immune responses. Of the 95 included studies, only 28 incorporated antibody based immunological measurements, whereas 70.5% relied exclusively on structural, digestive, proteomic or techno-functional outcomes, and none provided clinical confirmation of reduced allergic reactions or celiac-related risk. Moreover, the immunological evidence itself was heterogeneous: IgE-binding assays using sera from sensitized individuals, non-IgE antibody assays, and R5/G12-based gluten measurements address distinct biological or analytical endpoints and should not be assigned equivalent evidentiary weight.

In this context, well-characterized human serum assays combined with physiologically relevant cellular, ex vivo, or digestion-integrated models may provide an ethically preferable intermediate or “second-best” level of evidence when animal experiments or human challenge studies are not feasible or ethically justified. Although such approaches cannot replace clinical confirmation, they can substantially strengthen biological interpretation beyond purely structural or analytical measurements.

Future studies should adopt standardized extraction and immunoassay protocols, use well-characterized human serum panels when investigating IgE-mediated food allergy, evaluate residual immunoreactive epitopes after gastrointestinal digestion, and incorporate cellular or biological models in realistic food matrices. For wheat, IgE-mediated allergy and celiac-related gluten immunogenicity must be investigated and interpreted separately. This progression from molecular characterization to biologically relevant validation is necessary before processing-induced reductions in immunoreactivity can support claims of lower allergenic risk or improved safety for susceptible populations.

## Figures and Tables

**Table 1:** Major allergenic and immunoreactive proteins evaluated in legumes and gluten-containing cereals.

Food Group/Matrix	Protein/Allergen or Protein Fraction	Reported Content, Concentration, or Activity *	Enzymatic Activity	Biological/Immunological Relevance	References
Legumes					
Soybean	7S β-conglycinin (Gly m 5) and 11S glycinin (Gly m 6)	0.016–2.8%	NR	Major IgE-reactive soybean storage allergens	[11,22,23,32,33,34,36,37,59,60,61,62,63,64,65,66,67,68,69,70,71,72,73,74,75,76,77,78,79,80,81,82]
	Gly m 1–4, Gly m 7, Gly m 8, P34/Gly m Bd 30K, lipoxygenase, agglutinin, and β-amylase	lipoxygenase: 0.3–0.8%	NR	Additional allergenic or immunoreactive soybean targets	[32,33,34,35,36,59,60,83]
	Kunitz trypsin inhibitor and related protease inhibitors	3.094–9.368%	522.40 TIU∙mg^−1^ dry and 272.82 TIU∙mg^−1^ after soaking	Allergenic and antinutritional proteins	[23,32,33,34,35,36,37,38,39]
Pea	Legumin, vicilin, convicilin, albumins, lectin, and lipoxygenase	Lectin: 0.55%	NR	Storage proteins and antinutritional factors; Vicilin and convicilin are clinically relevant IgE-binding pea allergens	[52,53,54,55,56,57,58,84,85,86]
Common and kidney bean	Albumins, vicilin-type and legumin-type globulins, and trypsin inhibitors	NR	171.28 TIU∙mg^−1^ dry and 90.53 TIU∙mg^−1^ after soaking	Storage proteins and antinutritional inhibitors	[12,35,84,87]
Mung bean	Vig r 2, Vig r 4, and vicilin- and legumin-type globulins		NR	Selected proteins evaluated for IgE reactivity and structural modification	[87,88]
Peanut	Ara h 1, Ara h 2, Ara h 3, and Ara h 4	NR	NR	Major clinically relevant peanut allergens	[77,89,90]
Lupin	α-, β-, γ-, and δ-conglutins and 2S albumin	NR	NR	Recognized or potential lupin allergens	[91,92]
Chickpea	7S vicilin, 11S legumin, and Kunitz-type trypsin inhibitor	NR	63.92 TIU∙mg^−1^ dry and 35.71 TIU∙mg^−1^ after soaking	Storage globulins and antinutritional inhibitor	[35,93]
Cowpea	Kunitz-type trypsin inhibitor	NR	166.18 TIU∙mg^−1^ dry and 107.68 TIU∙mg^−1^ after soaking	Residual antinutritional activity; not a direct measure of immunoreactivity	[35]
Lentil	11S legumin, 7S vicilin, and γ-vicilin	NR	NR	Structural and functional characterization; Major IgE-binding lentil (Len c1 vicilin)	[94]
Guamuchil (*Pithecellobium dulce*)	Glutelin, albumin, globulin, and prolamin fractions	61.10%, 26.92%, 9.16%, and 1.85%, respectively	NR	Compositional protein fractions; allergenicity was not directly assessed	[95]
Cereals					
Wheat	α/β-, γ-, and ω-gliadins; HMW and LMW glutenin subunits	Gliadins: 1.29–2.21%; glutenins: 2.52–3.36%; immunoreactive gliadins: 22.75% gluten	NR	Major fractions associated with wheat allergy and gluten-related immune responses	[15,24,40,41,42,43,44,45,46,47,48,49,50,51,96,97,98,99,100,101]
	Albumins and globulins	NR	NR	Non-gluten proteins associated with food and respiratory wheat allergy	[40,102]
	Wheat germ agglutinin	NR	NR	Antinutritional lectin evaluated in sensitized models	[31]
Oat	Avenalin and other oat-protein fractions	NR	NR	Evidence focused mainly on structural and techno-functional changes	[103,104]

Note: NR, not reported; HMW, high molecular weight; LMW, low molecular weight; TIU, trypsin inhibitor units. Reported contents and activities were determined using different food matrices, extraction procedures, analytical methods, and expression bases and are therefore not directly comparable. * Allergenic relevance was assigned only when supported by IgE-binding, sensitization, clinical, or recognized allergen evidence; enzymatic inhibitory activity alone was not considered evidence of allergenicity.

**Table 2:** Analytical and biological approaches used to assess allergenic and immunoreactive proteins.

Method	Primary Endpoint Assessed	Main Advantage	Main Limitation	References
In vitro				
ELISA: sandwich, indirect, and competitive formats	Quantification of antibody-reactive proteins or epitopes; assessment of antigenicity or IgE binding	Sensitive, adaptable to different matrices, and potentially quantitative	Results depend on antibody specificity, protein extractability, and epitope accessibility; clinical allergenicity is not directly measured	[15,22,23,24,25,26,36,37,39,59,60,61,62,66,68,75,80,81,85,89,90,96,97,98,101,105,106]
Western blotting	Detection of antibody-binding bands and estimation of apparent molecular weight	Links antibody binding to specific molecular-weight protein fraction	Mainly semiquantitative and affected by extraction, electrophoresis, transfer efficiency, and antibody quality	[80,89,90,96,101,105]
Dot blotting	Rapid qualitative detection of antibody reactivity	Simple, rapid, inexpensive, and requires little sample	No protein separation, low resolution, and mainly qualitative	[90]
Structural and proteomic (SDS-PAGE, RP-HPLC, SEC-HPLC, LC-MS/MS, FTIR, Raman spectroscopy, and circular dichroism)	Protein profiles, molecular-weight distribution, aggregation, peptide identification, and conformational changes	Provides mechanistic information on processing-induced modifications	Does not directly assess IgE binding, sensitization, or clinical allergenicity	[15,75,87,107]
Simulated gastric or gastrointestinal digestion	Protein degradation and persistence of potentially immunoreactive fragments	Approximates gastric and intestinal conditions	Protocol heterogeneity; digestion may destroy existing epitopes or generate new immunoreactive fragments	[22,66,108]
Trypsin inhibitor activity assays, including BANA and multienzyme methods	Residual protease-inhibitory activity	Sensitive to processing-induced inactivation of antinutritional factors	Does not directly measure antigenicity, IgE binding, or clinical allergenicity	[35,38]
Biological/ex vivo				
Bovine polyclonal antisera raised against soybean protein	Systemic antibody response followed by serum-based assessment	Incorporates prolonged dietary exposure and biological variability	Species-specific response, high complexity, and limited extrapolation to human food allergy	[81]
In vivo				
Sensitized murine models	Sensitization, systemic immune responses, and cross-reactivity	Provides integrated biological evidence beyond analytical detection	Physiological differences from humans, ethical considerations, cost, and limited clinical extrapolation	[31]

Note: ELISA, enzyme-linked immunosorbent assay; SDS-PAGE, sodium dodecyl sulfate–polyacrylamide gel electrophoresis; RP-HPLC, reversed-phase high-performance liquid chromatography; SEC-HPLC, size-exclusion high-performance liquid chromatography; LC-MS/MS, liquid chromatography–tandem mass spectrometry; FTIR, Fourier-transform infrared spectroscopy; BANA, Nα-benzoyl-DL-arginine-β-naphthylamide. IgE-binding assays provide direct evidence of immunoreactivity but not proof of clinical allergenicity. Structural, digestive, and functional outcomes were interpreted as complementary or indirect evidence.

**Table 3:** Processing conditions and effects of ultrasound on the immunoreactivity, structure, digestibility, and techno-functional properties of proteins from legumes and gluten-containing cereal matrices.

Food Matrix	Target Protein(s)	US Conditions and Complementary Treatment	Direct Immunological Evidence	Structural or Digestive Changes	Functional and Bioactive Changes	Ref.
Soybean slurry and germinated system	Gly m 1–8 allergen profile	20–60 min; germination for 96–120 h; US alone or US + cooking	↓ Immunoreactivity: approximately 50% after 60 min of US; ↓ immunoreactivity: >99% with US + cooking	ND; subsequent cooking probably contributed to protein denaturation	↑ Phenolic compounds and antioxidant activity	[59]
Germinated soybean sprouts	Immunoreactive soybean proteins, trypsin inhibitor, and lipoxygenase	100–300 W; 30 min; 25 °C	↓ Immunoreactive signal assessed using human serum: approximately 51%; residual immunoreactivity remained	Modified secondary structure; rearrangement of disulfide bonds	↓ Trypsin inhibitor activity: 98.78%; ↓ lipoxygenase I activity: 36.22%; ↓ lipoxygenase II + III activity: 55.57%	[39]
Soybean protein isolate	β-Conglycinin, glycinin, lipoxygenase, and P34/Gly m Bd 30K	20 kHz; 600 W; 50% amplitude; 15 min; 20 °C	↓ IgE binding: 18.9%	Slight ↑ β-sheet content; ↑ exposure of hydrophobic regions: 25.21%; ↔ α-helix content	↑ Zeta potential	[60]
Soybean flour, isolate, and milk	β-Conglycinin and glycinin	300 W; 1–10 min; 4–23 °C; ultrasound-assisted extraction	± ELISA signal depending on the matrix; reflected analytical recovery or detectability rather than demonstrated reduction in immunoreactivity	Solubilization and possible unfolding during extraction	↑ Protein recovery; ↓ extraction time	[61]
Soybean okara	11S glycinin and 7S β-conglycinin	750 W; 75% amplitude; 10 min; pH 9.0; 30/15-s pulsed treatment	ND	Selective insolubilization of the 11S fraction; ↓ fiber-bundle size: >30 → 3–5 μm	↑ Protein content: 35.3 → 63.3%; ↑ emulsifying activity	[67]
Soybean flour	β-Conglycinin, glycinin, trypsin inhibitor, β-amylase, and agglutinin	0–500 W; 0–20 min; ultrasound-assisted isopropanol extraction	ND; depletion of proteins from the extract reflected extraction selectivity rather than allergen elimination	↑ Cell-particle disruption; ↑ selective protein release	↑ Recovery of low-abundance proteins	[33,34]
Soybean 7S and 11S fractions	β-Conglycinin and glycinin	20 kHz; 100–750 W; 5–50 min; 40–60 °C or ice-bath control	ND	± Structural response depending on the protein fraction; ↑ ·OH radicals: up to 2.30–3.68 μM; ↓ free –SH groups through oxidation under selected conditions; ↓ aggregate size: 75.9 → 51.8 nm	↑ Degree of hydrolysis: 33.6%; ↑ solubility: 85 → 93%	[69,72,79]
Guamuchil protein isolate	Glutelins, albumins, globulins, and prolamins	20 kHz; 200–600 W; 15–30 min; <15 °C	ND	↓ α-Helix content: 10.5 → 6.2%; ↑ β-sheet content: 45.9 → 55.0%; ↑ surface hydrophobicity: 114.8%	↑ Solubility: 115.5%; ↑ in vitro digestibility: 12.5%; ↑ emulsifying activity: 87.4%; ↑ foaming capacity: 74.2%	[95]
Lentil protein isolate	Legumin, vicilin, and γ-vicilin	20 kHz; 150–450 W; 10 min; pH 9.0; ice-bath control	ND	↔ SDS-PAGE profile; modified structural flexibility and thermal stability	↑ Solubility at pH 7: 63.4%; ↑ foaming capacity; ↑ foam stability	[94]
Chickpea protein concentrate	Vicilin and legumin	33–119 W; 10–30 min; 2-s cycles; ice-bath control	ND	↓ Detectability of a 45-kDa band in the hydrolysate; epitope elimination ND	↑ Biological activity of peptide fractions	[93]
Sweet and bitter lupin protein isolates	α-, β-, and γ-conglutins and δ-albumin	20 kHz; 5–30 min; extraction at pH 12 followed by neutralization	ND; ↔ electrophoretic protein profile	↓ Aggregate size; ↑ nanosized protein fraction	↑ Water-holding capacity; ↑ foam stability; ± emulsifying activity depending on the cultivar	[91]
Mung bean protein isolate	Vig r 2, Vig r 4, and β-conglycinin-like proteins	700 W; 30 kHz; 15 min; US + pH-shifting	IgE binding ND; ↓ abundance of Vig r 4 and β-conglycinin-like proteins according to SDS-PAGE and LC-MS/MS	↑ α-Helix content: 28.3 → 72.0%; ↓ particle size: 5300 → 174.6 nm; ↓ zeta potential: −21.6 → −26.3 mV	↑ Solubility: 45.9 → 91.2%; ↓ trypsin inhibitor activity: 0.06 → 0.02 U/mg; ↑ gel firmness	[88]
Pea protein isolate	Pea storage proteins	37 kHz; 160 W; 10–30 min; 60 °C; 18% protein	ND	Structural evidence was insufficient to infer epitope modification	↑ Gel homogeneity, consistency, and viscosity	[50]
Pea–oat protein blend	Pea and oat proteins	20–25 kHz; 200–600 W; 20–40 min; ice-bath control	ND	↓ α-Helix content: 30.85 → 27.51%; ↑ β-sheet content: 27.61 → 30.86%; ↑ disulfide bonds: 12.12 → 19.83 μmol·g^−1^	↑ Gel strength; ↑ thermal stability: 89.7 → 110.9 °C; ↑ viscoelasticity	[104]
Wheat noodle dough	Gliadins and glutenins	20 kHz; 300 W; 5–20 min; 30 °C; 42% moisture	ND	↓ Glutenin macropolymers; ↓ free–SH groups; ↑ disulfide bonds	↑ Extensibility; ↓ resting time: 20 → 10 min; ↑ water redistribution	[112]
Wheat gluten or protein systems	Gliadins and glutenins	37 kHz; 160 W; 10–30 min; 60 °C; US ± transglutaminase	Antibody binding ND; ↓ gliadin content: up to sixfold with US + transglutaminase; ↔ selected α-gliadins	Partial unfolding; modified gliadin/glutenin distribution	↑ Gel homogeneity and strength	[50]
Commercial wheat gluten and whole-wheat flour	Gliadins and glutenins	850 kHz; 10–30 min; 35 °C; US alone or US + lactic acid fermentation	Antibody binding ND; ↓ gliadin content: 25% with US alone and 83.5% with US + fermentation	↓ Molecular size of gliadins: 30–120 → 17–25 kDa; partial degradation of glutenins	↓ Dough hardness and consistency; ↑ free amino acids: 0.87 → 2.12 g·kg^−1^	[111]
Wheat gluten and dough	Gliadins and glutenins	Different 20–80-kHz configurations; 10 min; 25–30 °C; 67 W/L–900 W	ND	↓ α-Helix content; ↑ β-sheet and β-turn structures; ↓ particle size; ↑ hydrophobic exposure	↑ Solubility; ↑ foaming capacity; ↑ emulsifying activity and stability	[42,51,109]
Isolated gliadin from different wheat cultivars	Gliadins of 25–100 kDa	200–600 W; 30 min; 30 °C	ND	↓ α-Helix content; ↑ disordered structures; ↓ particle size: 32.2–42.1%; ↑ free –SH groups: 7.61 → 10.99 µmol·g^−1^	↑ Solubility: 6.6–11.55%; ↑ in vitro digestibility: 11.03–12.75%; ↑ emulsifying activity	[110]
Commercial wheat gluten	Gliadins and glutenins	150–300 W; 10–60 s; 8% dispersion	ND	↓ Protein fractions > 10 kDa; ↑ peptides < 1 kDa: up to 68%; ↓ S–S bonds; ↑ free –SH groups	↑ Soluble-protein recovery: up to 86.5%; ↑ degree of hydrolysis: up to 15.8%	[43]
Wheat gluten during US-assisted freezing	Gliadins and glutenins	20–100 W/L; US applied during different freezing stages	ND	↑ Preservation of α-helix structures; ↑ gluten-network uniformity; ↓ pore size	↑ Viscoelastic moduli; ↓ hardness: 25,706 → 23,118 g	[45]
Isolated wheat gliadin	α/β-, γ-, and ω-gliadins	40 kHz; 240–600 W; 2 h; 30 °C	↓ Antigenicity: maximum of 33.03% at 480 W, assessed by ELISA and Western blotting	↓ α-Helix content; ↓ β-sheet content; modified disulfide-bond conformation; ↑ free –SH groups	ND	[96]

Note: ↑, increase; ↓, decrease; ↔, no significant change; ±, variable or condition-dependent response; →, change from the initial to the final value; ND, not determined or not directly assessed; US, ultrasound; ELISA, enzyme-linked immunosorbent assay; LC-MS/MS, liquid chromatography–tandem mass spectrometry; SDS-PAGE, sodium dodecyl sulfate–polyacrylamide gel electrophoresis. Arrows are placed before the corresponding response variable.

**Table 4:** Processing conditions and effects of ultraviolet, gamma, and electron-beam irradiation on the immunoreactivity, structure, digestibility, and techno-functional properties of proteins from legumes and gluten-containing cereal matrices.

Food Matrix	Target Protein(s)	Irradiation Conditions and Complementary Treatment	Direct Immunological Evidence	Structural or Digestive Changes	Techno-Functional, Nutritional, or Antinutritional Changes	Ref.
Gamma irradiation						
Commercial soybean KTI in aqueous, dry, and 50–moisture systems	Kunitz trypsin inhibitor, KTI/STI	1–200 kGy; KTI: 10 mg·mL^−1^; aqueous, dry, or 50–moisture state	ND; trypsin inhibitor activity was an antinutritional outcome	Aqueous: ↑ unfolding and HMW aggregation; ↓ native form at 10 kGy. Dry: ↔ elution profile up to 100 kGy; ↑ hydrophobicity at 100–200 kGy. 50% moisture: ↑ structural damage and aggregation	Aqueous: ↓ KTI activity by 80–85% at 10 kGy and 100% at higher doses. Dry: ↓ activity by 37–57% at 100–200 kGy. 50% moisture: ↓ activity by 18–100% at 30–200 kGy	[35,36]
Soybean seeds, cultivars Enrei and Fukuyutaka	7S β-conglycinin, Gly m Bd 30K, Gly m 4, and KTI	2.5–30 kGy; 21 ± 2 °C	Enrei: ↓ IgE reactivity at 30 kGy; ↓ KTI reactivity. Fukuyutaka:↓ IgE reactivity at 20–30 kGy	↔ Major-allergen band profile in Enrei; ↓ 90-kDa proteins through fragmentation into approximately 20-kDa fractions at >10 kGy	ND	[36,71]
Soybean storage-protein system	β-Conglycinin and glycinin subunits	0–30 kGy; trypsin inhibitor result reported at 45 kGy	ND	Partial protein degradation at >20 kGy; ↓ acidic glycinin subunits after ruminal incubation; ↔ overall protein profile	↓ Trypsin inhibitor activity by 63.5%; ↑ in vitro protein digestibility by 28%	[63]
Soybean mutant lines, including M12–M20 and VLSoy-2-derived lines	α′, α, and β subunits of β-conglycinin; A3, G1, and G2 glycinin subunits	250 Gy using a ^60Co source	ND; subunit absence was not assessed through antibody binding	Absence or ↓ abundance of selected β-conglycinin and glycinin subunits; mutations in storage-protein loci; altered lipoxygenase bands	↓ Trypsin inhibitor activity in selected lines; ± 11S/7S ratio; ↔ total protein: 39.7–40.5%	[65,77]
Soybean seeds, cultivar Clark	7S β-conglycinin and 11S glycinin	0.5–7.5 kGy	ND	↓ Globulin-band intensity at 7.5 kGy; ↑ selected 11S subunits up to 5 kGy; extensive denaturation at 7.5 kGy	↓ Protein solubility: 76.2 → 65.6%; ↑ SDS-ME-soluble fraction: 10.1 → 15.2%	[77]
Peanut seeds, cultivar Giza 5	Arachin and conarachin	0.5–7.5 kGy	ND	Appearance of a 16-kDa band; ↑ protein oligomerization	↓ Total protein solubility: 82.8 → 60.0%	[77]
Common bean, cowpea, and chickpea seeds	Kunitz-type trypsin inhibitor	1–200 kGy; dry or 50–moisture state	ND	Dry: ↔ structure up to approximately 100 kGy; strong matrix protection. 50% moisture: ↑ degradation due to water radiolysis	Common bean: ↓ activity by 66% at 200 kGy under moist conditions. Cowpea:↓ activity by 57–85% at 100–200 kGy under moist conditions. Chickpea:↔ activity under the reported conditions	[12,35]
Common bean flour, cultivar Giza	Albumin, globulin, prolamin, and glutelin fractions	1–2 kGy; approximately 26 °C	ND; Osborne fractionation was not an immunological assay	↑ Aggregation and cross-linking; ↑ exposure of hydrophobic groups	↓ Albumin: 56.65 → 46.92%; ↑ globulin: 11.7 → 16.2%; ↓ nitrogen solubility: 65.95 → 42.17%; ↓ foaming capacity: 200 → 160 mL·100 g^−1^	[25]
Kidney bean, black gram, and peanut	Gamma alone: 5–25 kGy; gamma + boiling: 25 kGy + boiling	Total soluble and insoluble allergenic proteins	Gamma alone: ↔ IgE binding in all matrices. Gamma + boiling: Kidney bean: ↓ soluble IgE binding by 89.0%; ↓ insoluble IgE binding by 52.9%. Black gram: ↓ soluble IgE binding by 86.6%; ↓ insoluble IgE binding by 79.3%. Peanut: ↓ insoluble IgE binding by 52.1%; soluble result unclear	Gamma alone: ↔ reported structural response. Gamma + boiling: ↑ denaturation and severe aggregation	↓ Protein solubility after the combined treatment	[25]
Wheat germ	Wheat germ agglutinin	1–25 kGy; O_2_ atmosphere; freeze-dried sample	↓ Antigenicity, intestinal-cell binding, lymphocytic infiltration, and allergy-associated cytokines at 10–25 kGy in sensitized mice	↓ Native conformation; ↑ exposure of hydrophobic regions	↓ Hemagglutinating activity and intestinal inflammatory response	[31]
Wheat endosperm, cultivars HD3086 and HD3118	α/β-, γ-, and ω-gliadins	0.20–0.30 kGy applied to dry seeds	IgE binding ND; ↑ gliadin isoforms at 0.20 kGy	↔ Structural integrity at low doses; ± molecular-weight profile and protein accumulation	↑ Seed growth parameters under heat stress; modified antioxidant-defense network	[100]
Wheat grain, cultivars Sids-1 and Sakha-93	Albumins, globulins, gliadins, and glutenins	0–300 Gy; treatments also evaluated under salt stress	ND	↓ Electrophoretic bands: 20 → 13 at 200 Gy; disappearance of a 71.1-kDa band	↑ Gliadin, glutenin, HMW-glutenin, and free amino-acid fractions under selected conditions	[40]
Durum wheat semolina, cultivar Horani	Albumins and globulins	0.25–10 kGy; irradiation before or after milling	ND	↓ Protein bands: 19 → 13–15	↓ Lysine by 14% at 10 kGy; ↔ total protein: 10.6–10.9%	[99]
Commercial wheat gliadin and wheat flour	α/β-, γ-, and ω-gliadins	2.2–12.8 kGy; ambient temperature	Gliadin: ↑ immunoreactivity: 100 → 174.1% at 8.8 kGy; ↑ IgE binding by up to 500% at 12.7 kGy. Flour: ↑ immunoreactivity: 100 → 125% at 8.8 kGy	Gliadin: ω-gliadin fragmentation; ↑ HMW aggregates; ↓ native conformation. Flour:modification of Cys, Tyr, and Trp residues	↓ RP-HPLC peak detectability at high doses; matrix-associated ↑ antibody recognition	[44,101]
Ultraviolet irradiation						
Hard red wheat flour and flatbread	α/β-, γ-, and ω-gliadins; HMW- and LMW-glutenins	UV, 0–120 min, 15 cm; flour treated with UV alone or UV + IR; flatbread exposed after baking	Flour: ↓ G12-reactive signal by 58–64%. UV + IR: ↓ signal by up to 50% at 30 min; ± response thereafter. Flatbread: ↓ signal by up to 60% at 60 min; smaller response than in raw flour	Flour: ↓ approximately 50-kDa gliadin bands; ↑ S–S oxidation. UV + IR: ↓ gliadin-band intensity at 30 min; ↑ intensity at 60–120 min. Flatbread: ↔ gliadin/glutenin profile	↓ Wet gluten: 32.9 → 25.8%; ↓ gluten-network formation; ↑ brittleness. Previous baking ↓ effectiveness of subsequent UV treatment	[26]
Punjab wheat flour and isolated gluten	Gliadins and glutenins	1.5–4.5 W; 50–250 s; energy input ≤ 45 J/g	ND	Photoinduced –SH/S–S interchange. Flour: ↓ α-helix; ↑ β-sheet and β-turn. Gluten: ↑ helical/turn structures; ↓ β-sheet	↓ Gluten-network-forming capacity; ↑ water-binding capacity: 75.6 → 85.1%	[113]
Electron-beam irradiation						
Native and roasted soybean flour	7S β-conglycinin, 11S glycinin, trypsin inhibitor, and lipoxygenase	4.8–21.2 kGy; 8.3 MeV; native or roasted flour	ND; SDS-PAGE and enzyme activities did not directly assess antibody binding	↓ 7S subunits; smaller changes in 11S; ↑ fragmentation of large 7S polypeptides; ↑ 11S/7S ratio in roasted flour: 1.65 → 2.29	Native flour: ↓ PDI: 54.9 → 43.2%; ↓ trypsin inhibitor: 65.1 → 52.1 mg·g^−1^. Roasted flour: ↓ trypsin inhibitor: 10.2 → 6.5 mg·g^−1^; ↔ PDI. ↑ In vitro digestibility: 54.03 → 56.21%; ↓ solubility: 90.1 → 86.0%	[32,108]

Note: ↑, increase; ↓, decrease; ↔, no significant change; ±, variable or condition-dependent response; →, change from the initial to the final value; ND, not determined or not directly assessed; HMW, high molecular weight; IR, infrared; KTI, Kunitz trypsin inhibitor; LMW, low molecular weight; PDI, protein dispersibility index; S–S, disulfide bond; –SH, sulfhydryl group.

**Table 5:** Processing conditions and effects of microwave treatment on the immunoreactivity, structure, digestibility, and techno-functional properties of proteins from legumes and gluten-containing cereals matrices.

Food Matrix	Target Protein(s)	Processing Conditions and Complementary Treatment	Direct Immunological Evidence	Structural or Digestive Changes	Techno-Functional, Nutritional, or Antinutritional Changes	Ref.
Raw soybean flour and soybean protein isolate	7S β-Conglycinin and 11S glycinin	600 W; 2450 MHz; 60–100 °C; 5–10 min; microwave-assisted extraction	Flour: ↓ ELISA-detected protein recovery: 28.99 → 19.51 mg·mL^−1^ at 60 °C. Isolate: ↓ detected recovery: 2.39 → 0.88 mg/mL at 100 °C. These results reflect analytical extraction or detectability, not demonstrated mitigation of immunoreactivity	↑ Denaturation, unfolding, aggregation, and covalent-complex formation	Flour: ↑ PBS solubility: 31 → 53%. Isolate: ↑ urea-buffer solubility to approximately 60%	[61]
Glycosylated soybean 7S globulin	α, α′, and β subunits of β-conglycinin	900 W; 0–120 s; sample thickness: approximately 1 mm	ND	At 60 s: ↑ α-helix: 19.68 → 47.86%; ↓ β-sheet: 28.39 → 12.33%; ↑ particle size: 124.8 → 152.3 nm	↑ Water-holding capacity: 4.18 → 6.06 g·g^−1^; ↑ emulsifying activity; ↓ oil-holding capacity: 25.3 → 22.7 g·g^−1^	[74]
Protein-rich legume powder	Predominantly 11S globulin fractions	Microwave drying; inlet temperature: 140–200 °C; solids: 15–50%; feed rate: 3–21 mL/min	ND	↓ α-Helix; ↑ β-sheet; altered 18–44-kDa fractions; ↑ protein aggregation	↑ Dispersibility with temperature; ↑ solubility with lower residual moisture	[83]
Edamame milk	Trypsin inhibitor and soybean proteins	1000 W; 70–100 °C; 5–15 min	ND; trypsin inhibitor activity was an antinutritional outcome	↓ α-Helix: 15.41 → 6.84%; ↓ random coil: 34.46 → 23.22%; modified amide I region	↓ Trypsin inhibitor activity by 74%: 30.94 → 8.07 mg·g^−1^; ↑ in vitro protein digestibility: 81.05 → 86.60%	[38]
Wheat gluten hydrolysate	Gliadins and glutenins	200–800 W; 50–100 °C; 1 min; 10% protein; microwave pretreatment + enzymatic hydrolysis	↓ R5-reactive gluten signal by approximately 60%, reaching 39.65% of the control under selected conditions; effect cannot be attributed exclusively to microwave treatment	↑ Reactive and total –SH groups; ↓ α-helix; ↑ β-sheet and aggregation; ↔ primary electrophoretic profile	↑ Emulsifying activity: 11.63 → 50.66 m^2^·g^−1^; ↑ ABTS activity: 7.5 → 70.3%	[106]
Wheat gluten powder	Gliadins and glutenins	200–1000 W; 20–60 s	↑ R5-reactive gluten signal: 227.5 → 298.8 g·kg^−1^	↓ α-Helix; ↑ polymer disaggregation; ↓ aggregate-mass fraction	↑ Maximum resistance: 64.63 → 142.17 g; ↓ extensibility	[98]
Medium-strength wheat flour	α/β-, γ-, and ω-gliadins	700 W; 2450 MHz; approximately 37–100 °C; 1–10 min	↔ IgG immunoreactivity; no quantifiable reduction	Slight ↓ 27–30-kDa bands after 10 min; ↔ reactivity of an approximately 35-kDa band	ND	[97]
Commercial gliadin and wheat flour	α/β-, γ-, and ω-gliadins	70–500 W; 1–5 min; 2450 MHz; energy input ≤ 150 kJ	Gliadin: ↑ antibody-binding signal by a reported 210% at 40 kJ; return toward the control at 90 kJ. Flour: ↑ reactivity, with a maximum at 30 kJ	Gliadin: ↓ gliadin fractions with increasing energy; partial degradation and loss of protein integrity. Flour: ↑ unresolved HMW aggregates	ND	[105]
Whole wheat grain, cultivar Begra	Albumin fraction and enzyme inhibitors	15–180 s; final temperature: 28–98 °C; 2450 MHz	ND for human immunoreactivity; ↓ insect-directed inhibitory activities by 78–96%	↓ Extractable proteins by approximately 45%; loss of selected albumin and β-amylase bands	↓ Breadmaking score: 144 → 18; ↓ vitreousness and wet-gluten content	[102]
Hydrated wheat gluten	Gliadins and glutenins	350–706 W; 1–3 min; 65% moisture	ND	Appearance of an approximately 84-kDa band after 3 min; ↔ protein profile below 55 kDa	↓ Solubility with treatment time; ↑ foam stability at the highest power	[49]

Note: ↑, increase; ↓, decrease; ↔, no significant change; ND, not determined or not directly assessed; →, change from the initial to the final value; HMW, high molecular weight; PBS, phosphate-buffered saline; –SH, sulfhydryl groups. ELISA- or immunoblot-based changes indicate altered analytical recognition of selected epitopes and do not independently establish clinical hypoallergenicity. Trypsin inhibitor and insect-enzyme inhibitor activities were treated as antinutritional or biological outcomes rather than direct evidence of allergenicity.

**Table 6:** Processing conditions and effects of cold plasma on the immunoreactivity, structure, and techno-functional properties of proteins from legumes and gluten-containing cereals matrices.

Food Matrix	Target Protein(s)	Cold-Plasma Conditions and Complementary Treatment	Direct Immunological Evidence	Structural Changes	Techno-Functional Changes	Ref.
Soy protein isolate	7S β-Conglycinin (Gly m 5) and 11S glycinin (Gly m 6)	DBD; 40 kV; 80–120 Hz; 1–10 min; aqueous protein system; reported temperature increase < 3 °C	↓ IgE binding by up to 75% at 120 Hz for 5 min; ± response with treatment intensity; ↑ IgE binding after 10 min	↑ Protein carbonyls; ↓ free –SH groups; ↓ α-helix; ↑ β-strand and random-coil structures; ↑ aggregation at prolonged exposure	↑ Solubility at pH 4.5 by up to 282%; ↑ EAI by 56–168%; ↑ FC by 60–194%	[75]
Purified soybean glycinin	Acidic and basic glycinin chains	DBD; 40 kV; air; 2–5 min; protein concentration: 2 mg/mL	↓ IgG binding by 91.64% and ↓ IgE binding by 81.49% after 5 min	↑ Polypeptide cleavage and peptide-fragment formation; oxidation of Trp, Tyr, and Phe; ↓ α-helix: 25.77 → 11.06%	↑ Solubility: 10.78 → 65.96%; ↑ EAI: 21.08 → 160.29 m^2^/g	[80]
Soy protein isolate	Glycinin and β-conglycinin	DBD; 40 kV; 12 kHz; 20–120 s; optimal gelling response reported at 40 s	ND	↑ Carbonyls: 2.39 → 4.36 μmol·g^−1^ at 120 s; ↓ free –SH: 4.49 → 3.25 μmol·g^−1^; ↓ α-helix: 25.7 → 20.4%; ↑ β-sheet: 30.7 → 41.1%	↑ Gel hardness by 2.63-fold and ↑ adhesiveness by 5.98-fold at 40 s	[76]
Defatted peanut flour and whole peanut kernels	Ara h 1 vicilin	80 kV; air; 80% relative humidity; 0–60 min	Defatted flour: ↓ Ara h 1 antigenicity by 43–44% after 60 min. Whole kernels: ↓ antigenicity by only 9.3%	Flour: ↓ α-helix; modified conformational epitopes; ↔ SDS-PAGE profile. Whole kernels: limited secondary-structure changes; matrix and lipid protection	ND	[89]
Grass pea protein isolate	11S legumin, 7S vicilin, and convicilin	DBD; 9.4 or 18.6 kVpp; 20 kHz; 30–60 s; electrode gap: 4 mm	ND	↑ Carbonyls; ↓ free –SH by 33.5–45.5%; ↑ dityrosine by 15.25%; globulin dissociation; slight ↑ α-helix and β-sheet contents	Slight ↑ solubility: 56.02 → 58.96%; ↑ emulsion stability against creaming	[55]
Pea protein concentrate	Legumin, vicilin, and convicilin	Cold-plasma jet; air, N_2_, or He; 6 kV; 2.4 MHz; 5 min; 4 or 8 L/min	ND	Gas-dependent conformational changes; ↓ α-helix: 17.47 → 15.82% with air at 8 L·min^−1^; ↑ disordered structures	Air: ↑ solubility by approximately 36–44%. Air and N_2_: ↑ WHC. All gases: ↑ OHC	[54]
Whole wheat kernels, cultivar Bainong 307	Gliadins and glutenins	Non-thermal plasma; 100–500 W; 10–50 s; selected condition: 500 W for 20 s; grain moisture: 14%	ND	↓ α-helix: 10.26 → 9.40%; ↑ β-sheet: 28.10 → 30.95%; ↑ porosity and fracture of the gluten microstructure	↑ Wet-gluten content to a maximum of 32.63%; ↑ dough stability to 5.60 min; ↓ PPO activity: 11.41 → 5.76 U·g^−1^·min^−1^	[107]
Hard and soft spring wheat flours	Gliadins and glutenins	Atmospheric cold plasma; 70 kV; 7 min	ND	Hard flour: ↓ free –SH: 17.5 → 9.8 nmol·mg^−1^. Soft flour: ↓ free –SH: 13.9 → 9.5 nmol·mg^−1^. Both matrices: ↑ S–S cross-linking and protein polymerization	Hard/soft flour: ↑ G′ to 57.8/39.3 kPa; ↓ Jmax from 247.13/435.22 × 10^−3^ to 63.45/80.31 × 10^−3^ Pa^−1^; ↑ η_0_ from 0.44/0.26 to 2.62/2.19 MPa·s	[47]
Oat protein–casein complex	Oat protein, including an approximately 54-kDa subunit	Atmospheric cold plasma + pH shifting; air at 30 L·min^−1^; 1–3 min; nozzle distance: 40 mm	ND; the study did not evaluate oat-protein immunoreactivity	↑ α-helix: 12.16 → 12.80%; ↓ β-sheet: 28.29 → 26.95%; ↑ surface hydrophobicity: 236.12 → 282.65 RFI	↑ Solubility by 14.49%; ↓ emulsion-droplet size: 5.36 → 3.07 μm	[103]

Note: ↑, increase; ↓, decrease; ↔, no significant change; ±, variable or treatment-dependent response; →, change from the initial to the final value; ND, not determined or not directly assessed; DBD, dielectric barrier discharge; EAI, emulsifying activity index; FC, foaming capacity; G′, elastic modulus; Jmax, maximum creep compliance; OHC, oil-holding capacity; PPO, polyphenol oxidase; RFI, relative fluorescence intensity; –SH, sulfhydryl groups; S–S, disulfide bonds; WHC, water-holding capacity; η_0_, zero-shear viscosity.

**Table 7:** Processing conditions and effects of extrusion cooking on the immunoreactivity, structure, digestibility, and techno-functional properties of proteins from legumes and gluten-containing cereals matrices.

Food Matrix	Target Protein(s)	Extrusion Conditions and Complementary Treatment	Direct Immunological Evidence	Structural or Digestive Changes	Techno-Functional, Nutritional, or Antinutritional Changes	Ref.
Ground soybean hypocotyl	7S β-Conglycinin subunits; 11S glycinin subunits; approximately 33–34-kDa allergenic protein	Twin-screw extrusion; 72–143 °C; 280 rpm; feed rate: 51.4 kg·h^−1^; 2-mm die	↓ Overall antigenicity by up to 99%, although residual antibody recognition remained	Loss of detectable α, α′, and β subunits of 7S and A3 of 11S; A1A2A4 antigenicity remained at 22.3% of the control; approximately 33–34-kDa allergen retained 66% of its initial reactivity	↓ Trypsin inhibitor and urease activities; ↔ isoflavone concentration	[81]
Soybean flour	7S β-Conglycinin, 11S glycinin, and trypsin inhibitor	Twin-screw extrusion; matrix temperature: 66–134 °C; 280 rpm; water addition: 12 L·h^−1^; subsequent drying and milling	↓ Antigenicity by 99.9%; approximately 0.1% residual antibody recognition	↓ Protein fractions above approximately 40 kDa; extensive alteration of the electrophoretic and immunoblot profiles	↓ Nitrogen solubility index; trypsin inhibitor outcome was antinutritional rather than direct immunological evidence	[37]
Pea flour	Legumin, predominantly the 11S fraction	100–200 °C; variable water addition and feed rate; SME > 250 kWh·t^−1^; 2–6-mm die	↓ Anti-legumin antibody reactivity under the most severe extrusion conditions	↑ Protein unfolding and cross-linking; shear-induced protein alignment	↓ SDS–DTT-soluble protein to approximately 40%; ↑ starch water-solubility index as feed moisture decreased	[85]
Pea protein isolate, low-moisture extrusion	Legumin, vicilin, convicilin, and lipoxygenase	130–170 °C; 26–35% moisture; 400–700 rpm; SME: 180–325 kJ·kg^−1^	ND	↓ α-Helix and β-sheet structures; ↑ β-turn: 9.7 → 13.3%; disappearance or aggregation of the approximately 60-kDa legumin monomer	↓ Water solubility by 50–90%; ↑ sectional expansion index to approximately 13 under selected conditions	[52]
Commercial pea protein isolates, high-moisture extrusion	Legumin, vicilin, and convicilin	40–140 °C; 60% moisture; 150 rpm; protein: water ratio of 40:60	ND; loss of electrophoretic bands was not an antibody-binding measurement	↓ Detectable legumin bands; ↔ visible vicilin and convicilin bands; ↑ inter- and intramolecular disulfide-mediated interactions.	↓ Urea-soluble protein from approximately 100% to 60%; ↔ amino acid composition	[57]
Pea protein–sunflower meal blend	Mixed pea and sunflower proteins; trypsin inhibitors	High-moisture extrusion; 125–145 °C; 48–58% moisture	ND; trypsin inhibitor activity was an antinutritional outcome	↑ Formation of anisotropic fibrous structures; protein denaturation and exposure of enzyme-accessible regions	↓ Trypsin inhibitor activity by 64%; ↓ phytic acid by 83%; ↑ in vitro protein digestibility to 86–89%	[114]
Pea, cultivar Ballet, and kidney bean, cultivar Pinto	Globulins, including vicilin and legumin; albumins	Pea: 148 °C. Kidney bean: 156 °C. Both: 100 rpm; 25% moisture; feed rate: 350 g/min	ND; SDS-PAGE and protein solubility were indirect measurements	Pea: ↓ free –SH: 21.7 → 5.5 µmol·g^−1^; ↑ disulfide-linked insoluble protein: 25.5 → 157.9 g·kg^−1^; altered 14–75-kDa bands. Kidney bean: loss of approximately 45- and 31-kDa bands; appearance of 14–22-kDa fragments	Pea: ↓ soluble protein: 760 → 220 g·kg^−1^; ↑ WHC: 1.24 → 2.86 g·g^−1^. Kidney bean: ↓ soluble protein: 730 → 140 g·kg^−1^; ↓ OAC	[84]
White wheat flour	α/β-, γ-, and ω-gliadins; glutenins	120 °C; 30% added water; 220 rpm; feed rate: 50 kg·h^−1^	↔ R5/G12-reactive gluten signal; approximately 2% decrease, not statistically significant	↓ α-Helix: 32.1 → 16.6%; ↑ intermolecular β-sheet: 26.2 → 32.8%; ↓ extractability of glutenins and selected gliadin fractions	↓ Soluble protein: 6.5 → 2.3%; slight ↓ in vitro digestibility: 95.6 → 92.7%	[15]
Baking-enzyme-enriched wheat flour, cultivar Laudias	Gliadins and glutenins	Extrusion arm of a conventional/hybrid-processing study; 40–85 °C; 400 rpm; 27% moisture; residence time < 2 min	ND	↑ Gluten-protein denaturation; ↑ compaction of the starch–protein matrix; ↓ crystallinity; formation of amorphous starch–lipid phases	↑ WaSRC by approximately 55–67%; loss of coherent dough-forming capacity	[115]

Note: ↑, increase; ↓, decrease; ↔, no significant change; →, change from the initial to the final value; ND, not determined or not directly assessed; DTT, dithiothreitol; OAC, oil absorption capacity; SME, specific mechanical energy; –SH, sulfhydryl groups; WaSRC, water-sodium carbonate solvent retention capacity; WHC, water-holding capacity. Changes detected only through SDS-PAGE, FTIR, solubility, digestibility, or protein-fraction measurements were considered indirect evidence and do not independently demonstrate reduced allergenicity. Trypsin inhibitor and urease activities were classified as antinutritional or enzymatic outcomes.

**Table 8:** Processing conditions and effects of pressure-based technologies on the immunoreactivity, structure, digestibility, and techno-functional properties of proteins from legumes and gluten-containing cereal matrices.

Food Matrix	Target Protein(s)	Processing Conditions, and Complementary Treatment	Direct Immunological Evidence	Structural or Digestive Changes	Techno-Functional, Nutritional, or Antinutritional Changes	Ref.
Whole soymilk	Kunitz soybean trypsin inhibitor, KTI/STI	HPP: 300–600 MPa; 5–25 min	± Anti-STI antibody recognition: ↓ to 44.39% of the control at 300 MPa for 5 min, but ↑ to 109% at 600 MPa	At 600 MPa: ↓ α-helix, 29.6 → 10.3%; ↑ β-sheet, 32.0 → 48.1%; fluorescence blue shift, 343 → 340 nm; ↑ aggregation	↓ Trypsin inhibitor and lipoxygenase activities; ↑ viscosity, 4.13 → 6.22 mPa·s	[23]
Soy protein isolate	7S β-Conglycinin, Gly m 5	HPP-assisted enzymatic hydrolysis: 100–600 MPa; 50 °C; 15 min; pH 8.0	↓ Antibody-reactive Gly m 5 by ≥99.5% at 400–500 MPa during the combined treatment	↑ Unfolding and exposure of enzyme-accessible sites; extensive fragmentation of α′, α, and β subunits into approximately 15–35-kDa fractions	↑ Solubility at pH 7 to 79.4% after HPP pretreatment; ↑ foaming activity, 552 → 1604% at 400 MPa	[66]
Soy protein isolate	7S β-Conglycinin, 11S glycinin, P34/Gly m Bd 30K, and lipoxygenase	HPH: 90 MPa, two passes. HPP: 350 MPa; 16 min; 20 °C; pH 6.8	HPH: ↓ antibody reactivity by 29.8%. HPP: ↓ antibody reactivity by 46.6%	HPH: ↑ α-helix, free –SH groups, and surface hydrophobicity; ↓ disordered structures. HPP: ↑ α-helical structures; ↓ β-structures and unordered regions	HPH: ↑ surface hydrophobicity by 21.79-fold. HPP: ↑ surface hydrophobicity by 23.93-fold	[60]
Soy glycinin-stabilized emulsion	11S Glycinin	UHPH: 20–350 MPa; one pass; 1–3% protein; pH 7; final temperature up to 86 °C	ND	Partial unfolding at approximately 200 MPa; disappearance of the native-protein peak and ↑ aggregation at 350 MPa	↓ Solubility, 74 → 40% at 350 MPa; transition from Newtonian liquid to pseudoplastic gel at >250 MPa	[11]
Purified soybean glycinin	Acidic and basic glycinin subunits	HPP: 100–500 MPa; 10 min; approximately 25 °C	ND	↑ Dissociation of glycinin subunits; at ≥400 MPa, ↓ ordered α-helical and β-sheet structures and ↑ random-coil structures	ND	[82]
Purified pea lectin model solution	Pea lectin	HPP: 400 MPa; 25 °C; 0 or 5 min. UST: 400 MPa; 40 °C; five cycles	ND; antibody binding was not evaluated	↔ Overall pea-lectin structure; no substantial irreversible changes or disruption of the dimeric organization detected under the reported conditions	ND	[86]
Pea protein extract	Convicilin, vicilin, legumin, and lipoxygenase	pH shifting + HPH: 150 MPa; 10 L/h; 10 °C	ND	↓ Aggregate size through shear-induced disruption; ↑ exposure of aromatic and hydrophobic regions	↑ Solubility, 36.6 → 53.6%; ↑ OHC, 5.90 → 7.88 g·g^−1^; ↑ emulsion stability	[58]
Pea globulin soluble aggregates	11S Legumin, 7S vicilin, and convicilin	Thermal aggregation + dynamic high-pressure fluidization: 90 °C for 60 min at pH 7.2, followed by 70 or 130 MPa for three passes	ND	↓ Aggregate size, 145.8 → 119.8 nm; ↓ disulfide bonds, 4.44 → 4.17 µmol·10^6^ g^−1^ protein; ↑ free –SH groups, 1.10 → 1.43 µmol·10^6^ g^−1^	↑ Surface hydrophobicity after thermal aggregation	[56]
Wheat flour	Gliadins and glutenins	HPP: 200–600 MPa; 5 min; approximately 25 °C; flour moisture: 33 or 56%	ND; gluten immunoreactivity was not evaluated	33% moisture: ↑ gluten aggregation and unextractable polymeric protein at ≥400 MPa; fibrillar networks at 500–600 MPa. 56% moisture: discontinuous gluten network and structural disruption at ≥500 MPa	At 33% moisture and 600 MPa: ↑ dough-development time, 1.82 → 3.06 min; ↑ stability, 1.70 → 3.15 min	[48]

Note: ↑, increase; ↓, decrease; ↔, no significant change; ±, variable or condition-dependent response; →, change from the initial to the final value; ND, not determined or not directly assessed; HPH, high-pressure homogenization; HPP, high-pressure processing; KTI, Kunitz trypsin inhibitor; OHC, oil-holding capacity; P34, soybean allergen Gly m Bd 30K; SAXS, small-angle X-ray scattering; –SH, sulfhydryl groups; STI, soybean trypsin inhibitor; UHPH, ultra-high-pressure homogenization; UST, ultra-shear technology.

**Table 9:** Processing conditions and effects of pulsed electric fields on the immunoreactivity, structure, digestibility, and techno-functional properties of proteins from legumes and gluten-containing cereal matrices.

Food Matrix	Target Protein(s)	Pulsed-Electric-Field Conditions	Direct Immunological Evidence	Structural or Digestive Changes	Techno-Functional Properties	Ref.
Instant soy milk powder	7S β-Conglycinin (Gly m 5) and 11S glycinin (Gly m 6)	0–40 kV·cm^−1^; 60 s; pulse width: 8 μs; 500 Hz	↓ IgE-binding ELISA signal: 1.64 ± 0.13 → 1.33 ± 0.09.↓ IgG1-binding signal: 2.26 ± 0.08 → a minimum of 1.61 ± 0.09; 1.71 ± 0.13 at 40 kV·cm^−1^	Altered tertiary conformation; ↓ α-helix and β-turn structures; modification of the accessibility or abundance of selected epitope-containing regions	↑ Susceptibility to pepsin and pancreatin digestion	[62]
Soy protein isolate	7S β-Conglycinin and 11S glycinin	0–50 kV/cm; 1.0 kHz; pulse width: 40 μs; flow rate: 10 mL·min^−1^; total effective treatment time: 4.8 ms; temperature: 25–58 °C; rapid cooling	ND	Apparent complete denaturation of 7S at 35 kV·cm^−1^ and 11S at 45 kV·cm^−1^ according to thermal analysis; at >35 kV·cm^−1^, altered antiparallel β-sheet, β-turn, and β-sheet structures; ↓ hydrogen-bond strength and denaturation enthalpy	ND	[64]
Pea protein extract	Legumin, convicilin, vicilin, and albumin fractions	24 kV/cm; 50 Hz; pulse width: 20 μs; effective treatment time: 126 μs; inlet temperature: 25–40 °C	ND	↑ Protein unfolding, indicated by a tryptophan-fluorescence red shift; ↑ parallel β-sheet: 10.6 → 13.0%; rearrangement of secondary structure	↑ Gelling capacity by 35%; ↑ mass solubility by 6%; ζ-potential became more negative: –14.1 → –22.6 mV, indicating ↑ electrostatic repulsion and colloidal stability	[53]
Vital wheat gluten	Gliadins and glutenins	Electric-field strength: 2.5–12.5 kV·cm^−1^; 100–900 Hz; pulse width: 2–10 μs; residence time: 1–9 min; selected condition reported as 5 kV·cm^−1^, 500 Hz, 6 μs, and 3 min	ND; gluten antibody reactivity and celiac-related immunogenicity were not evaluated	↓ α-helix and β-turn structures; ↑ parallel and antiparallel β-sheet structures; ↑ free –SH groups by 25.2%, reaching 3.43 μmol·g^−1^ at 12.5 kV·cm^−1^; ↓ surface hydrophobicity by 47.5% at 7.5 kV·cm^−1^; formation of smaller and more uniform pores	↑ Solubility with electric-field strength; ↓ denaturation enthalpy: 116.3 → 82.3 J·g^−1^	[116]

Note: ↑, increase; ↓, decrease; →, change from the initial to the final value; ND, not determined or not directly assessed; PEF, pulsed electric fields; –SH, sulfhydryl groups; ζ-potential, zeta potential.

**Table 10:** Processing conditions and effects of other processing strategies and combined treatments on the immunoreactivity, structure, digestibility, and techno-functional properties of proteins from legumes and gluten-containing cereal matrices.

Food Matrix	Target Protein(s)	Technology, Processing Conditions, and Complementary Treatment	Direct Immunological Evidence	Structural or Digestive Changes	Techno-Functional Properties	Ref.
Wet pea protein extract	Convicilin, vicilin, legumin, albumins, and lipoxygenase	pH shifting: pH 12.0; 50 °C; 1 h. pH shifting + HPH: pH 12.0 for 1 h at 50 °C, followed by 150 MPa; 10 L·h^−1^; 10 °C	ND; antibody binding or allergen-specific immunoreactivity was not evaluated	pH shifting: ↔ primary and secondary protein profiles by SDS-PAGE and FTIR; ↓ aggregate size to ~20 nm; ↑ free –SH groups to 17.38 μmol·g^−1^; ↑ exposure of hydrophilic and hydrophobic regions. pH shifting + HPH: partial reassociation of small particles to ~40 nm; ↑ intramolecular interactions	pH shifting:↑ solubility by a reported 76%. pH shifting + HPH: ↑ emulsion stability; ↑ interfacial properties, WHC, OHC, and antioxidant activity	[58]
Mung bean protein isolate	Vig r 2, Vig r 4, and β-conglycinin-like protein	pH shifting: pH 2.0 or 12.0; 25 °C; 1 h. pH shifting + ultrasound: pH 2.0 or 12.0 followed by ultrasound at 700 W and 30 kHz; 15 min; 30-s on/off pulses; approximately 20 °C	ND; SDS-PAGE, LC–MS/MS, and AllerCatPro 2.0 did not directly assess IgE or IgG binding	Alkaline pH shifting: ↓ abundance or analytical detection of Vig r 4 and β-conglycinin-like protein; ↑ free –SH groups and exposure of hydrophobic regions. pH shifting + ultrasound: ↓ particle size: 5300 → 174.6 nm; ↑ negative surface charge: −21.3 → −26.3 mV; conformational shift from a β-sheet-rich structure toward ↑ α-helix content, reaching 72.0%	pH shifting + ultrasound:↑ solubility: 45.9 → 91.2%; modified protein–gellan gum interactions and gel-forming behavior	[88]
Extruded toasted soybean meal	11S Glycinin, including acidic and basic subunits; 7S β-conglycinin, including α′, α, and β subunits	Extrusion pretreatment + enzymatic hydrolysis:substrate concentration/enzyme dose: 26%/0.4, 26.9%/0.9, 29%/2.0, 31.1%/3.1, and 32%/3.6 PU·g^−1^; 50 °C; pH 6.1; 5 h	ND; disappearance of SDS-PAGE bands was not an antibody-binding measurement	↑ DH to a reported maximum of 42.1%; ↓ detectable intact glycinin and β-conglycinin subunits; formation of low-molecular-weight peptides; no bands >15 kDa after extrusion at 120–130 °C followed by hydrolysis	↑ Maximum reported solubility to 42.98%; ↑ dynamic viscosity: 0.20 → 5.82 Pa·s as substrate concentration increased	[70]
Vital wheat gluten	Gliadins and glutenins	Heat pretreatment + enzymatic hydrolysis: Alcalase 2.4 L from *Bacillus licheniformis*; 60 °C; pH 8.0 adjusted with 0.8 M NaOH; total hydrolysis time: 195 min; DH: 16.1, 22.9, and 30.2%	ND; IgE binding, R5/G12 recognition, and celiac-related immunogenicity were not evaluated	↑ Proteolysis with increasing DH; generation of lower-molecular-weight gluten hydrolysates; structural changes resulted from the combined effects of thermal pretreatment and Alcalase hydrolysis	↑ Solubility across pH 2–12; ↑ emulsifying activity and emulsion stability at higher DH; ↓ foaming capacity and foam stability at higher DH	[41]
Purified soybean 7S protein	α′, α, and β subunits of 7S β-conglycinin	Punicalagin binding + ultrasound: 7S:PUN molar ratios of 1:10–1:100; binding at 25 °C for 80 min; ultrasound at 100–500 W for 10 min; subsequent dialysis	↓ IgG-binding signal by 4.78–58.05%, depending on the PUN concentration; reduced recognition of selected IgG-reactive regions. IgE binding was not evaluated	↑ Formation of stable protein–punicalagin complexes; altered secondary structure and intrinsic fluorescence; ↑ exposure of hydrophobic regions	ND	[22]
Soybean protein system	7S β-Conglycinin, 11S glycinin, and Kunitz trypsin inhibitor (Gly m TI)	Ohmic heating: 2–20 V·cm^−1^; 50 Hz–20 kHz; 95 °C; 10 min	↓ Gly m TI immunoreactivity by approximately 36% at 50 Hz; response was frequency-dependent	Altered conformation and aggregation; ↓ intrinsic fluorescence by approximately 27% at 50 Hz; metal–protein interactions were reported; ↔ exposed hydrophobic regions	ND	[68]
Purified soybean glycinin dispersion	11S Glycinin	Hydrodynamic cavitation: 550-W pump; 0–30 min; pH 2–10; protein concentration: 3 g·100 mL^−1^; 3-mm orifice; temperature controlled by cooling	ND	At pH 6: ↓ particle size, 459.57 → 322.97 nm; ↑ surface hydrophobicity, 850.95 → 988.43; ↓ α-helix and ↑ β-sheet structures; ↓ aggregation	At pH 6: ↑ solubility, 21.85 → 63.62 g·100 g^−1^; ↑ ESI, 58.39 → 127.74 min; ↓ EAI; ↑ absolute ζ-potential	[78]
Soy protein isolate	7S and 11S globulins	Electrolysis: direct current; platinum electrodes and Nafion membrane; 30–180 min; 25 °C; pH 7.0; protein concentration: 0.04 g·mL^−1^	ND	Time-dependent rearrangement of disulfide-bond conformations; ± α-helix: 29.11 → 15.60% at 120 min and 36.29% at 150 min; ↓ β-sheet to 24.59%; ↑ random coil: 21.97 → 37.44%; ↑ exposure of Trp and Tyr residues	ND	[73]
Mung bean protein isolate–dextran system	8S Vicilin-like and 11S legumin-like globulins	Controlled Maillard reaction: protein–dextran system; 80 or 90 °C; pH 7.0; 0–6 h; subsequent freeze-drying	ND; SDS-PAGE band attenuation was not an antibody-binding measurement	↓ Intensity of 60-, 43–48-, 32-, 26-, and 21-kDa bands; disappearance of 32- and 26-kDa bands after ≥5 h at 80 °C; ↓ α-helix and ↑ β-sheet structures; fluorescence red shift	↑ Solubility compared with untreated protein, particularly at 80 °C; solubility subsequently decreased during prolonged treatment because of aggregation	[87]
White lupin	β-Conglutin, including Lup an 1; δ- and γ-conglutins; legumin-like proteins	Lactic fermentation and thermal processing: fermentation at 43 °C for 24 h in a whey-based system; thermal treatment at 100 °C for 30 min evaluated as an additional processing step	ND; allergen-specific antibody binding was not evaluated	↓ Detectable intact β-conglutin; ↑ diversity of peptides after INFOGEST digestion; generation of processing-specific peptide sequences, including 8-mer peptides	ND	[92]
Hydrated vital wheat gluten and gluten powder	α/β-, γ-, and ω-gliadins; glutenins	Pulsed light: fluence of 1.75–26.25 J·cm^−2^; xenon lamps, 200–1000 nm; pulse duration: 0.50 μs; 0.5 Hz; lamp distance: 1–3 cm; temperature ≤ 45 °C	Gluten suspension: ↓ R5-reactive gliadin signal by approximately 50% at 26.25 J/cm^2^. Gluten powder: smaller reduction than in the hydrated system	↑ Partial depolymerization of hydrated gluten; ↑ free –SH groups; disulfide-exchange reactions; formation of new protein species; oxidation and nonenzymatic browning	↑ Browning, representing a potential adverse quality change	[24]
Vital wheat gluten	Gliadins; HMW- and LMW-glutenin subunits	Planetary ball milling: 400 rpm; dry milling for 0–40 min; aluminum oxide jars; alternating operation every 5 min	ND	At 20 min: ↓ α-helix, 22.70 → 17.44%; ↓ β-sheet, 27.36 → 21.07%; ↑ β-turn, 28.94 → 45.38%; ↑ free –SH groups, 14.1 → 24.1 μmol·g^−1^. At 40 min: ↓ particle size, 85.9 → 32.3 μm	↑ Surface hydrophobicity, 959.72 → 1194.33; ↑ EAI, 0.18 → 0.27 m^2^/g; ↑ FC, 8.7 → 31.0 cm^3^; ↑ FS, 76.8 → 94.2%; ↑ whiteness	[46]
Whole peanut kernels	Ara h 1, Ara h 2, Ara h 3, and Ara h 4	Pulsed light: 5–9.5 min; lamp distance: 7 or 10 cm; 3 pulses·s^−1^; approximately 1.27 J·cm^−2^ per lamp; emission comprising UV, visible, and infrared radiation	↓ IgE-binding ELISA signal by up to 80%; marked reduction in antibody recognition of Ara h1, Ara h2, Ara h3, Ara h4	↓ Intact allergen bands; fragmentation into polypeptides <17 kDa after >6 min; formation of insoluble aggregates; photo-oxidation, denaturation, and protein cross-linking	ND	[90]

Note: ↑, increase; ↓, decrease; ↔, no significant change; ±, variable or treatment-dependent response; →, change from the initial to the final value; DH, degree of hydrolysis; EAI, emulsifying activity index; ESI, emulsion stability index; FC, foaming capacity; FS, foam stability; HMW, high molecular weight; HPH, high-pressure homogenization; LMW, low molecular weight; ND, not determined or not directly assessed; OHC, oil-holding capacity; PU, protease units; PUN, punicalagin; –SH, sulfhydryl groups; WHC, water-holding capacity; ζ-potential, zeta potential.

## Data Availability

No new data were created or analyzed in this study. Data sharing is not applicable to this article.

## Supplementary Materials

The following supporting information can be downloaded at: https://www.mdpi.com/article/10.3390/molecules31193438/s1, Table S1: Bibliographic characteristics of the studies included in the systematic review; Table S2: Evidence-extraction matrix of food matrices, allergenic proteins, emerging processing conditions, analytical methods, structural modifications, allergenicity outcomes, and limitations of the included studies; and the PRISMA 2020 checklist. Reference [117] is cited in the Supplementary Materials.

## Abbreviations

The following abbreviations are used in this manuscript:

BANA	Nα-benzoyl-DL-arginine-β-naphthylamide
DBD	Dielectric barrier discharge
DH	Degree of hydrolysis
EAI	Emulsifying activity index
ELISA	Enzyme-linked immunosorbent assay
ESI	Emulsion stability index
FC	Foaming capacity
FS	Foam stability
FTIR	Fourier-transform infrared spectroscopy
HMW	High molecular weight
HPH	High-pressure homogenization
HPP	High-pressure processing
IgE	Immunoglobulin E
IgG	Immunoglobulin G
IR	Infrared
KTI	Kunitz trypsin inhibitor
LC–MS/MS	Liquid chromatography–tandem mass spectrometry
LMW	Low molecular weight
OHC	Oil-holding capacity
PBS	Phosphate-buffered saline
PDI	Protein dispersibility index
PEF	Pulsed electric fields
PIO	Population, Intervention, and Outcomes
PRISMA	Preferred Reporting Items for Systematic Reviews and Meta-Analyses
RONS	Reactive oxygen and nitrogen species
RP-HPLC	Reversed-phase high-performance liquid chromatography
SAXS	Small-angle X-ray scattering
SDS-PAGE	Sodium dodecyl sulfate–polyacrylamide gel electrophoresis
SEC-HPLC	Size-exclusion high-performance liquid chromatography
SME	Specific mechanical energy
STI	Soybean trypsin inhibitor
UHPH	Ultra-high-pressure homogenization
US	Ultrasound
UST	Ultra-shear technology
UV	Ultraviolet
WHC	Water-holding capacity

## References

[1] Firoozzare A., Boccia F., Yousefian N., Ghazanfari S., Pakook S. (2024). Understanding the role of awareness and trust in consumer purchase decisions for healthy food and products. Food Qual. Prefer..

[2] Huangfu P., Pearson F., Abu-Hijleh F.M., Wahlich C., Willis K., Awad S.F., Abu-Raddad L.J., Critchley J.A. (2024). Impact of price reductions, subsidies, or financial incentives on healthy food purchases and consumption: A systematic review and meta-analysis. Lancet Planet. Health.

[3] Diego T., Fiorella S., Joaquín A., Vanina N., Nicolás P., Mónica W. (2024). Dietas saludables y sustentables como eje transformador de los modos actuales de producción de alimentos: Evidencias desde la demanda actual en la ciudad de Río Cuarto, Córdoba, Argentina. SaberEs.

[4] Licandro H., Ho P.H., Nguyen T.K.C., Petchkongkaew A., Nguyen H.V., Chu-Ky S., Nguyen T.V.A., Lorn D., Waché Y. (2020). How fermentation by lactic acid bacteria can address safety issues in legumes food products?. Food Control.

[5] Lisciani S., Marconi S., Le Donne C., Camilli E., Aguzzi A., Gabrielli P., Gambelli L., Kunert K., Marais D., Vorster B.J. (2024). Legumes and common beans in sustainable diets: Nutritional quality, environmental benefits, spread and use in food preparations. Front. Nutr..

[6] Zhang W., Boateng I.D., Xu J., Zhang Y. (2024). Proteins from Legumes, Cereals, and Pseudo-Cereals: Composition, Modification, Bioactivities, and Applications. Foods.

[7] Benjamin J., Idowu O., Babalola O.K., Oziegbe E.V., Oyedokun D.O., Akinyemi A.M., Adebayo A. (2024). Cereal production in Africa: The threat of certain pests and weeds in a changing climate—A review. Agric. Food Secur..

[8] Nguyen S.N., Drawbridge P., Beta T. (2024). Distribution of cereal phytochemicals and micronutrients in whole grains: A review of nutraceutical, industrial, and agricultural implications. Cereal Chem..

[9] Skeie G., Fadnes L.T. (2024). Cereals and cereal products—A scoping review for Nordic Nutrition Recommendations 2023. Food Nutr. Res..

[10] Li J., Zhang X., Lian H., Zhang C., Zheng H., Han D., Dou H. (2025). Asymmetrical flow field-flow fractionation coupled with multi-detector: A robust method for evaluating the degradation of jujube polysaccharides with ultrasound-assisted H_2_O_2_ and subsequent conjugation with soybean 7S globulin. Food Chem..

[11] Floury J., Desrumaux A., Legrand J. (2002). Effect of Ultra-high-pressure Homogenization on Structure and on Rheological Properties of Soy Protein-stabilized Emulsions. J. Food Sci..

[12] Abdelwhab N.M., Nour A.A.A.M., Fageer A.S.M. (2009). The nutritive and functional properties of dry bean (*Phaseolus vulgaris*) as affected by gamma irradiation. Pak. J. Nutr..

[13] Xing G., Yang S., Huang L., Liu S., Wan X. (2024). Effect of transglutaminase crosslinking combined with lactic fermentation on the potential allergenicity and conformational structure of soy protein. J. Sci. Food Agric..

[14] Zhang L., Gong S., Zuo Y., Zhang L., Chen J., Xu Y., Wu Y., Zhao Y., Wu J.-L., Li N. (2025). Soybean fermentation drives the production of native neuroprotective peptides based on a peptidomics strategy. Curr. Res. Food Sci..

[15] Mattioni B., Tilley M., Scheuer P.M., Paulino N., Yucel U., Wang D., de Francisco A. (2024). Flour Treatments Affect Gluten Protein Extractability, Secondary Structure, and Antibody Reactivity. Foods.

[16] Sharma P., Malhotra L., Dhamija R.K. (2025). Comprehensive amino acid composition analysis of seed storage proteins of cereals and legumes: Identification and understanding of intrinsically disordered and allergenic peptides. J. Biomol. Struct. Dyn..

[17] Das D., Kabir M.E., Borah A., Phukan T., Kaita J., Manna P. (2024). Alpha-glucosidase inhibitory and glucoregulatory potential of soy protein fermented with a bacterium (*Bacillus subtilis* PM_NEIST_3) isolated from Hawaijar. Food Biosci..

[18] Pi X., Zhu L., Liu J., Zhang B. (2024). Effect of Thermal Processing on Food Allergenicity: Mechanisms, Application, Influence Factor, and Future Perspective. J. Agric. Food Chem..

[19] Davis P.J., Smales C.M., James D.C. (2001). How can thermal processing modify the antigenicity of proteins?. Allergy.

[20] Jiménez-Saiz R., Benedé S., Molina E., López-Expósito I. (2015). Effect of Processing Technologies on the Allergenicity of Food Products. Crit. Rev. Food Sci. Nutr..

[21] Shriver S.K., Yang W.W. (2011). Thermal and Nonthermal Methods for Food Allergen Control. Food Eng. Rev..

[22] Xu Y., Shi J., Wang X., Wang Y., Zhao Y., Raghavan V., Wang J. (2025). Effects of ultrasound-assisted punicalagin binding on the allergenicity reduction of soybean protein 7S. Food Chem..

[23] Rajan A., Velusamy M., Baskaran K., Rangarajan J., Natarajan V., Radhakrishnan M. (2023). High pressure processing of whole soymilk: Effect on allergenicity, anti-nutritional factor, lipoxygenase activity and E-nose-aroma characteristics. Food Chem. Adv..

[24] Panozzo A., Manzocco L., Lippe G., Nicoli M.C. (2016). Effect of pulsed light on structure and immunoreactivity of gluten. Food Chem..

[25] Kasera R., Singh A.B., Kumar R., Lavasa S., Prasad K.N., Arora N. (2012). Effect of thermal processing and γ-irradiation on allergenicity of legume proteins. Food Chem. Toxicol..

[26] Alhendi A.S., Alobaidy Z.S. (2023). Effect of ultraviolet irradiation on immunoreactivity and other protein characteristics of wheat gliadin in flour and flatbread. Food Sci. Appl. Biotechnol..

[27] Tahmasian A., Drew R., Broadbent J.A., Juhász A., Nye-Wood M., Colgrave M.L. (2023). Conventional solid-state fermentation impacts the white lupin proteome reducing the abundance of allergenic peptides. Food Chem..

[28] Mousavi Khaneghah A., Nematollahi A., AbdiMoghadam Z., Davoudi M., Mostashari P., Marszałek K., Aliyeva A., Javanmardi F. (2024). Research progress in the application of emerging technology for reducing food allergens as a global health concern: A systematic review. Crit. Rev. Food Sci. Nutr..

[29] Lyu H., Li X., Zhang X., Zeng L., Zhu Q. (2025). Legume allergy: Binding epitopes, detection methods, mitigation techniques, diagnosis and immunotherapy strategies. Food Sci. Hum. Wellness.

[30] Page M.J., McKenzie J.E., Bossuyt P.M., Boutron I., Hoffmann T.C., Mulrow C.D., Shamseer L., Tetzlaff J.M., Akl E.A., Brennan S.E. (2021). Declaración PRISMA 2020: Una guía actualizada para la publicación de revisiones sistemáticas. Rev. Esp. Cardiol..

[31] Vaz A.F.M., Souza M.P., Carneiro-Da-Cunha M.G., Medeiros P.L., Melo A.M.M.A., Aguiar J.S., Silva T.G., Silva-Lucca R.A., Oliva M.L.V., Correia M.T.S. (2012). Molecular fragmentation of wheat-germ agglutinin induced by food irradiation reduces its allergenicity in sensitised mice. Food Chem..

[32] Kumar V., Rani A., Hussain L., Yadav M., Jha P., Petwal V., Dwivedi J. (2017). Changes in physico-chemical properties of native and toasted defatted soy flour on submission to electron beam radiation. Food Bioprod. Process..

[33] Liu M., Zhao G., Qi B., Zhan J. (2016). Effects of ultrasonic treatment on removal of abundant proteins and enrichment of low-abundance proteins in defatted soybean meal by isopropanol. Biotechnol. Biotechnol. Equip..

[34] Liu M., Qi B., Liu Z., Zhan J., Zhan K., Zhao G. (2017). Optimization of low-abundance protein extraction and abundant protein removal from defatted soybean meal. J. Zhejiang Univ. Sci. B.

[35] Mallikarjunan N., Marathe S., Deshpande R., Jamdar S.N., Sharma A. (2012). Influence of γ-radiation on the structure and function of soybean trypsin inhibitor. J. Agric. Food Chem..

[36] Moriyama T., Yano E., Kitta K., Kawamoto S.-I., Kawamura Y., Todoriki S. (2013). Effect of gamma irradiation on soybean allergen levels. Biosci. Biotechnol. Biochem..

[37] Ohishi A., Watanabe K., Urushibata M., Utsuno K., Ikuta K., Sugimoto K., Harada H. (1994). Detection of soybean antigenicity and reduction by twin-screw extrusion. J. Am. Oil Chem. Soc..

[38] Olakanmi S., Barbhuiya R.I., Wroblewski C., Ramalingam S., Wang J., Nair G.R., Singh A. (2023). Effect of microwave and conventional heat treatment on trypsin inhibitor activity and in vitro digestibility of edamame milk protein. Food Bioeng..

[39] Yang H., Gao J., Yang A., Chen H. (2015). The ultrasound-treated soybean seeds improve edibility and nutritional quality of soybean sprouts. Food Res. Int..

[40] Aly A.A., Maraei R.W., Baraket M. (2019). Effects of gamma irradiation and salt stress on amino acids and protein fractions of two egyptian bread wheat (*Triticum aestivum* L.) cultivars. Bangladesh J. Bot..

[41] Elmalimadi M.B., Stefanović A.B., Šekuljica N.Ž., Žuža M.G., Luković N.D., Jovanović J.R., Knežević-Jugović Z.D. (2017). The synergistic effect of heat treatment on alcalase-assisted hydrolysis of wheat gluten proteins: Functional and antioxidant properties. J. Food Process. Preserv..

[42] Guo Z., Zhang H., Chen K., Wang Z., Chen G., Yang B., Kan J. (2023). Characterization of sonicated gluten protein and subsequent rheological properties of model dough and noodles. J. Sci. Food Agric..

[43] Jin H.-L., Wang J.-S., Bian K. (2012). Characteristics of enzymatic hydrolysis of the wheat gluten proteins treated by ultrasound wave. Adv. Mater. Res..

[44] Köksel H., Sapirstein H.D., Çelik S., Bushuk W. (1998). Effects of gamma-irradiation of wheat on gluten proteins. J. Cereal Sci..

[45] Li Y., Zhang Y., Liu X., Wang H., Zhang H. (2019). Effect of ultrasound-assisted freezing on the textural characteristics of dough and the structural characterization of wheat gluten. J. Food Sci. Technol..

[46] Liu Z., Zheng Z., Zhu G., Luo S., Zhang D., Liu F., Shen Y. (2021). Modification of the structural and functional properties of wheat gluten protein using a planetary ball mill. Food Chem..

[47] Mahanta S., Castell-Perez E., Awika J.M., Moore J.M. (2026). Atmospheric Cold Plasma Treatment of Hard Wheat Flour and Soft Wheat Flour: Effects on Thiol Group Oxidation and Dough Rheology. Food Bioprocess Technol..

[48] McCann T.H., Leder A., Buckow R., Day L. (2013). Modification of structure and mixing properties of wheat flour through high-pressure processing. Food Res. Int..

[49] Yalcin E., Sakiyan O., Sumnu G., Celik S., Koksel H. (2008). Functional properties of microwave-treated wheat gluten. Eur. Food Res. Technol..

[50] Zadeike D., Zvirdauskiene R., Basinskiene L. (2025). Gluten Functionality Modification: The Effect of Enzymes and Ultrasound on the Structure of the Gliadin–Glutenin Complex and Gelling Properties. Molecules.

[51] Zhang H., Claver I.P., Zhu K.-X., Zhou H. (2011). The effect of ultrasound on the functional properties of wheat gluten. Molecules.

[52] Beck S.M., Knoerzer K., Arcot J. (2017). Effect of low moisture extrusion on a pea protein isolate’s expansion, solubility, molecular weight distribution and secondary structure as determined by Fourier Transform Infrared Spectroscopy (FTIR). J. Food Eng..

[53] De Gol C., De Ridder V., Zwietering M.H., den Besten H.M.W., Beyrer M. (2025). Effects of pulsed electric field and thermal treatment on the structural and techno-functional properties of mildly extracted pea proteins. LWT.

[54] Heydari M.M., Movasaghi M., Higa F., Nickerson M., Meda V., Zhang L. (2026). Cold plasma jet-induced modifications in pea protein: A comparative study of gas-specific effects. Innov. Food Sci. Emerg. Technol..

[55] Mahdavian Mehr H., Koocheki A. (2020). Effect of atmospheric cold plasma on structure, interfacial and emulsifying properties of Grass pea (*Lathyrus sativus* L.) protein isolate. Food Hydrocoll..

[56] Oliete B., Potin F., Cases E., Saurel R. (2018). Modulation of the emulsifying properties of pea globulin soluble aggregates by dynamic high-pressure fluidization. Innov. Food Sci. Emerg. Technol..

[57] Osen R., Toelstede S., Eisner P., Schweiggert-Weisz U. (2015). Effect of high moisture extrusion cooking on protein-protein interactions of pea (*Pisum sativum* L.) protein isolates. Int. J. Food Sci. Technol..

[58] Plazzotta S., Melchior S., Barozzi L., Saitta A., Nicoli M.C., Manzocco L. (2026). Effect of pH shift and high pressure homogenization on the structure and techno-functional properties of protein extracts from substandard peas. Curr. Res. Food Sci..

[59] Ravindran A., Ramaswamy H.S. (2023). Effect of sonication—Cooking on the immunoreactivity of soy slurry from germinated soybeans. Food Innov. Adv..

[60] Li H., Zhu K., Zhou H., Peng W., Guo X. (2016). Comparative study of four physical approaches about allergenicity of soybean protein isolate for infant formula. Food Agric. Immunol..

[61] Amponsah A., Nayak B. (2016). Effects of Microwave and Ultrasound Assisted Extraction on the Recovery of Soy Proteins for Soy Allergen Detection. J. Food Sci..

[62] Ding J., Li Y., Zhong L., Lu X., Bao Z., Lin S. (2025). Unveiling the regulatory mechanism of pulsed electric field modified instant soy milk powder: Exploring allergenicity, conformation, and epitopes. Food Chem..

[63] Lee K.J., Kim J.-B., Kim S.H., Ha B.-K., Lee B.-M., Kang S.-Y., Kim D.S. (2011). Alteration of seed storage protein composition in soybean [*Glycine max* (L.) Merrill] mutant lines induced by γ-irradiation mutagenesis. J. Agric. Food Chem..

[64] Liu Y.Y., Zeng X.A., Deng Z., Yu S.J., Yamasaki S. (2011). Effect of pulsed electric field on the secondary structure and thermal properties of soy protein isolate. Eur. Food Res. Technol..

[65] Manjaya J.G., Suseelan K.N., Gopalakrishna T., Pawar S.E., Bapat V.A. (2007). Radiation induced variability of seed storage proteins in soybean [*Glycine max* (L.) Merrill]. Food Chem..

[66] Meinlschmidt P., Brode V., Sevenich R., Ueberham E., Schweiggert-Weisz U., Lehmann J., Rauh C., Knorr D., Eisner P. (2017). High pressure processing assisted enzymatic hydrolysis—An innovative approach for the reduction of soy immunoreactivity. Innov. Food Sci. Emerg. Technol..

[67] Moscoso Ospina Y.A., Porfiri M.C., Cabezas D.M. (2022). Soybean okara: Effect of ultrasound on compositional and emulsifying properties. Int. J. Food Sci. Technol..

[68] Pereira R.N., Rodrigues R.M., Machado L., Ferreira S., Costa J., Villa C., Barreiros M.P., Mafra I., Teixeira J.A., Vicente A.A. (2021). Influence of ohmic heating on the structural and immunoreactive properties of soybean proteins. LWT.

[69] Rahman M.M., Byanju B., Grewell D., Lamsal B.P. (2020). High-power sonication of soy proteins: Hydroxyl radicals and their effects on protein structure. Ultrason. Sonochem..

[70] Sharikov A.Y., Sereda A.S., Kostyleva E.V., Velikoretskaya I.A., Polyakov V.A. (2016). Enzymatic hydrolysis of extruded soybean meal at high substrate concentrations. Acta Period. Technol..

[71] Taghinejad M., Nikkhah A., Sadeghi A.A., Raisali G., Chamani M. (2009). Effects of gamma irradiation on chemical composition, antinutritional factors, ruminal degradation and in vitro protein digestibility of full-fat soybean. Asian-Aust. J. Anim..

[72] Wang Y., Wang Z., Handa C.L., Xu J. (2017). Effects of ultrasound pre-treatment on the structure of β-conglycinin and glycinin and the antioxidant activity of their hydrolysates. Food Chem..

[73] Yu D., Zhang X., Zou W., Tang H., Yang F., Wang L., Elfalleh W. (2021). Raman spectroscopy analysis of the effect of electrolysis treatment on the structure of soy protein isolate. J. Food Meas. Charact..

[74] Zhang J., Cui T., Zhang L., Xu H., Xu J., Wang J. (2025). Impact of Microwave Time on the Structure and Functional Properties of Glycosylated Soy 7S Globulins. Foods.

[75] Zhang Q., Cheng Z., Zhang J., Nasiru M.M., Wang Y., Fu L. (2021). Atmospheric cold plasma treatment of soybean protein isolate: Insights into the structural, physicochemical, and allergenic characteristics. J. Food Sci..

[76] Zhou Y., Liu L., Zhang L., Tan Y., Li X., Peng K., Liu Z., Gong D. (2022). The effects of dielectric barrier discharge plasma treatment on the structure and gelling properties of soy protein isolate. J. Henan Univ. Technol. Nat. Sci. Ed..

[77] Afify A.E.-M.M.R., Rashed M.M., Mahmoud E.A., El-Beltagi H. (2011). Effect of gamma radiation on protein profile, protein fraction and solubility’s of three oil seeds: Soybean, peanut and sesame. Not. Bot. Horti Agrobot. Cluj-Napoca.

[78] Hou Y., Yang F., Cao J., Huang Y., Li C., Li J., Ren X. (2022). Effects of hydrodynamic cavitation at different pH values on the physicochemical properties and aggregation behavior of soybean glycinin. LWT.

[79] Hu H., Cheung I.W.Y., Pan S., Li-Chan E.C.Y. (2015). Effect of high intensity ultrasound on physicochemical and functional properties of aggregated soybean β-conglycinin and glycinin. Food Hydrocoll..

[80] Liu Z.-W., Zhou Y.-X., Wang F., Tan Y.-C., Cheng J.-H., Bekhit A.E.-D., Aadil R.M., Liu X.-B. (2021). Oxidation induced by dielectric barrier discharge (DBD) plasma treatment reduces IgG/IgE binding capacity and improves the functionality of glycinin. Food Chem..

[81] Saitoh S., Urushibata M., Ikuta K., Fujimaki A., Harada H. (2000). Antigenicity in soybean hypocotyls and its reduction by twin-screw extrusion. JAOCS J. Am. Oil Chem. Soc..

[82] Zhang H., Li L., Tatsumi E., Kotwal S. (2003). Influence of high pressure on conformational changes of soybean glycinin. Innov. Food Sci. Emerg. Technol..

[83] Wang H., Tong X., Zhong M., Qi B., Li Y., Jiang L. (2018). Effect of Microwave Drying on Protein Subunits and Functional Properties of Enzyme-assisted Aqueous Extraction Processing Protein Rich Bean Powder. Nongye Jixie XuebaoTransactions Chin. Soc. Agric. Mach..

[84] Alonso R., Orúe E., Zabalza M.J., Grant G., Marzo F. (2000). Effect of extrusion cooking on structure and functional properties of pea and kidney bean proteins. J. Sci. Food Agric..

[85] Valle G.D., Quillien L., Gueguen J. (1994). Relationships between processing conditions and starch and protein modifications during extrusion-cooking of pea flour. J. Sci. Food Agric..

[86] Janahar J.J., Hu H., Balasubramaniam V.M., Teixeira S.C.M. (2025). Small Angle X-Ray Scattering Studies on the Effects of Ultra Shear Technology on Model Animal and Plant-Based Protein Solutions. J. Food Process Eng..

[87] Zhou L., Wu F., Zhang X., Wang Z. (2017). Structural and functional properties of Maillard reaction products of protein isolate (mung bean, *Vigna radiate* (L.)) with dextran. Int. J. Food Prop..

[88] Taheri A., Tan C.F., Bi X., Ong F., Binte Abdul Halim F.N., Du J. (2025). Effects of pH-shifting and ultrasound on the allergenicity and physicochemical properties of mung bean protein: Application in gellan gum-based complex gels. Food Chem..

[89] Venkataratnam H., Sarangapani C., Cahill O., Ryan C.B. (2019). Effect of cold plasma treatment on the antigenicity of peanut allergen Ara h 1. Innov. Food Sci. Emerg. Technol..

[90] Zhao X., Yang W., Chung S.-Y., Sims C.A., Otwell S.W., Rababah T.M. (2014). Reduction of IgE Immunoreactivity of Whole Peanut (*Arachis hypogaea* L.) After Pulsed Light Illumination. Food Bioprocess Technol..

[91] Rababah T., Albiss B., Aludatt M., Akkam Y., Abu Kayed A. (2021). Effect of Ultrasound Treatment on the Physicochemical, Nutraceutical, and Functional Properties of Lupine Flour. J. Agric. Sci. Technol..

[92] Ozyurek-Arpa F., Yaman M., Çakır B., Kalkan I. (2026). Impact of thermal and biotechnological processing on the bioaccessibility and allergenic peptide profile of white lupin (*Lupinus albus*). Front. Nutr..

[93] González-Osuna M.F., Torres-Arreola W., Márquez-Ríos E., Wong-Corral F.J., Lugo-Cervantes E., Rodríguez-Figueroa J.C., García-Sánchez G., Ezquerra-Brauer J.M., Soto-Valdez H., Castillo A. (2023). Antioxidant Activity of Peptide Fractions from Chickpea Globulin Obtained by Pulsed Ultrasound Pretreatment. Horticulturae.

[94] Miranda C.G., Speranza P., Kurozawa L.E., Kawazoe Sato A.C. (2022). Lentil protein: Impact of different extraction methods on structural and functional properties. Heliyon.

[95] Thalía Flores-Jiménez N., Armando Ulloa J., Esmeralda Urías-Silvas J., Carmen Ramírez-Ramírez J., Ulises Bautista-Rosales P., Gutiérrez-Leyva R. (2022). Influence of high-intensity ultrasound on physicochemical and functional properties of a guamuchil *Pithecellobium dulce* (Roxb.) seed protein isolate. Ultrason. Sonochem..

[96] Cao J., Zhu H., Wang J., Meng T., Zhang G. (2023). Effects of high-intensity ultrasonic bath on the structure and antigenicity of gliadin. J. Henan Univ. Technol. Nat. Sci. Ed..

[97] Kwak J.-H., Kim K.-B.-W.-R., Lee C.-J., Kim M.-J., Kim D.-H., Sunwoo C., Jung S.-A., Kang J.-Y., Kim H.-J., Choi J.-S. (2011). Changes in antigenicity of gliadin from medium flour by autoclave and microwave treatments. J. Korean Soc. Food Sci. Nutr..

[98] Mahroug H., Ribeiro M., Rhazi L., Bentallah L., Zidoune M.N., Nunes F.M., Igrejas G. (2019). How microwave treatment of gluten affects its toxicity for celiac patients? A study on the effect of microwaves on the structure, conformation, functionality and immunogenicity of gluten. Food Chem..

[99] Kozub N.A., Sozinov I.A., Blume Y.B., Sozinov A.A. (2013). Study of the effects produced by gamma-irradiation of common wheat F 1 seeds using gliadins as genetic markers. Cytol. Genet..

[100] Kumar R.R., Hasija S., Goswami S., Tasleem M., Sakhare A., Kumar S., Bakshi S., Jambhulkar S., Rai G.K., Singh B. (2019). Gamma irradiation protect the developing wheat endosperm from oxidative damage by balancing the trade-off between the defence network and grains quality. Ecotoxicol. Environ. Saf..

[101] Leszczynska J., Ła̧cka A., Szemraj J., Lukamowicz J., Zegota H. (2003). The influence of gamma irradiation on the immunoreactivity of gliadin and wheat flour. Eur. Food Res. Technol..

[102] Warchalewski J.R., Gralik J. (2010). Influence of microwave heating on biological activities and electrophoretic pattern of albumin fraction of wheat grain. Cereal Chem..

[103] Teng Y., Ou M., Wu J., Jiang T., Zheng K., Guo Y., Pan D., Zhang T., Wu Z. (2025). Emulsifying Properties of Oat Protein/Casein Complex Prepared Using Atmospheric Cold Plasma with pH Shifting. Foods.

[104] Wang S., Li M., Dong G., Shen R., Dong J., Li Y. (2025). Enhancement of Pea–Oat Composite Protein Gel Properties Through Ultrasound Treatment Affects Structural and Functional Characteristics. Foods.

[105] Leszczynska J., Ła̧cka A., Szemraj J., Lukamowicz J., Zegota H. (2003). The effect of microwave treatment on the immunoreactivity of gliadin and wheat flour. Eur. Food Res. Technol..

[106] Gazikalović I., Mijalković J., Šekuljica N., Tanasković S.J., Vuković A.Ð., Mojović L., Knežević-Jugović Z. (2021). Synergistic effect of enzyme hydrolysis and microwave reactor pretreatment as an efficient procedure for gluten content reduction. Foods.

[107] Li X., Wen Y., Zhang J., Ma D., Zhang J., An Y., Song X., Ren X., Zhang W. (2022). Effects of non-thermal plasma treating wheat kernel on the physicochemical properties of wheat flour and the quality of fresh wet noodles. Int. J. Food Sci. Technol..

[108] Kumar V., Rani A., Hussain L., Jha P., Pal V., Petwal V.C., Dwivedi J. (2017). Impact of Electron Beam on Storage Protein Subunits, In Vitro Protein Digestibility and Trypsin Inhibitor Content in Soybean Seeds. Food Bioprocess Technol..

[109] Zhang H., Chen G., Liu M., Mei X., Yu Q., Kan J. (2020). Effects of multi-frequency ultrasound on physicochemical properties, structural characteristics of gluten protein and the quality of noodle. Ultrason. Sonochem..

[110] Zhang K., Wen Q., Li T., Wang Y., Zhang Y., Luo D. (2022). Comparative study of the effects of ultrasonic power on the structure and functional properties of gliadin in wheat and green wheat. J. Food Sci..

[111] Zadeike D., Cipkute K., Cizeikiene D. (2025). Effects of Co-Fermentation with Lactic Acid Bacteria and Yeast on Gliadin Degradation in Whole-Wheat Sourdough. Fermentation.

[112] Wang L., Zhang Y., Xu F., Chen J. (2022). Effects of ultrasound-assisted resting on the tensile properties and gluten network formation of wheat noodle dough. J. Food Meas. Charact..

[113] Kumar A., Rani P., Purohit S.R., Rao P.S. (2020). Effect of ultraviolet irradiation on wheat (*Triticum aestivum*) flour: Study on protein modification and changes in quality attributes. J. Cereal Sci..

[114] Cai M., Kadam A., House J.D., Koksel F. (2026). High-moisture extrusion modifies texture and improves nutritional value of sunflower meal-pea protein meat analogues. Appl. Food Res..

[115] Lewko P., Wójtowicz A., Kamiński D.M. (2024). The Influence of Processing Using Conventional and Hybrid Methods on the Composition, Polysaccharide Profiles and Selected Properties of Wheat Flour Enriched with Baking Enzymes. Foods.

[116] Zhang C., Yang Y.-H., Zhao X.-D., Zhang L., Li Q., Wu C., Ding X., Qian J.-Y. (2021). Assessment of impact of pulsed electric field on functional, rheological and structural properties of vital wheat gluten. LWT.

[117] Page M.J., McKenzie J.E., Bossuyt P.M., Boutron I., Hoffmann T.C., Mulrow C.D., Shamseer L., Tetzlaff J.M., Akl E.A., Brennan S.E. (2021). The PRISMA 2020 statement: An updated guideline for reporting systematic reviews. BMJ.

