# Supplementary material for: Emerging Processing Technologies for Modulating Allergenic Proteins in Legumes and Gluten-Containing Cereals: A Systematic Review
Source: Molecules. 2026 Sep 27;31(19):3438. doi: 10.3390/molecules31193438 (PMC13666922; doi:10.3390/molecules31193438)
Supplement: Supplementary file 1 [file molecules-31-03438-s001.zip › PRISMA_2020_checklist.pdf]

PRISMA 2020 Checklist

Section and Topic	Item #	Checklist item	Location where item is reported
**TITLE**			
Title	1	Identify the report as a systematic review.	Page 1, lines 2–4
**ABSTRACT**			
Abstract	2	See the PRISMA 2020 for Abstracts checklist.	Page 1-2, lines 26–48
**INTRODUCTION**			
Rationale	3	Describe the rationale for the review in the context of existing knowledge.	Page 2-3, lines 137–146
Objectives	4	Provide an explicit statement of the objective(s) or question(s) the review addresses.	Page 2, lines 147–155
**METHODS**			
Eligibility criteria	5	Specify the inclusion and exclusion criteria for the review and how studies were grouped for the syntheses.	Section 2.2, page 5, lines 215–233; Section 2.3, pages 6-7, lines 278–293
Information sources	6	Specify all databases, registers, websites, organisations, reference lists and other sources searched or consulted to identify studies. Specify the date when each source was last searched or consulted.	Section 2.1, page 4, lines 174–180
Search strategy	7	Present the full search strategies for all databases, registers and websites, including any filters and limits used.	Section 2.1, pages 4–5, lines 174–199 (full Scopus strategy and Web of Science adaptation described)
Selection process	8	Specify the methods used to decide whether a study met the inclusion criteria of the review, including how many reviewers screened each record and each report retrieved, whether they worked independently, and if applicable, details of automation tools used in the process.	Section 2.2, page 5, lines 201–212 and 234-248
Data collection process	9	Specify the methods used to collect data from reports, including how many reviewers collected data from each report, whether they worked independently, any processes for obtaining or confirming data from study investigators, and if applicable, details of automation tools used in the process.	Section 2.3, page 6, lines 252–258
Data items	10a	List and define all outcomes for which data were sought. Specify whether all results that were compatible with each outcome domain in each study were sought (e.g. for all measures, time points, analyses), and if not, the methods used to decide which results to collect.	Section 2.1, page 4, lines 156–161; Section 2.3, page 6, lines 259–277
	10b	List and define all other variables for which data were sought (e.g. participant and intervention characteristics, funding sources). Describe any assumptions made about any missing or unclear information.	Section 2.3, page 6, lines 252–258
Study risk of bias assessment	11	Specify the methods used to assess risk of bias in the included studies, including details of the tool(s) used, how many reviewers assessed each study and whether they worked independently, and if applicable, details of automation tools used in the process.	Section 2.2, page 5, lines 234–248
Effect measures	12	Specify for each outcome the effect measure(s) (e.g. risk ratio, mean difference) used in the synthesis or presentation of results.	Section 2.3, pages 6–7, lines 270–286
Synthesis methods	13a	Describe the processes used to decide which studies were eligible for each synthesis (e.g. tabulating the study intervention characteristics and comparing against the planned groups for each synthesis (item #5)).	Section 2.3, pages 6-7, lines 278–294
	13b	Describe any methods required to prepare the data for presentation or synthesis, such as handling of missing summary statistics, or data conversions.	Section 2.3, pages 6–7, lines 270–294

PRISMA 2020 Checklist

Section and Topic	Item #	Checklist item	Location where item is reported
	13c	Describe any methods used to tabulate or visually display results of individual studies and syntheses.	Section 2.3, page 7, lines 287–301; Supplementary Tables S1–S2
	13d	Describe any methods used to synthesize results and provide a rationale for the choice(s). If meta-analysis was performed, describe the model(s), method(s) to identify the presence and extent of statistical heterogeneity, and software package(s) used.	Section 2.3, page 7, lines 277–293 (narrative and comparative synthesis; no meta-analysis)
	13e	Describe any methods used to explore possible causes of heterogeneity among study results (e.g. subgroup analysis, meta-regression).	Section 2.3, pages 6-7, lines 278–286 (heterogeneity addressed narratively by matrix, technology, molecular changes, assessment type, and response direction; no meta-regression)
	13f	Describe any sensitivity analyses conducted to assess robustness of the synthesized results.	Not reported: no sensitivity analysis is described in the manuscript
Reporting bias assessment	14	Describe any methods used to assess risk of bias due to missing results in a synthesis (arising from reporting biases).	Not applicable as a formal assessment: no reporting-bias analysis is described. Selective-reporting limitations are acknowledged in Section 2.2, page 5, lines 242–248
Certainty assessment	15	Describe any methods used to assess certainty (or confidence) in the body of evidence for an outcome.	Not applicable: no formal certainty-of-evidence framework was applied. Section 2.2, page 5, lines 245–248
**RESULTS**			
Study selection	16a	Describe the results of the search and selection process, from the number of records identified in the search to the number of studies included in the review, ideally using a flow diagram.	Section 2.2, page 5, lines 201–213; Figure 1, page 6, line 250
	16b	Cite studies that might appear to meet the inclusion criteria, but which were excluded, and explain why they were excluded.	Section 2.2, page 5, lines 201–241
Study characteristics	17	Cite each included study and present its characteristics.	Supplementary Tables S1–S2; Tables 1–10
Risk of bias in studies	18	Present assessments of risk of bias for each included study.	Not applicable: no formal risk-of-bias assessment was performed. Section 2.2, page 5, lines 234–248
Results of individual studies	19	For all outcomes, present, for each study: (a) summary statistics for each group (where appropriate) and (b) an effect estimate and its precision (e.g. confidence/credible interval), ideally using structured tables or plots.	Sections 3.4.1–3.4.8, pages 15–43, Tables 3–10
Results of syntheses	20a	For each synthesis, briefly summarise the characteristics and risk of bias among contributing studies.	Sections 3.1–3.5, pages 7–45, Tables 1–10; no formal risk-of-bias assessment was conducted
	20b	Present results of all statistical syntheses conducted. If meta-analysis was done, present for each the summary estimate and its precision (e.g. confidence/credible interval) and measures of statistical heterogeneity. If comparing groups, describe the direction of the effect.	Not applicable: no meta-analysis or quantitative statistical synthesis was performed. Narrative synthesis method: Section 2.3, page 7, lines 278–286
	20c	Present results of all investigations of possible causes of heterogeneity among study results.	No formal statistical heterogeneity analysis was conducted. Sources of heterogeneity are discussed narratively in Section 3.5, pages 43–45, lines 1266–1356, and Section 4, pages 45–47, lines 1357–1430
	20d	Present results of all sensitivity analyses conducted to assess the robustness of the synthesized results.	Not applicable, as no quantitative synthesis or statistical models were conducted. Consequently, it was not possible to perform sensitivity analyses to assess the robustness of the

PRISMA 2020 Checklist

Section and Topic	Item #	Checklist item	Location where item is reported
			results.
Reporting biases	21	Present assessments of risk of bias due to missing results (arising from reporting biases) for each synthesis assessed.	Not applicable: no formal reporting-bias assessment was conducted. Section 2.2, page 5, lines 242–248
Certainty of evidence	22	Present assessments of certainty (or confidence) in the body of evidence for each outcome assessed.	Not applicable: no formal certainty assessment was performed. Section 2.2, page 5, lines 245–248
**DISCUSSION**			
Discussion	23a	Provide a general interpretation of the results in the context of other evidence.	Section 3.5, pages 43–45, lines 1266–1356
	23b	Discuss any limitations of the evidence included in the review.	Section 4, pages 45–47, lines 1357–1430
	23c	Discuss any limitations of the review processes used.	Section 2.2, page 5, lines 242–248
	23d	Discuss implications of the results for practice, policy, and future research.	Section 4, pages 45–47, lines 1357–1430; Section 5, pages 46–47, lines 1431–1475
**OTHER INFORMATION**			
Registration and protocol	24a	Provide registration information for the review, including register name and registration number, or state that the review was not registered.	This review was not registered in any database.
	24b	Indicate where the review protocol can be accessed, or state that a protocol was not prepared.	No review protocol was prepared.
	24c	Describe and explain any amendments to information provided at registration or in the protocol.	Not applicable, as no protocol was registered or prepared.
Support	25	Describe sources of financial or non-financial support for the review, and the role of the funders or sponsors in the review.	Page 47, Funding, lines 1488–1489
Competing interests	26	Declare any competing interests of review authors.	Page 48, Conflicts of Interest, line 1501
Availability of data, code and other materials	27	Report which of the following are publicly available and where they can be found: template data collection forms; data extracted from included studies; data used for all analyses; analytic code; any other materials used in the review.	Page 47, Supplementary Materials, lines 1476–1479; Data Availability Statement, line 1490

From: Page MJ, McKenzie JE, Bossuyt PM, Boutron I, Hoffmann TC, Mulrow CD, et al. The PRISMA 2020 statement: an updated guideline for reporting systematic reviews. BMJ 2021;372:n71. doi: 10.1136/bmj.n71.
This work is licensed under CC BY 4.0. To view a copy of this license, visit https://creativecommons.org/licenses/by/4.0/
