# Supplementary material for: Emerging Processing Technologies for Modulating Allergenic Proteins in Legumes and Gluten-Containing Cereals: A Systematic Review
Source: Molecules. 2026 Sep 27;31(19):3438. doi: 10.3390/molecules31193438 (PMC13666922; doi:10.3390/molecules31193438)
Supplement: Supplementary file 1 [file molecules-31-03438-s001.zip › Table S1 (3).pdf]

Table S1. List of included studies

Title	Resource	Year	Country	Citation	Reference
Effect of ultra-high-pressure homogenization on structure and on rheological properties of soy protein-stabilized emulsions	Journal of Food Science	2002	France	173	[11]
The nutritive and functional properties of dry bean (Phaseolus vulgaris) as affected by gamma irradiation	Pakistan Journal of Nutrition	2009	Sudan	10	[12]
Flour Treatments Affect Gluten Protein Extractability, Secondary Structure, and Antibody Reactivity †	Foods	2024	USA	4	[15]
Effects of ultrasound-assisted punicalagin binding on the allergenicity reduction of soybean protein 7S	Food Chemistry	2025	China	1	[22]
High pressure processing of whole soymilk: Effect on allergenicity, anti-nutritional factor, lipoxygenase activity and E-nose-aroma characteristics	Food Chemistry Advances	2023	India	8	[23]
Effect of pulsed light on structure and immunoreactivity of gluten	Food Chemistry	2016	Belgium	44	[24]
Effect of thermal processing and γ-irradiation on allergenicity of legume proteins	Food and Chemical Toxicology	2012	India	11	[25]
Effect of ultraviolet irradiation on immunoreactivity and other protein characteristics of wheat gliadin in flour and convflatbread	Food Science and Applied Biotechnology	2023	Iraq	4	[26]
Molecular fragmentation of wheat-germ agglutinin induced by food irradiation reduces its allergenicity in sensitised mice	Food Chemistry	2012	Brazil	3	[31]
Changes in physico-chemical properties of native and toasted defatted soy flour on submission to electron beam radiation	Food and bioproducts processing	2017	India	10	[32]
Effects of ultrasonic treatment on removal of abundant proteins and enrichment of low-abundance proteins in defatted soybean meal by isopropanol	Biotechnology and Biotechnological Equipment	2016	China	10	[33]
Optimization of low-abundance protein extraction and abundant protein removal from defatted soybean meal	Journal of zhejiang university-science b	2017	China	5	[34]
Influence of γ-radiation on the structure and function of soybean trypsin inhibitor	Journal of Agricultural and Food Chemistry	2012	India	16	[35]

Effect of gamma irradiation on soybean allergen levels	Bioscience, Biotechnology and Biochemistry	2013	Japan	1	[36]
Detection of soybean antigenicity and reduction by twin-screw extrusion	Journal of the American Oil Chemists' Society	1994	Japan	90	[37]
Effect of microwave and conventional heat treatment on trypsin inhibitor activity and in vitro digestibility of edamame milk protein	Food bioengineering	2023	Canada	2	[38]
The ultrasound-treated soybean seeds improve edibility and nutritional quality of soybean sprouts	Food Research International	2015	China	148	[39]
Effects of gamma irradiation and salt stress on amino acids and protein fractions of two egyptian bread wheat (Triticum Aestivum L.) cultivars	Bangladesh Journal of Botany	2019	Egypt	1	[40]
The synergistic effect of heat treatment on alcalase-assisted hydrolysis of wheat gluten proteins: Functional and antioxidant properties	Journal of food processing and preservation	2017	Serbia	21	[41]
Characterization of sonicated gluten protein and subsequent rheological properties of model dough and noodles	Journal of the science of food and agriculture	2023	China	5	[42]
Characteristics of enzymatic hydrolysis of the wheat gluten proteins treated by ultrasound wave	Advanced Materials Research	2012	China	1	[43]
Effects of gamma-irradiation of wheat on gluten proteins	Journal of Cereal Science	1998	Turkey	32	[44]
Effect of ultrasound-assisted freezing on the textural characteristics of dough and the structural characterization of wheat gluten	Journal of food science and technology-mysore	2019	China	27	[45]
Modification of the structural and functional properties of wheat gluten protein using a planetary ball mill	Food chemistry	2021	China	53	[46]
Atmospheric Cold Plasma Treatment of Hard Wheat Flour and Soft Wheat Flour: Effects on Thiol Group Oxidation and Dough Rheology	Food and Bioprocess Technology	2026	USA	0	[47]
Modification of structure and mixing properties of wheat flour through high-pressure processing	Food Research International	2013	Australia	35	[48]

Functional properties of microwave-treated wheat gluten	European Food Research and Technology	2008	Turkey	51	[49]
Gluten Functionality Modification: The Effect of Enzymes and Ultrasound on the Structure of the Gliadin-Glutenin Complex and Gelling Properties	Molecules	2025	Lithuania	0	[50]
The effect of ultrasound on the functional properties of wheat gluten	Molecules	2011	China	104	[51]
Effect of low moisture extrusion on a pea protein isolate's expansion, solubility, molecular weight distribution and secondary structure as determined by Fourier Transform Infrared Spectroscopy (FTIR)	Journal of Food Engineering	2017	Australia	187	[52]
Effects of pulsed electric field and thermal treatment on the structural and techno-functional properties of mildly extracted pea proteins	LWT	2025	Belgium	0	[53]
Cold plasma jet-induced modifications in pea protein: A comparative study of gas-specific effects	Innov. Food Sci. Emerg. Technol.	2026	Canada	0	[54]
Effect of atmospheric cold plasma on structure, interfacial and emulsifying properties of Grass pea (Lathyrus sativus L.) protein isolate	Food Hydrocolloids	2020	Iran	157	[55]
Modulation of the emulsifying properties of pea globulin soluble aggregates by dynamic high-pressure fluidization	Innovative Food Science and Emerging Technologies	2018	France	69	[56]
Effect of high moisture extrusion cooking on protein-protein interactions of pea (Pisum sativum L.) protein isolates	International Journal of Food Science and Technology	2015	Germany	213	[57]
Effect of pH shift and high pressure homogenization on the structure and techno-functional properties of protein extracts from substandard peas	Current Research in Food Science	2026	Italy	0	[58]
Effect of sonication - cooking on the immunoreactivity of soy slurry from germinated soybeans	Food Innovation and Advances	2023	Canada	4	[59]
Comparative study of four physical approaches about allergenicity of soybean protein isolate for infant formula	Food and Agricultural Immunology	2016	China	56	[60]
Effects of Microwave and Ultrasound Assisted Extraction on the Recovery of Soy Proteins for Soy Allergen Detection	Journal of food science	2016	USA	2	[61]

Unveiling the regulatory mechanism of pulsed electric field modified instant soy milk powder: Exploring allergenicity, conformation, and epitopes	Food Chemistry	2025	China	2	[62]
Alteration of seed storage protein composition in soybean [Glycine max (L.) Merrill] mutant lines induced by γ-irradiation mutagenesis	Journal of Agricultural and Food Chemistry	2011	South Korea	14	[63]
Effect of pulsed electric field on the secondary structure and thermal properties of soy protein isolate	European Food Research and Technology	2011	Japan	107	[64]
Radiation induced variability of seed storage proteins in soybean [Glycine max (L.) Merrill]	Food Chemistry	2007	India	22	[65]
High pressure processing assisted enzymatic hydrolysis – An innovative approach for the reduction of soy immunoreactivity	Innovative Food Science and Emerging Technologies	2017	Germany	55	[66]
Soybean okara: Effect of ultrasound on compositional and emulsifying properties	International Journal of Food Science and Technology	2022	Argentina	10	[67]
Influence of ohmic heating on the structural and immunoreactive properties of soybean proteins	LWT	2021	Portugal	47	[68]
High-power sonication of soy proteins: Hydroxyl radicals and their effects on protein structure	Ultrasonics Sonochemistry	2020	USA	72	[69]
Enzymatic hydrolysis of extruded soybean meal at high substrate concentrations	Acta Periodica Technologica	2016	Russia	0	[70]
Effects of gamma irradiation on chemical composition, antinutritional factors, ruminal degradation and in vitro protein digestibility of full-fat soybean	Asian-Australasian Journal of Animal Sciences	2009	Iran	21	[71]
Effects of ultrasound pre-treatment on the structure of β-conglycinin and glycinin and the antioxidant activity of their hydrolysates	Food Chemistry	2017	Brazil	149	[72]
Raman spectroscopy analysis of the effect of electrolysis treatment on the structure of soy protein isolate	Journal of food measurement and characterization	2021	Tunisia	29	[73]
Impact of Microwave Time on the Structure and Functional Properties of Glycosylated Soy 7S Globulins	Foods	2025	China	2	[74]
Atmospheric cold plasma treatment of soybean protein isolate: insights into the	Journal of Food Science	2021	China	116	[75]

structural, physicochemical, and allergenic characteristics					
The effects of dielectric barrier discharge plasma treatment on the structure and gelling properties of soy protein isolate	Journal of Henan University of Technology: Natural Science Edition	2022	China	3	[76]
Effect of gamma radiation on protein profile, protein fraction and solubility's of three oil seeds: Soybean, peanut and sesame	Notulae Botanicae Horti Agrobotanici Cluj-Napoca	2011	Egypt	58	[77]
Effects of hydrodynamic cavitation at different pH values on the physicochemical properties and aggregation behavior of soybean glycinin	Lwt-food science and technology	2022	China	7	[78]
Effect of high intensity ultrasound on physicochemical and functional properties of aggregated soybean β-conglycinin and glycinin	Food Hydrocolloids	**2015**	China	231	[79]
Oxidation induced by dielectric barrier discharge (DBD) plasma treatment reduces IgG/IgE binding capacity and improves the functionality of glycinin	Food chemistry	2021	USA	11	[80]
Antigenicity in soybean hypocotyls and its reduction by twin-screw extrusion	JAOCS, Journal of the American Oil Chemists' Society	2000	Japan	15	[81]
Influence of high pressure on conformational changes of soybean glycinin	Innovative Food Science and Emerging Technologies	2003	Japan	99	[82]
Effect of Microwave Drying on Protein Subunits and Functional Properties of Enzyme-assisted Aqueous Extraction Processing Protein Rich Bean Powder	Nongye Jixie Xuebao/Transactions of the Chinese Society for Agricultural Machinery	2018	China	4	[83]
Effect of extrusion cooking on structure and functional properties of pea and kidney bean proteins	Journal of the Science of Food and Agriculture	2000	Spain	130	[84]
Relationships between processing conditions and starch and protein modifications during extrusion-cooking of pea flour	Journal of the Science of Food and Agriculture	1994	France	20	[85]
Small Angle X-Ray Scattering Studies on the Effects of Ultra Shear Technology on Model Animal and Plant-Based Protein Solutions	Journal of Food Process Engineering	2025	USA	0	[86]
Structural and functional properties of Maillard reaction products of protein isolate	International journal of food properties	2017	China	41	[87]

(mung bean, Vigna radiate (L.)) with dextran					
Effects of pH-shifting and ultrasound on the allergenicity and physicochemical properties of mung bean protein: application in gellan gum-based complex gels	Food Chemistry	2025	Singapore	0	[88]
Effect of cold plasma treatment on the antigenicity of peanut allergen Ara h 1	Innovative Food Science and Emerging Technologies	2019	Ireland	96	[89]
Reduction of IgE Immunoreactivity of Whole Peanut (Arachis hypogaea L.) After Pulsed Light Illumination	Food and Bioprocess Technology	2014	USA	24	[90]
Effect of Ultrasound Treatment on the Physicochemical, Nutraceutical, and Functional Properties of Lupine Flour	Journal of agricultural science and technology	2021	Jordan	1	[91]
Impact of thermal and biotechnological processing on the bioaccessibility and allergenic peptide profile of white lupin (Lupinus albus)	Front. Nutr.	2026	Turkey	0	[92]
Antioxidant Activity of Peptide Fractions from Chickpea Globulin Obtained by Pulsed Ultrasound Pretreatment	Horticulturae	2023	Mexico	4	[93]
Lentil protein: impact of different extraction methods on structural and functional properties	Heliyon	2022	Brazil	36	[94]
Influence of high-intensity ultrasound on physicochemical and functional properties of a guamuchil Pithecellobium dulce (Roxb.) seed protein isolate	Ultrasonics Sonochemistry	2022	Mexico	41	[95]
Effects of high-intensity ultrasonic bath on the structure and antigenicity of gliadin	Journal of Henan University of Technology: Natural Science Edition	2023	China	3	[96]
Changes in antigenicity of gliadin from medium flour by autoclave and microwave treatments	Journal of the Korean Society of Food Science and Nutrition	2011	USA	7	[97]
How microwave treatment of gluten affects its toxicity for celiac patients? A study on the effect of microwaves on the structure, conformation, functionality and immunogenicity of gluten	Food Chemistry	2019	Portugal	29	[98]
Study of the effects produced by gamma-irradiation of common wheat F 1 seeds using gliadins as genetic markers	Cytology and Genetics	2013	Ukraine	1	[99]

Gamma irradiation protect the developing wheat endosperm from oxidative damage by balancing the trade-off between the defence network and grains quality	Ecotoxicology and Environmental Safety	2019	India	8	[100]
The influence of gamma irradiation on the immunoreactivity of gliadin and wheat flour	European Food Research and Technology	2003	Poland	33	[101]
Influence of microwave heating on biological activities and electrophoretic pattern of albumin fraction of wheat grain	Cereal Chemistry	2010	Poland	9	[102]
Emulsifying Properties of Oat Protein/Casein Complex Prepared Using Atmospheric Cold Plasma with pH Shifting	Foods	2025	China	0	[103]
Enhancement of Pea-Oat Composite Protein Gel Properties Through Ultrasound Treatment Affects Structural and Functional Characteristics	Foods	2025	China	0	[104]
The effect of microwave treatment on the immunoreactivity of gliadin and wheat flour	European Food Research and Technology	2003	Poland	30	[105]
Synergistic effect of enzyme hydrolysis and microwave reactor pretreatment as an efficient procedure for gluten content reduction	Foods	2021	Serbia	11	[106]
Effects of non-thermal plasma treating wheat kernel on the physicochemical properties of wheat flour and the quality of fresh wet noodles	International Journal of Food Science and Technology	2022	China	15	[107]
Impact of Electron Beam on Storage Protein Subunits, In Vitro Protein Digestibility and Trypsin Inhibitor Content in Soybean Seeds	Food and Bioprocess Technology	2017	India	13	[108]
Effects of multi-frequency ultrasound on physicochemical properties, structural characteristics of gluten protein and the quality of noodle	Ultrasonics Sonochemistry	2020	China	111	[109]
Comparative study of the effects of ultrasonic power on the structure and functional properties of gliadin in wheat and green wheat	Journal of Food Science	2022	China	21	[110]
Effects of Co-Fermentation with Lactic Acid Bacteria and Yeast on Gliadin Degradation in Whole-Wheat Sourdough	Fermentation	2025	Lithuania	1	[111]
Effects of ultrasound-assisted resting on the tensile properties and gluten network formation of wheat noodle dough	Journal of Food Measurement and Characterization	2022	China	8	[112]

Effect of ultraviolet irradiation on wheat (Triticum aestivum) flour: Study on protein modification and changes in quality attributes	Journal of Cereal Science	2020	India	40	[113]
High-moisture extrusion modifies texture and improves nutritional value of sunflower meal-pea protein meat analogues	Applied Food Research	2026	Canada	0	[114]
The Influence of Processing Using Conventional and Hybrid Methods on the Composition, Polysaccharide Profiles and Selected Properties of Wheat Flour Enriched with Baking Enzymes	Foods	2024	Poland	2	[115]
Assessment of impact of pulsed electric field on functional, rheological and structural properties of vital wheat gluten	LWT	2021	China	38	[116]
